# Structurally exclusive Teneurin complexes orchestrate divergent programs in early cortical development

**DOI:** 10.1038/s41467-026-71619-1

**Published:** 2026-04-16

**Authors:** Miguel Berbeira-Santana, Claudia Peregrina, Kosuke Okuda, Jin Chuan Zhou, Maria Carrasquero-Ordaz, Amy V. Roberts, Anne E. Thomas, Evert Haanappel, Matthieu Chavent, Kamel el Omari, Lindsay A. Baker, Daniel T. Pederick, Els Pardon, Jan Steyaert, U. Valentin Nägerl, Daniel del Toro, Elena Seiradake

**Affiliations:** 1 Department of Biochemistry, University of Oxford, Oxford, UK; 2 Kavli Institute for Nanoscience Discovery, University of Oxford, Oxford, UK; 3 Department of Biomedical Sciences, Faculty of Medicine and Health Sciences, Institute of Neurosciences, IDIBAPS, CIBERNED, University of Barcelona, Barcelona, Spain; 4 Interdisciplinary Institute for Neuroscience, CNRS UMR 5297 and University of Bordeaux, Bordeaux, France; 5 Institut für Anatomie und Zellbiologie, Universitätsmedizin Göttingen, Göttingen, Germany; 6 Laboratoire de Microbiologie et Génétique Moléculaires, Centre de Biologie Intégrative, Université de Toulouse, CNRS, UPS, Toulouse, France; 7 Diamond Light Source, Harwell Science and Innovation Campus, Didcot, UK; 8 Department of Biology, Howard Hughes Medical Institute, Stanford University, Stanford, CA USA; 9 Structural Biology Brussels, Vrije Universiteit Brussel (VUB), Brussels, Belgium; 10 VIB-VUB Centre for Structural Biology, VIB, Brussels, Belgium

**Keywords:** Electron microscopy, Development of the nervous system

## Abstract

Cortical migration is a complex process in which neurons migrate along radial glial cells (RGC) to form functional layers. Teneurins (Ten1-4) play a role by interacting with Latrophilins (Lphn/ADGRL1-3). Teneurins are also known as cell adhesion molecules, but how homophilic and heterophilic Teneurin interactions are integrated is unknown. Here, single-particle-cryo-EM data of Ten2 shows that canonical Latrophilin-binding is sterically incompatible with Ten2-dimerisation, making these interactions exclusive. We engineered surface mutations that specifically disrupt Ten2-Ten2 or Ten2-Latrophilin interactions. These are transferrable to Ten4, suggesting conserved binding mechanisms. Proteomics, in-vivo-gene-editing and super-resolution-microscopy show that Ten4 is expressed along RGC fibres and that migrating neurons switch from low-to-high Ten4-expression. Ten4 expression is highest in the cortical plate where Ten4-Ten4 interactions reduce RGC-attachment. In the intermediate zone, Ten4-Latrophilin interactions are required to promote neuron-RGC association. The results show how Ten4 orchestrates different stages of cortical migration by using a structural/functional switch between high-affinity Lphn interactions and low-affinity homophilic interactions, underpinning the integration of distinct migration programmes.

## Abstract (web summary)

Here the authors show how Teneurin4 orchestrates different stages of cortical migration by using a structural/functional switch between high-affinity Latrophilin and lower affinity homophilic interactions, underpinning the integration of distinct migration programmes.

## Introduction

**Fig. 1:**
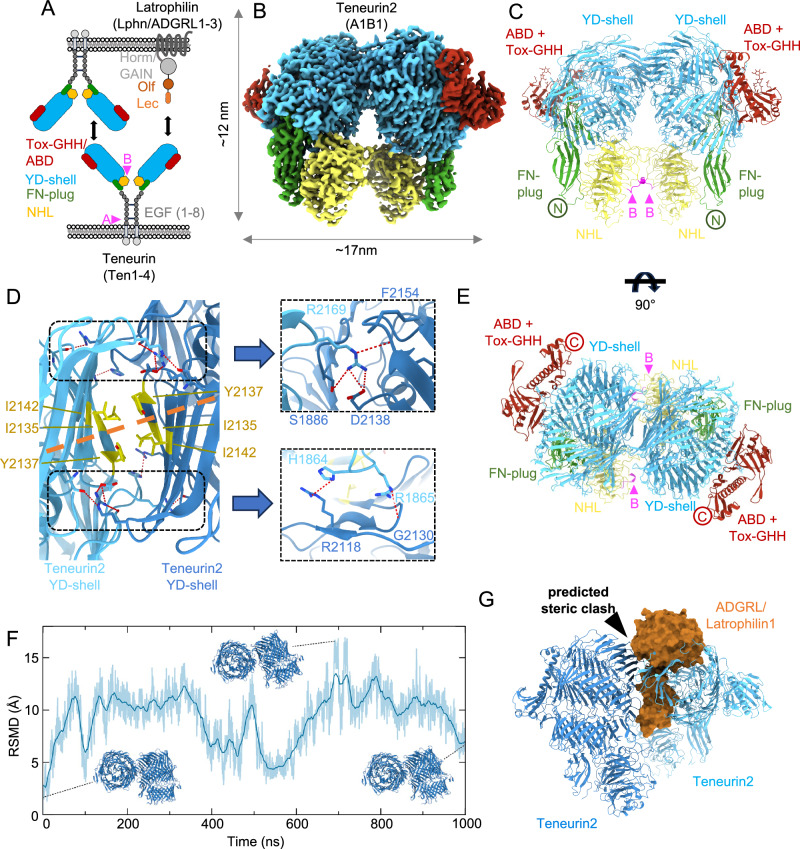
The murine Ten2 YD-shell forms a compact dimer that excludes Latrophilin-binding. **A** A cartoon overview showing that Teneurins engage in homophilic and heterophilic interactions. Domains of the Teneurin “superfold” and the Latrophilin N-terminal domains Lec and Olf are coloured separately. The alternatively spliced sites A and B are indicated by pink arrow heads in one copy of Teneurin. **B** CryoEM density map calculated for the ectodomain of murine Ten2 isoform A1B1 after refinement. Domains coloured as in (**A**). **C** Cartoon view of the Ten2 (A1B1) dimeric model, domains annotated and coloured as in (**A** and **B**). *N* termini are indicated. The location of the alternatively spliced site B (RNKDFRH in Ten2) is indicated and coloured magenta. **D** Left: zoomed view of the Ten2–Ten2 interface showing selected residue conformations after molecular dynamics (MD) simulation with backbone constraints. Key hydrophobic residues are shown as sticks, coloured in yellow. Residues providing stable hydrogen bonds or salt bridges are shown as blue sticks. Selected atoms are coloured by type: oxygen–red, nitrogen–blue. Hydrogen bonds are indicated by thin red dashed lines. The twofold pseudosymmetry axis is indicated as a thick orange dashed line. Right: close views on selected areas of the interface. **E** As (**C**), but rotated by 90 degrees around the *x*-axis. Visible C-termini are indicated. **F** We calculated the relative root mean squared deviations (RMSD) between the YD-shell backbones of the two Ten2 copies within the dimer of the A1B1 isoform, over the course of a 1000-nanosecond (ns) unconstrained MD simulation. The dark blue line shows average values across 10 sliding window frames. The light blue coloured area depicts the spread of the RMSD values of these frames for each time point. Snapshots of the model are shown for three time points. **G** Structural overlay of the Ten2 dimer (shades of blue) and the previously published structure of chicken Ten2 in complex with Lphn1 (orange, PDB 6SKA). The two interactions are sterically exclusive.

During cortical development, young pyramidal neurons migrate radially from the germinal layer in the (sub)ventricular zone (SVZ/VZ) and through the intermediate zone (IZ) to reach the cortical plate (CP), where they form cortical layers. During the initial phase of migration in the IZ, neurons are multipolar with short processes, characterized by random and low speed movements along the radial axis^1^. These neurons transition to a bipolar morphology in the upper portion of the IZ, close to the CP boundary, where they interact with radial-glial-cell (RGC) fibres and switch to faster, fibre-guided migration before settling into functional layers in the CP^2^. A cell surface receptor interaction network involving Teneurins has emerged as a key regulatory system of this migration^3^. Teneurins are large ( ~ 2800 amino acids) type II membrane receptors that evolved by a horizontal gene transfer event in which a bacterial toxin-like protein gene fused to the C-terminus of a eukaryotic membrane receptor^4^. Modern Teneurins are found in certain species of single-celled choanoflagellates and in bilaterians^4–8^. Mammals contain four isoforms (Ten1–4), all composed of an N-terminal intracellular domain, a single transmembrane helix, and a large ~2400 amino acid extracellular region. This large extracellular region is composed of a ~ 200 amino acid juxta-membrane domain followed by eight epidermal-growth-factor (EGF) domains, a cysteine rich region, a transthyretin-like domain, and a characteristic Teneurin “superfold” at the C-terminus (Fig. 1A). The superfold contains a spiralling beta-barrel tyrosine-aspartate repeat domain (YD-shell), which is decorated by four smaller domains: a fibronectin-like domain (FN-plug) and an “NCL-1, HT2A and Lin-41” (NHL) domain at the N-terminal end, and an antibiotic-binding domain fold (ABD) plus C-terminal DNAse-like domain (Tox-GHH)^7–11^ at the C-terminus. The amino acid chain folds into a knot-like structure: the C-terminal end of the YD-shell folds deep into the YD-shell lumen and then leads through its wall to form the ABD/Tox-GHH domains external to the YD-shell. Teneurins present two well-characterised alternative splicing sites located between the exons encoding for the EGF7 and EGF8 domains (splice site A) and in the NHL domain (splice site B). These splice sites regulate the homophilic (Ten–Ten) binding of Teneurins in cell-cell adhesion, with an insert required at either position A or B to promote cell-cell adhesion in vitro^9,12,13^. Teneurins also form disulphide bonded homodimers, using lone cysteines in the second and fifth EGF domains^14^. Their function as synaptic cell adhesion molecules was first described for fly Teneurins in olfactory synaptic partner matching^15–17^. A role for homophilic Ten3 interaction in murine hippocampal wiring is controversial^12,18,19^. While there is no definitive structural model of how Teneurins interact homophilically in trans (from one cell to another), structure-based hypotheses have been put forward for vertebrate Ten3, Ten4 and fly Ten-m^9–11^. These published Teneurin dimer structures are different from each other, and it is unclear if any represent ‘trans’ interactions in cell-cell contacts.

Teneurins also engage in heterophilic interactions, such as with the adhesion G-protein-coupled receptor family of Latrophilins (Lphn/ADGRL1–3, Fig. 1A)^20^, the only other known binding partner in cortical migration. Latrophilins function broadly in the nervous systems of vertebrates and invertebrates, with emerging roles in mechanosensation^21–25^, and neural wiring^18,20,23,26–31^. The Teneurin-Latrophilin complex further interacts with Fibronectin Leucine-Rich Transmembrane proteins (FLRTs)^3,32–35^ via Latrophilin. The Latrophilin extracellular region contains an N-terminal lectin (Lec) domain, followed by a linker, an olfactomedin (Olf) domain and hormone (Horm) plus GPCR-autoproteolysis-inducing (GAIN) domains. We and others recently showed that the Lec domain binds to the Teneurin YD-shell and that mutations in this interface reduce Teneurin-Latrophilin binding^3,36^. Latrophilins contain a well-characterised alternatively spliced site (SS) in the linker between the Lec and Olf domains, whose presence (SS+) or absence (SS−) might modulate Latrophilin functions^29,36^. Both Teneurins and Latrophilins are implicated in a range of neurodevelopmental diseases and other disorders such as attention deficit hyperactivity disorder, bipolar disorder and schizophrenia^37–41^.

The wealth of recent data, while detailing different protein interaction interfaces, has failed to address how Teneurins coordinate their dual functions as both homophilic binders and receptors for Latrophilins. The lack of molecular tools to unravel these functions in an in-vivo context where both are simultaneously expressed, has made previous analyses challenging, reflecting a general lack of information on how multifunctional receptors integrate their functions at the molecular-to-tissue levels. Here, we use single particle cryo-electron microscopy (cryo-EM) to demonstrate that the extracellular domain of murine Ten2 dimerises in an arrangement that is distinct from previously solved structures for other Teneurins. Structural comparison with previous results^3,36^ suggests that this arrangement clashes with Latrophilin Lec-binding at the canonical Teneurin-Latrophilin binding site. These structural insights, supported by molecular dynamics (MD) simulations, suggest that Teneurin homophilic and heterophilic interactions are structurally exclusive. We use these results to engineer Ten2 and Ten4 mutants that selectively inhibit either Teneurin-Teneurin or Teneurin-Latrophilin interactions in trans, and characterised a novel nanobody that selectively targets Ten4. Armed with these tools, we discovered that Ten4 is preferentially expressed along RGC fibres and that migrating neurons (MN) upregulate Ten4 as they migrate from the IZ to the CP. We find that Ten4-Latrophilin interactions are essential for association of neurons with RGCs in the IZ, while high Ten4-expressing cells engage in homophilic, cell-repulsive interactions in the CP, facilitating rapid migration along RGC fibres. The study illustrates how Ten4 uses the same interface to control both homophilic and heterophilic interactions sequentially to determine neuron-RGC attachment at different stages of cortical migration.

## Results

### Ten2 YD-shell homophilic binding clashes with Latrophilin Lec-binding

We produced full extracellular domains of the four major murine isoforms of Ten2 (A0B0, A1B0, A0B1, A1B1) using recombinant expression in mammalian cells, and performed single particle cryo-EM to determine the structures of Ten2 homodimers (Supplementary Fig. 1A). Maps after refinement have overall resolutions of ~2.5 Å (A0B0, A1B1, A1B0) −3.5 Å (A0B1) and are strikingly similar for the four isoforms (Supplementary Fig. 1B–E and Supplementary Table 1). Key differences are found in the area that corresponds to the NHL domain, where the B1 splice form is located. In this area, the maps of those isoforms that include the B1 splice insert (A0B1, A1B1) have the highest definition, while the A0B0 map is least defined. We performed further 3D refinement for the A1B1 and A0B0 isoforms to improve density for the NHL domain, using ~15% of the particles from each dataset (Fig. 1B and Supplementary Fig. 1F, G). We found that the map belonging to the A1B1 dataset has the best definition in the NHL region (Supplementary Fig. 1H), and so the models shown in most figures correspond to the A1B1 isoform of Ten2, unless indicated otherwise. Structural analysis resulted in a model that encompasses all of the superfold domains (Fig. 1C–E and Supplementary Table 2). Upstream domains, such as the EGF repeats and juxtamembrane regions, while present in the protein samples, are not resolved in any of the isoform datasets and may be flexible or flexibly linked. A structural alignment of the models derived for the four isoforms results in a root mean square deviation for C-alpha atoms (RMSD_Cα_) = ~ 0.3 Å, for residues C1019-E2770 (A1B1 notation, excluding NHL domain). For all four isoforms, the YD-shells belonging to two different chains come together at a ~ 75° angle, in a complex that is unlike other Ten dimer structures (Supplementary Fig. 1J, K). The surface area buried at this position in the interface is ~2100 Å^2^, and the high resolution of the map allows for confident placing of the side chains in this interface (Supplementary Fig. 1I). We performed an all-atom MD simulation for one microsecond to assess the stability of individual atomic interactions within the Ten2–Ten2 binding interface, using the A1B1 isoform model. Based on our established approach^3,42,43^, we imposed position restraints on backbone atoms (C, C_α_ and N), but not on side chain atoms. Taken together, the structure and atomistic simulation result suggest that the Ten2–Ten2 binding site is pseudosymmetric and consists of a hydrophobic core, which is flanked by four regions forming hydrogen bonds and salt bridges across the two monomers (Fig. 1D and Supplementary Fig. 2A–D). Two of these regions are dominated by R2169 coming from each of the monomers, and interacting with the nearby backbone oxygen of F2154, the side chain of D2138 and the backbone oxygen of S1886. The other two regions involve R1865 and R2118 from each monomer, interacting with the backbone oxygens of G2130 and side chains of H1864, respectively (Fig. 1D and Supplementary Fig. 2A).

To further assess the stability of the Ten2–Ten2 dimer, we performed an additional non-constrained simulation in which both monomers were allowed to move freely. We measured the RMSD between the backbones of the two YD-shells in the dimer, and although the dimer arrangement is stable, the RMSD values fluctuate slightly across the simulation, suggesting that the structure can “breathe” (Fig. 1F). Among the most stable hydrogen bond interactions are those mediated by Ten2 R2169, while other interactions (around R1865 and R2118) are transiently broken (Fig. 1D and Supplementary Fig. 2C, D). The central hydrophobic binding surface remains relatively stable during the simulation (Supplementary Fig. 2b). In summary, the interface may open up on one side, like a hinge, conceivably allowing other binding partners to interfere and displace a Teneurin monomer.

**Fig. 2:**
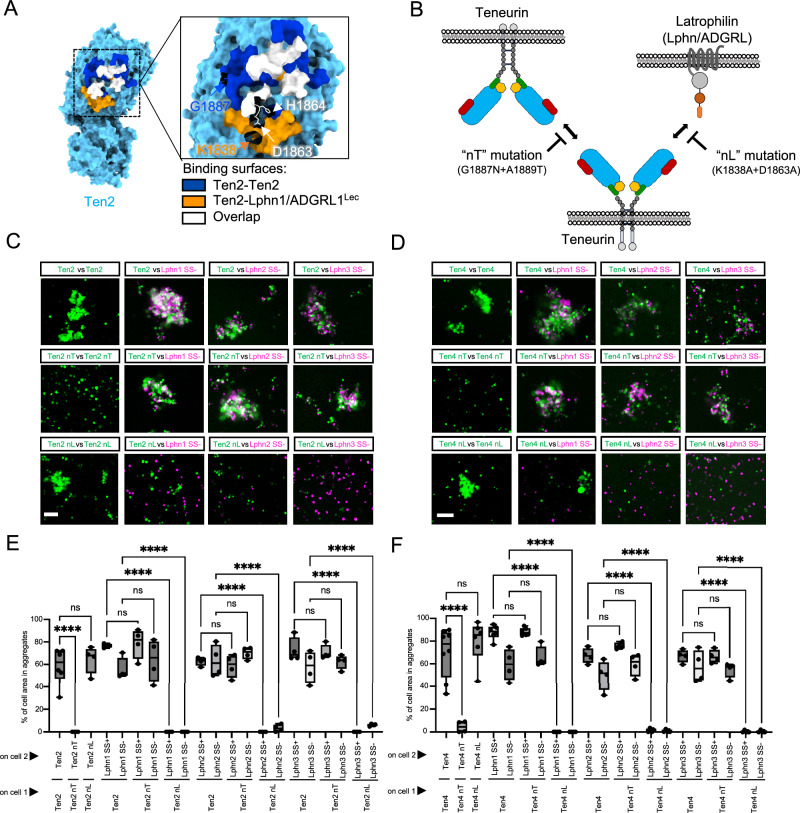
Design of Ten2 and Ten4 mutants with impaired in-trans binding capabilities for homophilic interaction (nT) and Lphn-binding (nL). **A** Surface view of one Ten2 monomer. The Ten2–Ten2 and Ten2-Lphn binding surfaces are coloured separately (dark blue and orange, respectively). Residues found in both binding sites are coloured in white. Interface assignment done with PISA^124^. **B** Summary of the properties of mutants nL and nT. **C**, **D** Representative images of selected cell aggregation experiments using Ten2 (**C**) and Ten4 mutants (**D**). Teneurin-expressing cells are shown in green, Latrophilin-expressing cells are shown in magenta. Two isoforms of Latrophilin were used (SS+ and SS−). **E**, **F** Quantification of cell aggregation experiments as those shown in (**C**, **D**). *N* = 4 experimental replicates, 6 pictures per replicate. n.s. not significant. ****p < 0.0001. One-way ANOVA test with Tukey’s post hoc analysis (**E**, **F**). Scale bars represent 100 μm (**C**, **D**). Box and whiskers plots (**E**, **F**) are defined as minimum data point to maximum data point, centre is median, percentiles are 25, 50 and 75. Source data are provided as a Source Data file.

Previously solved Ten2-Latrophilin complex crystal structures had revealed a binding site for the Lec domain of Latrophilin on the YD-shell of Ten2^3^. Structural comparison shows that this binding site and the homophilic interaction surface we discovered here are partially overlapping. This overlap results in a clash when the two structures are overlayed (Fig. 1G). Both binding sites localise to a similar surface region on the YD-shell, including also the R1865/R2118 binding area (Fig. 2A and Supplementary Fig. 2A–D). Therefore, the two interactions are likely structurally exclusive, such that Latrophilin binding could compete with Ten2 homophilic dimerisation. The dimer structures of Ten3 and Ten4 previously solved by cryo-EM^9,10^ do not engage this binding surface (Supplementary Fig. 1J, K).

### Structure-based protein engineering specifically disrupts homophilic or heterophilic interactions of Ten2 and Ten4

Using the structural data and MD simulations as a guide, we designed mutants aimed at specifically disrupting Teneurin-Teneurin or Teneurin-Latrophilin binding and assessed these using cell aggregation assays. G1887N + A1889T mutations, which introduce an N-linked glycosylation site at G1887 (Fig. 2B and Supplementary Fig. 2E–H), specifically disrupt aggregation between Ten2-expressing cells, without affecting Latrophilin binding (Fig. 2C, E and Supplementary Fig. 2I, K). Conversely, the mutations K1838A + D1863A disrupted the interaction with Latrophilins specifically, keeping the homophilic binding intact (Fig. 2C, E and Supplementary Fig. 2I–K). These mutants are termed “nT” and “nL”, as they are “non-Teneurin binding” and “non-Latrophilin binding”, respectively. The equivalent mutations R1881N + N1883T (nT) and R1832D + D1857A (nL) produced similar results for Ten4, showing that the interfaces are conserved in different Teneurin isoforms (Fig. 2B–F and Supplementary Fig. 2I, K). In agreement with previous studies, overexpression of wild-type Ten2 or Ten4 isoforms A1B0, A0B1 and A1B1 lead to robust cell-cell aggregation, which indicates effective *trans* interactions^3,9,12^ (Supplementary Fig. 2I, J), but we detected no mixed aggregates with cells expressing different Teneurin homologues (Supplementary Fig. 2L–N), suggesting that Teneurin homophilic interactions are homologue-specific. We also tested representative Ten3 isoforms (A0B0 and A1B1), but not Ten1 constructs, as these do not express at high enough levels at the cell surface of our in vitro systems, noting that Ten1 is also not very highly expressed during cortical migration (see mRNA expression data analysis below). The previously published chicken Ten2 “LT” mutation^3^, which is located in the shared region of the Latrophilin- and homophilic binding surfaces (H1864N + K1866T in Ten2 A1B1, H1858N + K1860T in Ten4 A1B1), abolishes both Teneurin-Teneurin and Teneurin-Latrophilin-dependent cell aggregation (Supplementary Fig. 3A–C), further confirming the overlapping of the two binding sites. Cell surface expression levels are similar for our mutants and wild-type proteins (Supplementary Fig. 3D–F).

We also used cell-based binding assays in which purified soluble Latrophilin1–3 Lec + Olf domains interact with cell surface Teneurins. These experiments confirm that Teneurin wild type and nT mutants bind Latrophilins, and that the nL mutants do not (Supplementary Fig. 3G–J). Vice versa, the wild type or nT mutants of purified Ten2 and Ten4 extracellular domains bound to cell surface Latrophilins, but not the Ten nL mutants (Supplementary Fig. 3K, L). The results confirm our conclusion that wild-type and nT proteins interact with Latrophilin, but not the nL mutants. Note that Teneurin-Teneurin trans interactions cannot be assessed by the latter cell-binding method, likely because robust binding requires avidity through cell surface presentation of the receptor.

The cell surface expression of Ten2 and Ten4 constructs used in these assays was validated using immunostaining and surface biotinylation methods (Supplementary Fig. 3D–F, I, J). The specific functionalities of the nT and nL mutants are summarised in Fig. 2B.

### Characterisation of a tetramerised anti-Ten4 nanobody: NanoTen4

**Fig. 3:**
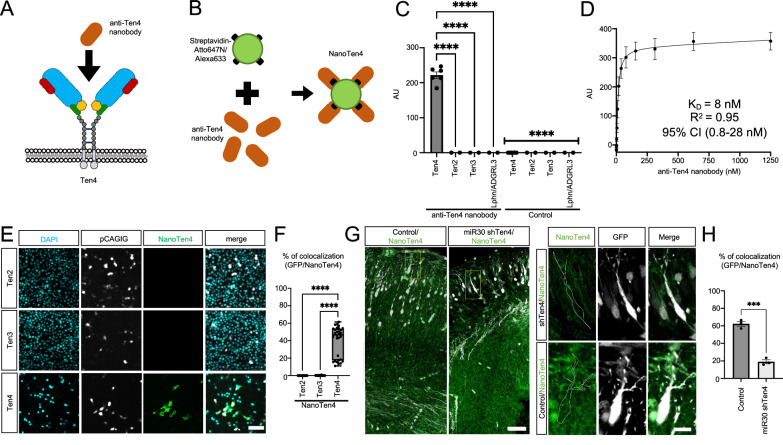
NanoTen4 specifically labels Ten4 in vitro and in brain tissue. **A** An anti-Ten4 nanobody was generated to target the Ten4 extracellular region (http://www.NanoSaurus.org entry SD-AW7W). **B** NanoTen4 is a tetramerised form of anti-Ten4 nanobody, using fluorescently labelled streptavidin. **C** ELISA assay shows binding of anti-Ten4 nanobody to Ten4, but not Ten2 or Ten3. *N* = 2 or more experimental replicates, 2 wells per condition per replicate. **D** Titration ELISA results suggest a Kd of ~8 nM for binding between the nanobody and Ten4 extracellular domain. *N* = 3 replicates, 4 wells per replicate. **E** A cell-based binding assay confirms that NanoTen4 (AlexaFluor-633, shown in green) binds to Ten4 but not Ten2 or Ten3 on cells (GFP+, shown in white). **F** Quantification of NanoTen4 cell-binding assays in (**E**). *N* = 3 experimental repeats, 10 areas per repeat. **G** E16.5 brain slices, immunolabelled with NanoTen4 (Atto647N, shown in green). Confocal microscopy analysis shows that NanoTen4-labelling is significantly lower in GFP+ neurons, in which Ten4 expression is knocked down with siRNA (see Fig. 5 and Supplementary Fig. 5). **H** Quantification of experiments shown in (**G**). *N* = 3 experimental repeats. n.s. not significant. ***p < 0.001 ****p < 0.0001. One-way ANOVA test with Tukey’s post hoc analysis (**C**, **F**). Two-tailed Unpaired Student’s *t*-test (**H**). Scale bar represents 100 μm (**E**), 50 μm (**G**, left), 10 μm (**G**, right/inset). All error bars shown are the SEM (**C**, **D**, **H**). Box and whiskers plots (**F**) are defined as minimum data point to maximum data point, centre is median, percentiles are 25, 50 and 75. Source data are provided as a Source Data file.

We produced an anti-Ten4 nanobody after immunising llamas with purified Ten4 extracellular domain protein (see “Methods” and Supplementary Fig. 4A, B). Using a previously established approach^42^, we biotinylated the nanobody at the C-terminus and used fluorescent Streptavidin (Atto647N/AlexaFluor^TM^633) for tetramerisation (Fig. 3A, B). The tetramerised nanobody is referred to as NanoTen4. We assessed the specificity and measured the affinity of the nanobody using enzyme-linked immunosorbent assays (ELISA) (Fig. 3C, D) and we used a cell-based binding assay to assess its specificity in a cellular context (Fig. 3E, F). To test the efficacy and specificity of NanoTen4 in tissue samples, we applied it to E16.5 murine brain slices containing electroporated cells (GFP control or Ten4 knock down, see details below). In these experiments, the knocked-down neurons bound NanoTen4 significantly less than control neurons (Fig. 3G, H). We also double-stained wild-type brain slices using an in situ hybridisation probe against Ten4 mRNA and found that its expression generally correlates with NanoTen4 labelling across different brain regions (Supplementary Fig. 4C, D).

### Ten4 expression is increased in the cortical plate and RGC fibres

**Fig. 4:**
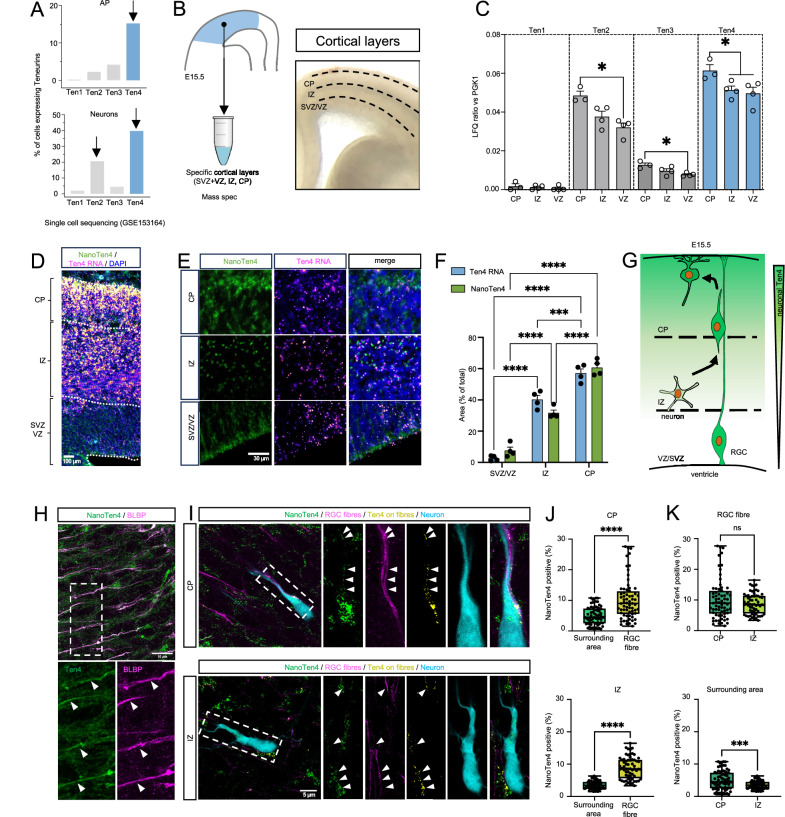
Ten4 is expressed in cortical neurons and progenitor cells, concentrated at RGC fibres, and upregulated in cortical plate neurons. **A** Percentage of cells expressing Ten1-4 mRNA in neurons and apical progenitors (AP) based on scRNA-seq data (GSE153164)^44^. **B** Diagram of the proteomics approach used to identify Teneurin expression levels in different cortical layers. CP cortical plate, IZ intermediate zone, (S)VZ (sub)ventricular zone. **C** Mass spectrometry results show Ten1–4 protein levels in the different cortical layers indicated in (**B**). *N* = 4 samples/group, 3 brains per sample. **D**, **E** In situ hybridisation highlights Ten4 mRNA (magenta) and NanoTen4 labelling for Ten4 protein (Atto647N, green) in E15.5 cortical tissue. **F** Quantification of data shown in (**E**). *N* = 3 sections pooled into 1 experimental replicate for a total of 4 experimental replicates (12 sections) from 2 biological replicates. **G** Summary diagram indicating that Ten4 expression increases from the (sub)ventricular zone to the cortical plate. **H** Confocal Airyscan images of E16.5 cortical plate tissue suggest spatial correlation between RGC fibres (AlexaFluor594, shown in magenta) and NanoTen4 (Atto647N, green) staining. **I** STED images of E16.5 cortical tissue labelled with NanoTen4 (Atto647N, green) and anti-BLBP (magenta), which labels RGC fibres. A confocal image of a GFP-positive neuron (cyan) is overlayed. We used sparse GFP labelling and therefore other neurons are not visible in this image. Yellow indicates Ten4 protein present on RGC fibres. Panels are a maximum projection of two slices of a Z-stack. **J**, **K** Quantification of data shown in (**I**), for cortical plate (CP) and intermediate zone (IZ). Areas within a 1.5 μm radius of the BLBP-positive fibres versus the surrounding tissue were analysed separately (“RGC fibre“), and the percentage of NanoTen4-positive pixels within this area versus the “surrounding areas” was quantified (**J**). **K** is based on the same quantification results as used in (**J**), but comparing values between the IZ and CP. *N* = 1 brain slice, for IZ *N* = 8 Z-stacks for a total of 64 images, for CP *N* = 7 Z-stacks for a total of 68 images. *p*-values (**K** upper graph = 0.1419, **K** lower graph = 0.0003) n.s. not significant. *p < 0.05 ****p < 0.0001. One-way ANOVA test with Tukey’s post hoc analysis (**C**, **F**). Two-tailed unpaired Student’s *t* test (**J**, **K**). Scale bars, 100 μm (**D**), 30 μm (**E**), 10 μm (**H**), and 5 μm (**I**). All error bars shown are the SEM (**C**, **F**). Box and whiskers plots (**J**, **K**) are defined as minimum data point to maximum data point, centre is median, percentiles are 25, 50 and 75. Source data are provided as a Source Data file.

Published single-cell RNA sequencing data^44^ suggests that Ten4 is the most abundantly expressed Teneurin in MN and apical progenitors (AP), including radial glial cells (RGC), at embryonic day 15 (E15, mid-gestation) (Fig. 4A and Supplementary Fig. 5A–C). To detect Ten4 protein levels in the different cortical layers (CP, IZ, and (sub)ventricular zone (SVZ/VZ)), we dissected wild-type brains and performed tissue mass-spectrometry of the different layers separately (Fig. 4B, C). We confirmed the precision of the dissection by analysing the presence of known markers for each layer (Supplementary Fig. 5D). We found that Ten4 is the highest expressing Teneurin in the cortex, which is in agreement with the scRNA-seq analysis. Within the E15.5 cortex, we found that Teneurins are most highly expressed in the CP (Fig. 4C). We also found that the three Latrophilins are present in all layers, especially the CP (Supplementary Fig. 5E). For better spatial precision, we assessed Ten4 protein and RNA levels also using NanoTen4 labelling and in situ *hybridisation* (Fig. 4D–G). We found increasing Ten4 protein and RNA levels from the (sub)ventricular zone to the CP, with the latter containing ~60% of the total nanobody and RNA staining. Interestingly, confocal Airyscan imaging showed that NanoTen4 staining overlaps with Brain Lipid Binding Protein (BLBP) staining, suggesting Ten4 expression along RGC fibres (Fig. 4H). To achieve higher resolution information on Ten4 protein localisation within the IZ and CP, where most Ten4 is expressed, we performed Stimulated Emission Depletion (STED) super-resolution microscopy on brain samples stained with NanoTen4 and anti-BLBP. We found that Ten4 is indeed enriched close to RGC fibres (within 1.5 μm) compared to surrounding regions (Fig. 4I–K and Supplementary Fig. 5F–H), both in the IZ and in the CP. However, compared to the IZ, more Ten4 is also expressed in non-RGC proximal areas of the CP (Fig. 4K). This result agrees with the proteomics and other imaging results, suggesting that more Ten4 is expressed by neurons in the CP (Fig. 4K).

Taken together, while Ten4 is preferentially expressed at RGC fibres across the CP and IZ, MN seem to upregulate Ten4 expression upon reaching the CP.

### Cortical migration requires homophilic and heterophilic Ten4 interactions at different stages

**Fig. 5:**
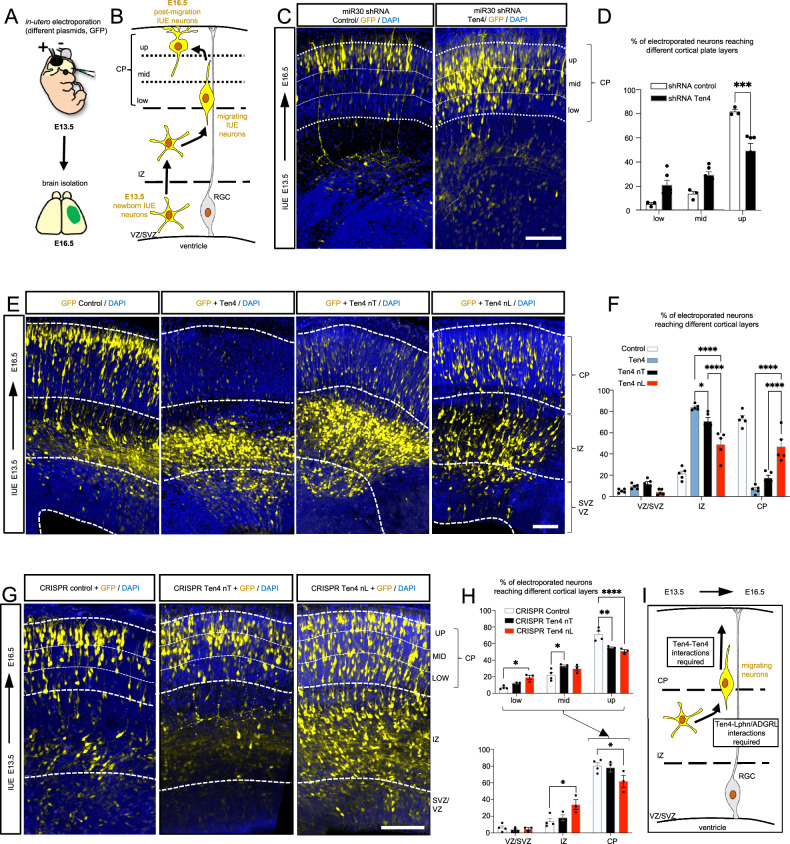
Ten4 directs different stages of cortical migration via homophilic and heterophilic interactions. **A** Schematic of in utero electroporation (IUE) performed at E13.5. **B** Diagram of radial migration during the course of an IUE experiment. **C** Coronal sections of an E16.5 electroporated murine cortex. Neurons were electroporated with pCACIG and pCAG-miR30 containing two shRNAs for murine Ten4. The cortical plate was subdivided into 3 bins (up, mid, and low), and the number of GFP+ neurons in each bin was quantified. **D** Quantification of experiments shown in (**C**). *N* = 3 electroporated brains (control), *N* = 6 (shRNA Ten4). **E** Coronal sections of an E16.5 cortex previously electroporated with pCAGIG (GFP) control plasmid or pCAG-Ten4-IRES-GFP, pCAG-Ten4nT-IRES-GFP or pCAG-Ten4nL-IRES-GFP expressing different Ten4 constructs in addition to GFP. **F** Quantification of the data shown in (**E**). *N* = 5 electroporated brains for all conditions. **G** Coronal sections of an E16.5 electroporated cortex. Neurons expressing GFP were modified with CRISPR/Cas9 to block Ten4–Ten4 interaction (Ten4 nT), Ten-Latrophilin interaction (Ten4 nL) or not modified in the *Tenm4* locus (CRISPR control). **H** Quantification of the data shown in (**H**). *N* = 3 electroporated brains. **I** Summary diagram. *p < 0.05, **p < 0.01, ***p < 0.001. We performed one-way ANOVA test with Bonferroni´s (**D**, **H**–top panel) or Tukey’s (**F**, **H**–bottom panel) post hoc analysis. Scale bars represent 100 μm (**C**, **E**, **G**). All error bars shown are the SEM (**D**, **F**, **H**). Source data are provided as a Source Data file.

To study the distinct roles of Ten4 interactions in vivo, we used an established murine *in-utero* electroporation model for genetic manipulation at embryonic age E13.5. The resulting tissues are analysed three days later at E16.5 (Fig. 5A, B). Using this method, we show that knock-down^3,42,45^ of Ten4 with two short-hairpin RNA (shRNA) delayed the migration of neurons within the CP, so that fewer neurons reached the upper layer compared to the control (Fig. 5C, D and Supplementary Fig. 6A–E). An experiment where we overexpressed wild-type Ten4 resulted in an even stronger delay, with most neurons remaining in the IZ, failing to enter the CP (Fig. 5E, F). This suggests that excessive levels of Ten4 are detrimental to cell migration, as has been observed for other receptors involved in this process^3,33^. However, introduction of the mutants nT and nL in these over-expression experiments significantly reduced the inhibitory effect of Ten4 over-expression. Of the two mutants, the nL mutant showed the most pronounced effect, with an apparent rescue of ~65% of cells reaching the CP, compared to the less pronounced but significant apparent rescue of ~24% for the nT mutant (Fig. 5E, F). Overexpression and knock-down experiments have certain limitations: a complete knock-out/knock-down of a multifunctional receptor abolishes all its functions, thereby making it difficult to unravel the specific roles of individual interactions. Also, the phenotype associated with over-expression experiments, even with structure-based mutants as controls, are often not physiological and only valid within a rescue framework. Therefore, we used a CRISPR/Cas9-based approach based on previously published methodology^3,46^ to introduce our structure-based mutations (nT and nL) in the endogenous *Tenm4* locus. In agreement with the above described results, we find that disrupting either Ten4 homophilic or heterophilic interactions affects neuronal migration (Fig. 5G, H), but as seen before, there were differences between the two mutants: the Ten4 nT mutant cells successfully entered the CP, but a significant number failed to reach the upper layer, indicating a delay in migration through the CP. In contrast, the Ten4 nL mutant cells accumulated in the IZ, at the boundary with the CP (Fig. 5G, H). To validate that the CRISPR-induced nT and nL mutations are present in our brain slices, we used BaseScope^TM^ to directly visualize edited mRNA in GFP^+^ neurons. We found that ~50% of the GFP^+^ neurons expressed the mutated transcript (Supplementary Fig. 6F–H). We further confirmed these results by amplicon sequencing to identify the genomic edits in electroporated tissue (Supplementary Fig. 6I, J).

In summary, these results suggest that Teneurin homo- and heterophilic interactions act at different points to direct migration through the IZ and CP. First, the Ten4-Latrophilin interaction promotes neuronal entry into the CP. Then, higher Ten4 expression and functional Ten4–Ten4 interactions are required for effective migration through the CP (Fig. 5I). Functional Ten4 homophilic interactions likely depend on the increased Ten4 levels present in neurons of the CP.

### Ten4 regulates neuron attachment to RGC fibre through distinct interactions

**Fig. 6:**
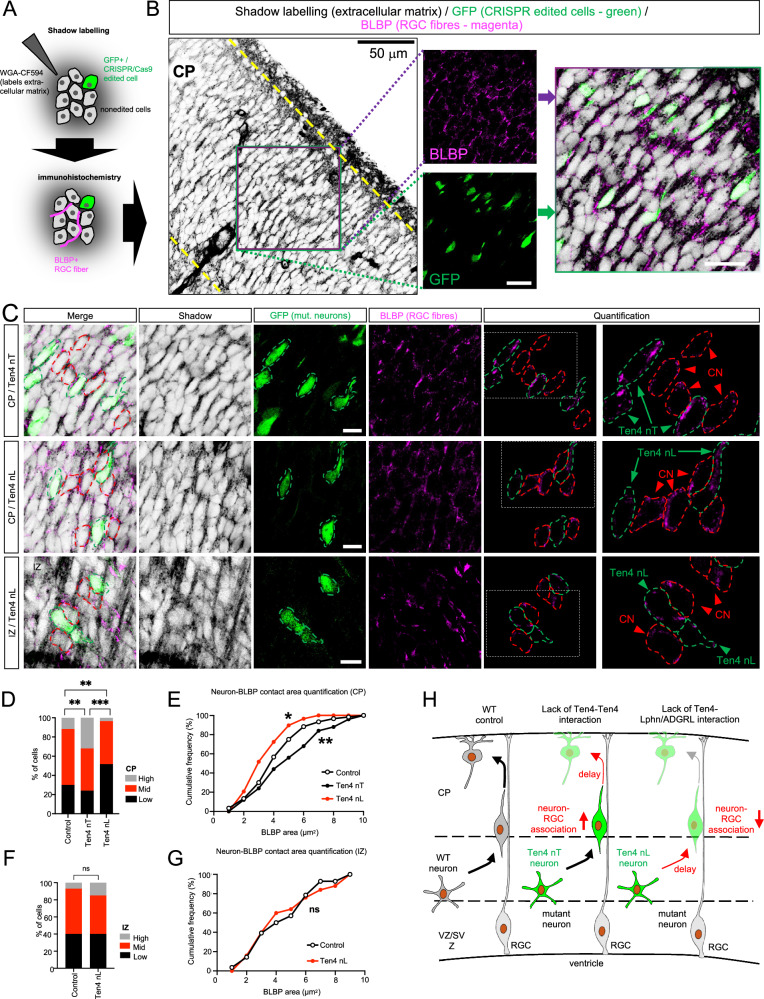
Ten4 regulates neuron-RGC association via two distinct mechanisms. **A** Diagram depicting the shadow and immunohistochemistry labelling strategy for brain slices collected after IUE using CRISPR/Cas9 reagents to target Tenm4 (as in Fig. 5G). Black = extracellular matrix (Shadow labelling), magenta = anti-BLBP, green = GFP-positive CRISPR-targeted neurons. Wild type neurons are white. **B** Example of a labelled brain slice with channels merged, following the strategy outlined in (**A**). **C** Shadow labelling (black) on electroporated coronal sections with GFP-positive CRISPR-targeted neurons (green) immunostained for BLBP (RGC fibres, magenta). Green dashed lines indicate the contour area assigned to each GFP-positive neuron, whereas red dashed lines represent the contour of adjacent control neurons identified through shadow imaging (white neurons). BLBP staining was quantified within each identified contour area (quantification panels). Area in white dashed rectangle is shown with higher magnification on the right. Red and green arrows indicate BLBP staining in control and CRISPR-targeted neurons, respectively. **D**–**G** Quantification of experiments as shown in (**C**). We expanded the outlined somata of cells by 1 μm and quantified the BLBP labelling within these areas for different cells (control, nL and nT). The neurons were separated into different populations depending on the levels of RGC staining in their vicinity: low (0–3.5 μm^2^), mid (3.5–6.5 μm^2^), and high (6.5–10 μm^2^). The plots display the fraction of cells and cumulative frequency distributions in the CP. *N* = 3 brains. H: Summary diagram showing the observed phenotypes. n.s. not significant. *p < 0.05, **p < 0.01, ***p < 0.001. Two-way Chi-square contingency analysis (**D**, **F**) and two-sided Kolmogorov–Smirnov test (**E**, **G**). Scale bars represent 50 μm (**B**) or 20 μm (**C**). Source data are provided as a Source Data file.

To visualise gene-edited neurons alongside non-edited wild-type neurons, we applied shadow imaging^47,48^ to our CRISPR/Cas9-edited samples (Fig. 6A, B and Supplementary Fig. 7A). The technique highlights the contours of all cells by staining the extracellular matrix within a tissue^47,48^. It is a powerful method for visualising all cells in an unbiased way and is compatible with immunostaining. We labelled RGC fibres with anti-BLBP, while GFP highlights CRISPR/Cas9-targeted neurons. In comparing gene-manipulated neurons with WT controls, we found that the introduction of the Ten4 nT and nL mutations change the association patterns with RGC fibres without affecting cell size in the CP (Fig. 6C–E and Supplementary Fig. 7B). Frequency distribution analysis showed that the fibre contact area of Ten4 nT neurons varies significantly more compared to the wild type control, whereas the distribution is narrower for Ten4 nL neurons. Neurons expressing Ten4 nT have increased contacts with RGC fibres compared to control neurons, while neurons expressing Ten4 nL have reduced contacts. We also analysed neurons located in the IZ, although as shown in Fig. 5, mainly Ten4 nL expressing neurons remain in the IZ at the point of analysis, while Ten4 nT cells have largely migrated into the CP at that point. We found no difference in cell size or RGC fibre contact area when comparing control and Ten4 nL cells in the IZ (Fig. 6C, F, G and Supplementary Fig. 7B). The results are consistent with previous studies indicating that migration through the IZ generally lacks defined polarity and is not along RGC fibres until the neurons switch to saltatory migration as they enter the CP^49–51^.

Taken together, these results suggest that different Ten4 interactions direct the association of neurons to RGC fibres in distinct, even opposing ways, and that they play a role at different time points: Latrophilin interactions play a major role initially, at the border to the CP, as neurons attach to the RGC fibres, while homophilic interactions regulate attachment and fast migration further up in the CP. Interfering with these distinct cellular mechanisms results in different migration phenotypes (Fig. 6H).

### Ten4 upregulation in neurons acts as a switch for migration

**Fig. 7:**
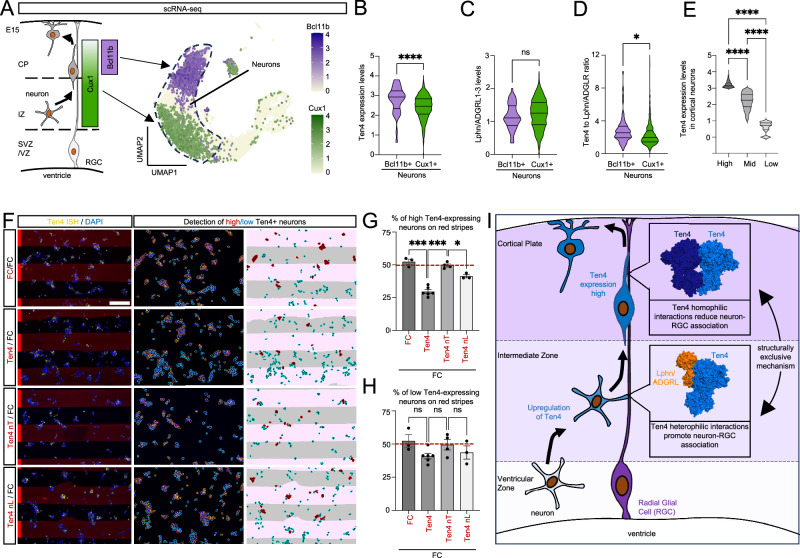
Homophilic Ten4 interaction triggers cell-repulsion in Ten4-expressing neurons. **A** Schematic showing where the markers Bcl11b and Cux1 are expressed in the cortex, and a UMAP derived from scRNA-seq analysis of cortical cells at E15.5 (GSE271794^52^). The cells are coloured depending on their expression of Bcl11b (purple) or Cux1 (green). **B** Levels of Ten4 expression in Bcl11b+ and Cux1+ neurons, after analysis of the data indicated in (**A**). The result shows that Ten4 is predominantly expressed in neurons of the cortical plate. **C** Levels of Latrophilin1–3 expression in Bcl11b+ and Cux1+ neurons. Latrophilins are not enriched in any particular section of migrating neurons. **D** Ratio of Ten4 to Latrophilin1–3 in Bcl11+ and Cux1+ neurons. Ten4 is enriched in Bcl11b+ neurons compared to Lphn/ADRGLs. **E** Cortical neurons exhibit distinct levels of Ten4 mRNA expression, here binned into high, mid and low. *N* = 7,540 neurons. **F** Representative images of a stripe assay in which E14.5 dissociated cortical neurons are challenged with WT, nT or nL Ten4 ectodomains. Neurons were grown on alternate stripes (red and black) containing Fc, Ten4, Ten4 nT or Ten4 nL. In-situ hybridisation was used to indicate Ten4 mRNA levels (yellow). Nuclear staining with DAPI is shown in blue. We used an automated detection pipeline to sort neurons into high and low Ten-expressing bins (see Supplementary Fig. 7I). **G**, **H** Quantification of experiments shown in (**F**), after binning. *N* = 3 or more experiments per condition. **I** Overview diagram summarising functions of Ten4 during cortical migration. n.s. not significant, *p < 0.05, **p < 0.01, ***p < 0.001, one-way ANOVA test with Tukey’s post hoc analysis (**E**, **G**, **H**). Two-tailed unpaired Student’s *t* test (**B**–**D**). Scale bar represents 100 μm (**F**). All error bars shown are the SEM (**G**, **H**). Source data are provided as a Source Data file.

Our results thus far have pointed to a phenotypic switch, where IZ neurons expressing lower levels of Ten4 react less to externally presented Ten4, compared to CP neurons expressing higher levels of Ten4. To confirm that cortical neurons express different levels of Ten4, we analysed our published scRNA-seq^52^ data derived from dissociated cortical neurons at E15.5 (Supplementary Fig. 8A). We show that Ten4 is indeed preferentially enriched in neurons populating the CP such as Bcl11b+ neurons (Fig. 7A, B and Supplementary Fig. 8B). Latrophilins are not preferentially enriched in these neurons (Fig. 7C, D). Also, we found different levels of Ten4 expression across the neuronal population (Fig. 7E), while the expression levels of Latrophilins were relatively constant (Supplementary Fig. 8C). The data analysis also shows that there is very low correlation between Ten4 expression and that of other Teneurins or Latrophilins (Supplementary Fig. 8D, E). Next, we tested whether high and low-level Ten4-expressing neurons react differently when challenged with external Ten4. We used dissociated cortical neurons (E14.5) in a stripe assay to test whether high Ten4-expressing neurons react more to external Ten4 (Supplementary Fig. 8F, G). In these assays, migrating cells choose between surfaces containing purified Ten4 protein (WT, nT or nL) and neutral control protein (recombinant Fc fragment protein (Fc)). In-situ hybridization labelling of Ten4 mRNA revealed cells expressing high levels of Ten4 versus those expressing low levels (Fig. 7F and Supplementary Fig. 8G). Quantification of these experiments showed that wild type Ten4, but not the nT mutant, is repulsive for high Ten4-expressing neurons (Fig. 7F, G), suggesting that Ten4–Ten4 homophilic “*trans*“ interactions are repulsive in these neurons. This result is in agreement with an increase of RGC fibre attachment for neurons expressing Ten4 nT, in vivo (Fig. 6C–H), as these lack the ability to engage in homophilic Ten4–Ten4 interactions. We previously showed that neurons tend to aggregate over time in this assay^3^. High Ten4 expression did not significantly inhibit neuron–neuron aggregation compared to low Ten4 expression (Supplementary Fig. 8H, I). In line with our model in which low-level Ten4-expressing cells do not engage in trans homophilic Ten4 interactions, we found that low Ten4-expressing neurons do not react significantly to Ten4 (Fig. 7F, H), suggesting that *trans* Latrophilin signalling, if switched on by the externally presented Ten4, is not causing a repulsive effect in these neurons. Interestingly, endogenous Latrophilin interactions could still play a role in these neurons as the mutant Ten4 nL protein stripes are less repulsive than the wild type protein, although still more repulsive that the nT mutant protein. Also, although high-Ten4 expressing cells avoid wild-type Ten4 stripes compared to low-Ten4 expressing cells, there is no significant difference for high versus low-Ten4 expressing cells on Ten4 nL stripes (Supplementary Fig. 8J).

## Discussion

Brain development relies on the multiple activities of receptors and adhesion molecules that operate in a context-dependent way. Different complexes can form between receptors and their diverse ligands at different times or simultaneously in different cell-to-cell configurations. This presents a challenge to studying the functions of individual interactions in vivo. We have developed an integrative approach that leverages structural biology-based protein engineering and nanobody-based tools to unravel how neurons integrate different stages of radial migration using structurally exclusive Ten4 interactions (Fig. 7I). In contrast to previously determined dimer structures^9,10^, we found that Ten2 forms a distinct dimer using a binding site on the YD-shell, which overlaps with the previously determined Latrophilin-binding site (Fig. 1 and Supplementary Fig. 1). These results provide a satisfactory explanation for why some mutations in this area abolish both homophilic and Latrophilin-binding in trans (see ref. ^3^ and Supplementary Fig. 3A–E). They also, finally, shed light on the elusive molecular mechanisms that underpin Teneurin-Teneurin trans interactions. Interestingly, the same interface was not observed in the previously published Ten4 dimer structure^9^, even though our mutational analysis points to a conserved *trans*-binding mechanism for Ten2 and Ten4. An alignment of this previously published Ten4 dimer ^9^ and our structures is sterically compatible, forming a continuous array (Supplementary Fig. 1J), it is therefore conceivable that both are used to mediate different Ten4 functions, even simultaneously.

How cortical migration is orchestrated by different molecular interactions to allow neurons to migrate to their final targets is overall poorly understood. Here, we identify Ten4 as a key regulator during two important steps: Ten4 interacts with Latrophilin in a high-affinity complex to promote RGC-attachment during the transition from the IZ into the CP, while during subsequent RGC-dependent migration within the CP, Ten4 forms weaker homophilic interactions to reduce RGC attachment. These weaker interactions could regulate the traction needed during fast migration observed in this layer^1^. Indeed, a recent study shows that weak adhesions are important in cell migration^53^, whereas either excessive or too little cell-adhesion impair migration^54,55^. The functional switch we describe is underpinned by the dynamic expression of Ten4, i.e., neurons migrating into the CP upregulate Ten4 expression, and a structural switch in which Ten4–Ten4 and Ten4-Lphn binding are sterically exclusive. Our findings position Ten4 as a regulator of the attachment dynamics that neurons exert on RGC fibres as they migrate through the CP. Indeed, Ten4 regulates the activity of the focal adhesion kinase (FAK)^43^, which is required for proper interaction between neurons and RGC fibres^56^. Upregulation of factors involved in switching migration patterns has also been observed for other proteins. As neurons migrate through the CP, several axon guidance receptors are expressed, most notably members of the Eph/ephrin protein family, such as EphrinB1^57^, EphA7^58^, and EphB6^59^, which regulate the lateral dispersion and columnar distribution of neurons within this layer. Other semaphorins and their receptors, Plexins, are also enriched in the CP. For example, Sema7A/4D^60^, as well as Sema3E/PlxnD1^61^, control neuron lamination.

Further questions remain. Could Ten4 interactions also contribute to migration termination? Interestingly, a recent study identified a repulsive interaction between Sema6A, expressed in RGC fibres, and PlxnA2/A4, enriched in neurons reaching the top of the CP. This interaction acts as a termination signal by inducing permanent neuronal detachment from the RGC fibres^62,63^. Moreover, increased neuron–neuron interactions are thought to weaken neuron-RGC fibre contacts during the terminal phase of migration. Given that Ten4 is upregulated in neurons within the CP, it is conceivable that Ten4 could function in alternative topologies, such as mediating interactions between neurons, which are densely packed in this region. Such a mechanism has been described for integrins; high α3 integrin expression in the upper part of the CP reduces neuron-RGC attachment while enhancing neuron–neuron interactions^64^. Interestingly, previous studies suggest that Teneurins may function redundantly with integrins^65^. In future work, it will be interesting to assess the possible interplay between Teneurin homophilic signalling and classical adhesion molecules such as cadherins, which play key roles in cortical migration^66^, and to determine whether Ten4 always signals cell repulsion or promotes adhesion in different contexts.

The intricate interdependence between molecular-level and tissue-level events that we show here for Ten4, is reminiscent of Teneurin functions in hippocampal wiring. In this context, Teneurin-expressing axons first encounter high-expressing Latrophilin regions, before reaching high-Teneurin expressing areas where synapses are formed^18^. Although our stripe experiments with cortical neurons results show a cell-repulsive effect for homophilic Ten4-interactions, hippocampal axons expressing Ten3 preferentially target and synapse with Ten3-expressing dendrites, suggesting that homophilic Teneurin interactions promote cell-contacts in this context. Indeed, homophilic axonal Teneurin interactions were first described as promoting adhesion and synapse formation in different biological systems^12,15–18^. The results illustrate that Teneurin homophilic interactions function in context-dependent ways, likely eliciting different functions in axons compared to migrating cells. This has also been shown for Teneurin-Latrophilin interactions, where, within the same experiment, Ten2-Latrophilin interaction was repulsive for cortical cells, but not for cortical axons^3^. The signalling mechanisms that determine these different response modes are not understood, but our stripe assay data suggest that co-expressed Latrophilins could be required for the effective repulsive response of high Ten4-expressing cells to external Ten4. Intriguingly, migrating cortical neurons establish transient glutamatergic synapses with subplate neurons before entering the CP, and disruption of these early synapses causes cortical migration defects^51^. Therefore, Teneurins, Latrophilins, and their interaction partners could transiently function as “synaptic proteins”, also during cortical migration^67–71^. Whether they do so, and how this integrates with other functions during migration, is a stimulating question for future research. A multifaceted role in mediating context-dependent adhesion or repulsion has been observed also for other receptors functioning in both cell migration and synapse specification, such as the Eph/ephrin protein family^72,73^. As for Ten4, receptor concentrations can be important for Eph functions. In vitro assays have shown that low levels of EphA promote attraction^74^, whereas high levels induce repulsion^75^. A similar effect is observed during topographic mapping, where growth cones migrate toward the superior colliculus: low levels of ephrinA promote attraction, while high levels trigger repulsion and inhibit migration^76^. Beyond the cortex and hippocampus, Teneurins and Latrophilins are found in multiple areas of the developing brain^27,77–81^, as well as in adult tissues and cancers^82–85^. Mutations and risk variants of Ten4 have been implicated in various brain disorders, including bipolar disorder^86^, schizophrenia^40^, mood disorders^87^, early-onset Parkinson’s Disease^88^, essential tremor^89^, disrupted axonal guidance^90^, and impaired oligodendrocyte myelination^91^, a process that is thought to depend on Ten4 homophilic interactions^92^. Dysregulated expression of Ten4 and Ten2 in particular is implicated in several cancer types^93–102^. The mutants, nanobodies and concepts we have developed here will likely be relevant for the study of these tissues and could shed light on the associated diseases. The work introduces a robust methodological framework for understanding the role of molecular interactions withing complex tissue environments.

## Methods

### Mouse embryos

All mice (C57BL/6 background) were housed with a 12 h:12 h light:dark cycle and food/water available ad libitum. All animal experiments were used in accordance with the ethical guidelines (Declaration of Helsinki and NIH, publication no. 85–23, revised 1985, European Community Guidelines, and approved by the local ethical committee (University of Barcelona, 225/17 and Generalitat de Catalunya, 404/18).

### Primary cultures

Neurons were dissociated from cortices of E14.5 embryos and cultured on stripes as described previously^3^. Neurons were cultured for 1 day in vitro at 37 °C, 5% CO_2_ in Neurobasal^TM^-A medium (Invitrogen, Cat#A3582901) supplemented with B27 (GIBCO, Cat#17504044). Then, cells were fixed with 4% Paraformaldehyde (SIGMA-Aldrich, Cat#158127-100G) for 10 min followed by immunostaining.

### Cell lines

K562 suspension cells (ATCC; CCL-243; RRID: CVCL_0004) were cultured in RPMI-1640 media (LGC Standards, Cat#ATCC 30-2001) supplemented with 10% Fetal Bovine Serum (FBS) (GIBCO, Cat#10437028). HEK293T cells (ATCC; CRL-3216; RRID: CVCL_0063) were cultured in DMEM plus L-glutamine (Thermo Fischer Scientific, Cat#41966052) supplemented with 10% FBS (GIBCO, Cat#10437028), and 5% Non-Essential Amino Acids (Life Technologies, Cat#11140035). Cell lines were maintained in sterile conditions in a 37 °C, 5% CO_2_-incubator.

### Vectors and cloning

We cloned constructs of mouse Ten2 (Uniprot: A0A0A0MQB7) (Ten2 A0B0, A1B1, A1B0, A0B1: residues 1–2774*; Ten2^ecto^: residues 398–2774*; nT mutant: G1887N + A1889T; nL mutant: D1863A + K1838A; LT mutant: H1864N + K1866T), mouse Ten4 (Uniprot: Q3UHK6) (Ten4 A0B0, A1B1, A1B0, A0B1: residues 1–2771*; Ten4^ecto^: residues 364–2771*; nT mutant: R1881N + N1883T; nL mutant: D1857A + R1832D; LT mutant: H1858N + K1860T), Latrophilin1 (Uniprot: H7BX15) (ADGRL1 residues 22–1471), Latrophilin2 (Uniprot: A0A0G2JGM8) (ADGRL2 residues 1–1487), and Latrophilin3 (Uniprot: Q80TS3-3) (ADGRL3 residues 1–1543). We used previously published mouse Latrophilin1–3 constructs as indicated^3^, including Lphn/ADGRL1^Lec-Olf^ (residues: 29–395, SS+, pHL-Sec C-terminal eAvi), Lphn/ADGRL2^Lec-Olf^ (residues: 30–400, SS+, pHL-Sec C-terminal eAvi) and Lphn/ADGRL3^Lec-Olf^ residues: 82–466, SS+, pHL-Sec C-terminal eAvi). For protein expression, the Teneurin ectodomain constructs were cloned into the Age1-Kpn1 site of the pHL-Sec vector (Addgene; Cat#99845)^103^. For structural studies and biotinylated nanobody production, we use a pHL-Sec vector that contains an N-terminal secretion signal peptide, which is used instead of the native signal peptide of the proteins. The vector also contains a 6xHis tag and an Avi-tag (protein sequence: GLNDIEAQKIEWHE). For nanobody production in bacteria, we use a pMESy4 vector (GenBank KF415192) that contains a His-tag and EPEA-tag at the C-terminus. The nanobody was cloned behind a pelB pre-signal (MKYLLPTAAAGLLLLAAQPAMA) that directs the nanobodies to the periplasmic space. For the Teneurin ectodomains used in stripe assays, ELISAs and nanobody generation, we use a pHL-Sec vector that contains a TwinStrep tag in the N-terminus (protein sequence: SAWSHPQFEKGGGSGGGSGGSAWSHPQFEK). For cell binding, cell-based aggregation assays and functional assays, full length constructs were used, cloned into the Xho1-Not1 site of the pCAGIG vector (Addgene; Cat#11159)^45^. pCAGIG was modified to express mCherry instead of GFP, for certain experiments, and is then referred to as pCAGIC. Teneurin constructs were cloned to contain an HA tag at the C-terminus (protein sequence: YPYDVPDYA) in the pCAGIG vector, whilst the Latrophilin constructs contained a myc tag at the N-terminus (protein sequence: EQKLISEEDL) in the pCAGIC vector. In addition, the Latrophilin full length constructs contained a PTPmu secretion signal sequenced prior to the myc-tag. We cloned the shRNAs 1 (sequence: GCAGCTCTGGTTGGCATTTAT) and 8 (sequence: GCAGTACATCTTCGAGTTTGA) into the pCAG-miR30 vector (Addgene; Cat#14758). These were designed using the BLOCK-iT™ RNAi Designer tool (https://rnaidesigner.thermofisher.com/rnaiexpress/rnaiDesign.jsp). *amino acid numbering is in A1B1 notation. The oligonucleotides’ sequences are available in a Supplementary Data file.

### Protein expression and purification

For large scale protein expression of the Teneurin ectodomains and biotinylated nanobody, HEK293T cells were grown in supplemented DMEM and 10% FBS in 2125 cm^2^ roller bottles (Grenier Bio-One, Cat#681070). After reduction of FBS to 2%, the cells were transfected with DNA plasmid and polyethylene imine (PEI) (SIGMA-Aldrich; Cat#208727), mixed in a 2-to-1 (PEI to DNA) mg:mg ratio. Cells were left to express the protein for four days and then the media were harvested, clarified by centrifugation and filtered using 0.22 μm sterile filters (Starlab; Cat# S1120-8810). The filtered media was concentrated and buffer-exchanged to diafiltration buffer (1× PBS (Invitrogen; Cat#3002), 20 mM Tris-HCl pH = 7.5, 150 mM NaCl). Then this media was passed through a 5 ml HisTrap^TM^ HP column (Cytiva, #Cat17-5248-02) at a flow of 5 ml/min. The column was washed with wash buffer (20 mM Tris-HCl, 300 mM NaCl, 40 mM Imidazole (Sigma-Aldrich, Cat#I3386)) and the protein eluted with 20 mM Tris-HCl, 300 mM NaCl, 500 mM Imidazole. 450 μl of the centre peak fraction was then injected into a pre-equilibrated Superose^TM^ 6 Increase 10/300 GL (Cytiva; Cat#29091598) in 25 mM HEPES-HCl (SIGMA-Aldrich; Cat#7365-45-9) (pH = 7.5) and 300 mM NaCl running buffer. Elution fractions were collected, analysed using SDS-PAGE and the peak fractions were either used directly to prepare cryo-EM grids or frozen at −80 °C.

For the purification of biotinylated Avi-tagged nanobody, the same protocol was used as before but with some changes. The same DNA:PEI ratio for transfection was used, but 90% of the DNA (weight) corresponds to the nanobody-containing vector, and the remaining 10% was BirA-encoding vector. Roller bottles for production of biotinylated protein were supplemented with 0.625 ml of 200 mM biotin after transfection. Purification of the biotinylated nanobody was performed always at 4 °C except for size exclusion chromatography. The peak fractions from the HisTrap were injected into a pre-equilibrated Superdex200^TM^ Increase 10/300 GL (Cytiva; Cat# 28990944) in 20 mM Tris-HCl (SIGMA-Aldrich; Cat#7365-45-9) (pH = 7.5) and 200 mM NaCl running buffer. Elution fractions were collected, analysed using SDS-PAGE and the peak fractions were either used directly to prepare tetramerised nanobody or frozen at −80 °C. For the preparation of the tetramerised NanoTen4, these peak fractions were concentrated and mixed in a 1:8 molar ratio (Streptavidin:Nanobody) with either Streptavidin-Atto647N (Rockland Immunochemicals, Cat#S000-56) or Streptavidin-AlexaFluor^TM^633 (Invitrogen, Cat#S21375), and left at room temperature (RT) in the dark for 1 h. The tetramerised nanobody mixture was then purified using a pre-equilibrated Superdex200^TM^ Increase 10/300 GL (Cytiva; Cat# 28990944) in 20 mM Tris-HCl (SIGMA-Aldrich; Cat#7365-45-9)(pH = 7.5) and 200 mM NaCl running buffer. Elution fractions were collected, analysed using SDS-PAGE and the peak fractions were either used directly or frozen at −80 °C.

For the N-terminal TwinStrep Teneurin constructs, protein expression was performed in the same way as for their Avi-tagged counterparts. The harvested media was concentrated and buffer-exchanged into diafiltration buffer (1× PBS, 20 mM Tris-HCl pH = 8, 150 mM NaCl), passed through a 5 ml StrepTrap^TM^ XT column (Cytiva, #Cat29-4013-17) at a flow of 5 ml/min. The column was washed (50 mM Tris-HCl, 150 mM NaCl, pH = 8) and the protein was eluted with elution buffer (50 mM Tris-HCl, 150 mM NaCl, 50 mM biotin). 450 μl of the centre peak fraction was then injected into a pre-equilibrated Superose^TM^ 6 Increase 10/300 GL (Cytiva) in 25 mM HEPES-HCl (pH = 7.5) and 300 mM NaCl running buffer. Elution fractions were collected, analysed using SDS-PAGE and the peak fractions were frozen at −80 °C.

For the purification of anti-Teneurin4 nanobodies used for ELISA analysis and initial characterisation, WK6 bacterial cells (ATCC/Fisher Scientific, Cat# 50-238-2643) were transformed with the desired nanobody construct using heat shock and plated. A single colony was then grown overnight at 37 °C in 8 ml of LB medium (Merck, Cat#L3147) with ampicillin. Then 5 ml of the preculture was transferred to 500 ml of complete TB medium (AppliChem, Cat#A0974), incubated 2 h at 37 °C at 230 rpm until it reached an OD of ~0.8. The temperature was reduced to 21 °C and incubated for another hour. Protein synthesis was induced with 120 μM (final concentration) of IPTG (Sigma, Cat#I6758-1G) and cells were left to express protein for 16 h at 21 °C and 230 rpm. The cells were pelleted and resuspended with 24 ml ice-cold 30 mM Tris pH 7.5, 20% Sucrose, 2 mM EDTA. Cells were incubated on ice for 30 min and centrifuged for 20 min at 10,400 × *g* (fixed angle). The supernatant was saved and the cell pellet resuspended with 25 mL ice-cold 30 mM Tris pH 7.5, 5 mM MgSO_4_. Cells were incubated on ice for 20 min and centrifuged for 20 min at 10,400 × *g*. Both supernatants were mixed and the nanobodies purified as explained above for the biotinylated version.

### Cryo-EM grid preparation

Protein samples were used straight after Size Exclusion Chromatography to ensure no protein aggregates deposit onto the grids (A0B1, A0B0, A1B0), or after thawing (A1B1). All samples were cleared by centrifugation prior to grid freezing. For all data presented here, holey carbon Quantifoil® R 1.2/1.3 300 copper mesh grids (Agar Scientific; Cat#AGS143-2) were first plasma cleaned for 2 min in a Harrick Plasma Cleaner (Harrick Plasma, USA). 3 μl of protein sample was applied onto the grid and blotted manually with filter paper. The grid was then mounted onto a Vitrobot Mark IV (Thermo Fisher) operating at 4 °C (A0B0, A1B0, A0B1) or 21 °C (A1B1), 100% humidity. 3 μl of the protein sample was applied again, blotted away with times ranging from 2.5 to 4 s before plunge-freezing in liquid ethane.

### Cryo-EM data collection and processing of mTen2 A0B1 dimer

A total of 5212 movies (Table S1) were recorded using a Talos Arctica cryogenic electron microscope (Thermo Fisher) equipped with a Field Emission Gun (FEG), operating at 200 kV. Micrographs were recorded using a Falcon 4 direct electron detector (Thermo Fisher) at 150,000× magnification (pixel size of 0.94 Å/pix) with an accumulated dose of 40 e^−^/Å^−2^, and a defocus range of −1.5 to−2.75 μm. All processing was performed in cryoSPARC^104^. Movies were motion corrected using Patch Motion Correction and the CTF estimated using Patch CTF. An initial set of 4,975 particles were picked using Blob Picking, classified in 2D, and the best dimeric classes used as references for Template Picking all micrographs. The resulting 1,037,437 particles were extracted with a box size of 658 Å^2^ and binned by 2. Junk particles were removed through successive rounds of 2D classification. Two ab initio models were then generated with 221,220 “good”, unbinned particles, and further 3D classified using the Heterogeneous Refinement job. The best 3D class containing 116,348 particles was further refined using Non-Uniform Refinement^105^, while optimising per-particle defocus and per-group CTF parameters and with C2 symmetry applied. This yielded a final map of 3.48 Å (FSC = 0.143, as reported by cryoSPARC). Details can be found in Supplementary Fig. 1A.

### CryoEM data collection and processing of mTen2 A1B1, A1B0 and A0B0 dimers

Datasets for mTen2 A1B1, A1B0 and A0B0 dimers were all collected on a Titan Krios electron microscope (Thermo Fischer) using a nominal magnification of 105,000× (0.83 Å/pix) and operating at 300 kV. Movies were recorded using a K3 Summit direct electron detector with a Bioquantum energy filter (Gatan) (20 eV slit), an accumulated dose ranging between 41.5 and 42.3 e^−^/Å^-2^, and a defocus range of −1 to −2 μm. A total of 8975, 5136 and 6108 movies were recorded for mTen2 A1B1, A1B0 and A0B0, respectively (Table S1). For all three datasets, processing was carried out in cryoSPARC^102^. All movies were first motion corrected using Patch Motion Correction followed by CTF estimation with Patch CTF. 2D references generated either from previous pilot datasets or a subset of Blob Picked particles were used for Template Picking. 6,799,146, 3,672,845 and 4,649,892 particles were initially extracted for A1B1, A1B0 and A0B0 datasets respectively, all with a box size of 301 Å^2^, and junk particles discarded through multiple rounds of 2D classification. Three ab initio models were generated for each dataset with “cleaned” particles, which were further 3D classified through one or two rounds of Heterogeneous Refinement. The best 3D class contained 128,786, 167,798 and 260,530 particles for A1B1, A1B0 and A0B0, respectively. Those were further refined using Non-Uniform Refinement^69^, while optimising per-particle defocus and per-group CTF parameters as well as with C2 symmetry applied, yielding maps of 2.54, 2.55 and 2.54 Å respectively. For A1B1 and A0B0 datasets, particles contributing to each refined map were further sorted via 3D Classification into 10 classes. In each case, the class that had both NHL domains resolved was then refined using Non-Uniform Refinement^105^, resulting in a final resolution of 2.80 Å and 2.82 Å for A1B1 and A0B0 maps, respectively (FSC = 0.143, as reported by cryoSPARC). Details can be found in Supplementary Fig. 1A.

### Molecular dynamics simulations and hydrogen bond identification

To perform an all-atom molecular simulation of Teneurin, we followed the approach outlined by Lemkul^106^ that we used earlier to study the GPC3-Unc5 receptor complex^42,106^. Briefly, we built a simulation model of the Teneurin homodimer based on the structure obtained by cryo-electron microscopy (Ten2–Ten2 A1B1 dimer). We removed all glycosylations from the structure and kept the disulfide bridges. We then used the Amber ff14SB force field to model this structure. Because the Amber ff14SB force field lacked an improper dihedral interaction for the HisD protonation state (6 His residues out of a total of 82), we manually set the protonation state of all His residues to HisE. We embedded the protein in a cubic box with edges at least 2.0 nm away from the protein. We solvated the protein in TIP3P water, neutralized the system and added 150 mM NaCl. The resulting system had a box size of (22.4 nm)^3^ and contained 1098381 atoms. We have used GROMACS 2023.2^107^ to perform the simulations. During the entire simulations we imposed position restraints on all backbone atoms (C, C_α_ and N), but not on the side chains, to preserve the overall protein structure. We minimized the system using the steepest descent method, then carried out a 100 ps NVT equilibration followed by a 100 ps NPT equilibration and a 1 µs production run. In all these simulations we used a time step of 2 fs, we set the temperature at 310 K using the V-rescale thermostat^108^ with a time constant τ_T_ of 0.1 ps and we set the pressure at 1 bar using the Parrinello-Rahman barostat^109^ with a time constant of 2.0 ps and an isothermal compressibility of 4.5 × 10^−5 ^bar^−1^. We employed the MDAnalysis toolkit^110,111^ to analyse MD simulation trajectories. For contact analysis, we have used the Distance_array function between the centres of mass of residues with a cutoff of 8 Å. To identify hydrogen bonds between the Teneurin monomers, the HydrogenBondAnalysis tool^112^ provided in using a donor-acceptor distance cut-off of 3.0 Å and a cut-off angle of 150°. We used the aforementioned protocol, but without constraints on the backbone for 1 μs.

### Stripe assays and immunostaining

We prepared the stripe assays as previously described in ref. ^3^. 50 μg/ml of Fc recombinant protein (Jackson Immunoresearch; Cat#009-000-008; AB_2337046), Ten4 WT, Ten4 nT or Ten4 nL, were mixed with AlexaFluor^TM^ 647-conjugated (Thermo Fischer Scientific (Cat#A-11014; AB_1500628) anti-hFc antibody (Thermo Fisher Scientific; Cat#62-8400; AB_2337530) in PBS (Life Technologies; Cat#10010023). Proteins were injected into matrices (90 μm width) (17546017) and placed on 60 mm dishes, resulting in red fluorescent stripes. After 30 min incubation at 37 °C, dishes were washed with PBS and matrices removed. Dishes were coated with 50 μg/ml Fc or Ten4WT/nT/nL protein mixed with 150 μg/ml anti-hFc (Jackson ImmunoResearch, Cat#62-8400) for 30 min at 37 °C and washed with PBS. Stripes were further coated with 20 μg/ml Laminin in PBS overnight and washed with PBS next morning. Cortical neurons (E14.5) were cultured on the stripes in Neurobasal medium supplemented with B27. After 24 h neurons were fixed with 4% PFA in PBS for 10 min at RT and stained as explained below. The numbers of ISH-positive pixels on red or black stripes were quantified with ImageJ (version 2.9.0/1.53t)^113^ after sorting on low/mid/high Ten4 expressing cells using Cellprofiler ^114^. For both software programmes, we used custom-made automated macros, which are available upon request.

### RNA In situ hybridization (ISH) in cultured neurons

Cultured neurons on stripes were fixed in 4% PFA for 10 min and dehydrated with increasing ethanol concentrations solutions (50, 70, 100%) in PBS for 5 min at RT. Stripes were kept in 100% ethanol at −20 °C until further use. Stripes were pre-treated using the RNAscope Universal Pretreatment Kit (Advanced Cell Diagnostics; Cat#322380). RNA In Situ Hybridizations (ISH) were performed using the RNAscope Fluorescent Multiplex Reagent Kit (Advanced Cell Diagnostics; Cat#323100) according to manufacturer’s instructions. The target gene (Ten4) was detected with the probe: Mm-Tenm4-C1 (RNAscope; Cat#555491). Following ISH, nuclei were counterstained with DAPI before mounting. Images were acquired using a Zeiss LSM880 confocal laser scanning microscope or a THUNDER imager (Leica) using a 10× objective and processed with ImageJ software. The oligonucleotides’ sequences are available in a Supplementary Data file.

### In utero electroporation

In utero electroporation was performed at E13.5 with anesthetized C57BL/6 mice as previously described^3^. DNA plasmids were used at 2 µg/µl and mixed with 1% fast green (Sigma-Aldrich, final concentration 0.2%). Plasmids were injected into the ventricle with a pump-controlled micropipette. After injection, six 50 ms electric pulses were generated with electrodes confronting the uterus above the ventricle. The abdominal wall and skin were sewed, and the mice were kept until E16.5 embryonic stage. To knockdown Ten4 by shRNA in vivo, we used two shRNAs embedded in the pCAG-miR30 vector, with the following sequence: shRNA#1 (sequence: GCAGCTCTGGTTGGCATTTAT) and #8 (sequence: GCAGTACATCTTCGAGTTTGA). This shRNA was validated in HEK293T cells, by co-transfection with Ten4 followed by Western Blotting (see below). For the CRISPR-Cas9 editing of the endogenous Ten4 locus, we used the Alt-R HDR Design tool on the IDT website. Oligonucleotides and Cas9 were ordered from Integrated DNA Technologies (IDT) using the Alt-R design tools and reagents. This includes the transactivating CRISPR RNA (IDT #:238355667), scrambled negative control crRNA (IDT #238355668 and #238355669), and the targeted CRISPR RNA (crRNA) sequences. Ten4 nT mutant was generated by targeting Exon30 (position: 96892998) with the following sequence: ACCTGACCGGCGTGAACGTGACA. For Ten4 nL, we targeted Exon29 (position:96888889) with the following sequence: GTGTTCGGCAGAGACCTGAGA, and Exon 30 (position: 96892922) with: GACGCCCACAGGAAGTTCACCCTGAGGATCCTGTAC. We followed the IDT protocol, where tracrRNA (IDT #238355667) and crRNA (400 µM stock) are hybridized in a 1:1 molar solution at 95 °C for 5 min and held at RT for 10 min, resulting in the final guide RNA (gRNA). The final solution was composed of 1.5 µl of gRNA (200 µM Stock), 2 µl of HDR template (200uM), 1 µl CAS9 (IDT #1081058), 1 µl pCAGIG (0.6ug/l final concentration), 1 µl Fast Green (final 0.2%) to a final volume of 10 µl. This solution was incubated at 37 °C for 10 min before surgery. The oligonucleotides’ sequences are available in a Supplementary Data file.

### Mass spectrometry of dissected cortical layers

Fresh E15.5 mouse brains (*N* = 12 brains) were divided into 4 replicate groups and each replicate was then manually dissected in cold PBS under and Olympus SZX10 stereomicroscope to isolate the different cortical layers: VZ/SVZ, IZ and CP. Each sample for each replicate group was homogenized separately for 1 min at 4 °C with an electric homogenizer in 50 μl of the following lysis buffer: 50 mM Tris-HCL (pH 7.4), 150 mM NaCl, 2 mM EDTA, 1% Triton ×-100 and protease inhibitors (Roche, Cat#04693116001). Samples were incubated on ice for 20 min and centrifuged for 10 min at 845 × *g*. The supernatant was collected, and then samples were processed for mass spectrometry and analysed (MaxQuant run, Proteomic facility, Max Planck Institute of Biological Intelligence, Martinsried, Germany). All reported readings for protein readings were normalized against PGK (house-keeping gene). The mass spectrometry proteomics data have been deposited to the ProteomeXchange Consortium via the PRIDE partner repository with the dataset identifier PXD075073.

### Shadow imaging

Three electroporated brains, coming from different animals, per condition were dissected and fixed in 4% PFA overnight, then washed and stored in PBS. We processed the brains with a vibratome to produce 100 µm sections. These brain slices were incubated at 4 °C for 2.5 h and then at R/T for 1.5 h in PBS with 5 µg/mL Wheat Germ Agglutinin, CF®594 Conjugate (Biotium, Cat#29064-1). Then, they were washed three times with PBS for 30 min. Finally, they were mounted in Fluoromount-G Mounting Medium (Southernbiotech, Cat#0100-01) on a glass slide under a cover slip. For the CP, a standard research confocal microscope was used (Zeiss LSM880) with a 63× oil immersion objective (NA 1.4; Plan-Apochromat 63×/1.4 Oil DIC M27). Image acquisition was controlled by the microscope’s commercial software (ZEN 2.3 sp1). Fluorophores were selectively excited by different lasers at wavelengths of 488 nm for GFP (mutant neurons), 561 nm for extracellular label (shadow imaging), and 633 nm for BLBP immunofluorescence. Image stacks were acquired with a x-y pixel size of 220 nm, a z-step of 1.5 μm and pixel dwell time of 2.05 μs. The imaging of the IZ was done using another but similar confocal microscope (Leica DMI6000 TCS SP8 X), also equipped with a 63× oil immersion objective of the same numerical aperture (NA 1.4; HC PL APO CS2 63×/1.40 OIL). Image acquisition was controlled by the microscope’s commercial software (LAS X). Fluorophores were selectively excited by different lasers at wavelengths of 488 nm for GFP (mutant neurons), 594 nm for extracellular label (shadow imaging), and 647 nm for BLBP immunofluorescence. Image stacks were acquired with a x-y pixel size of 361 nm, a z-step of 1.5 μm and pixel dwell time of 3.16 μs.

Areas that contained GFP-positive and GFP-negative cells alongside BLBP signals were selected for analysis from three separate slices coming from different animals as independent replicates. To obtain a measure of cell size, we outlined the somata of GFP-positive (mutant) and GFP-negative (control) neurons and expressed the enclosed area in square micrometres (μm^2^). To assess the association between neurons (mutant and wild-type control) and RGC fibres labelled by immunofluorescence (antibody directed against BLBP), the images were thresholded using the threshold function in ImageJ. The threshold was consistently set to 1%, so only the most intense 1% of pixels were considered as part of our object of interest. These resulting areas served as a local measure of the RGC fibres. Using ImageJ’s automatic ROI enlargement function, we expanded the outlined somata of the cells by 2 μm. Then, we measured the overlap between the RGC fibres and the expanded somata using the ImageJ ROI Manager. To separate the neurons depending on low, mid, and high levels of RGC fibre attachment, we used the following intervals: 0–3.5 μm^2^ for low, 3.5–6.5 μm^2^ for mid, and 6.5–10 μm^2^ for high.

### Cell-based binding assay

HEK293T cells grown on coverslips were transfected using HA-tagged constructs in pCAGIG vector with 3 μg of DNA and 9 μl of PEI. Eighteen hours after transfections, cells were incubated with buffer (HBSS (Life Technologies; Cat#14170088) with 1% BSA and 10 mM HEPES (pH 7.5)) for 30 min on ice, and then with buffer containing 0.5 μg purified His-tagged protein per coverslip that was previously pre-clustered (30 min at RT) with anti-His (mouse; Thermo Fisher Scientific; Cat#372900; RRID: AB_2533309) in a 1:2 (protein:antibody) ratio (mass:mass) for 60 min on ice. Cells were then washed with PBS and fixed with 4% PFA for 20 min, and then washed using PBS supplemented with 50 mM ammonium chloride (SIGMA-Aldrich; Cat#326372-100 G). Cells were then incubated in the dark with anti-mouse-Cy3 (Invitrogen; Cat#A10521; AB_10373848) in a 1:7.5 (protein:antibody) ratio (mass:mass) in buffer for 60 min on ice. The cells were washed with PBS, stained with DAPI (0.1 μg/ml), washed with PBS and mQ water, and then mounted using Immu-Mount (Fischer Scientific; Cat#10622689) into SUPERFROST microscope slides (Fischer Scientific; Cat#12372098). For the in vitro validation of NanoTen4, the same protocol was used, but NanoTen4 was added to the cells for 120 min on ice and there was no secondary antibody step. Imaging for data analysis was done with a Nikon ECLIPSE TE2000-U inverted fluorescence microscope or a THUNDER Imager (Leica). Analysis was performed in ImageJ (version 1.54f)^113^ using the co-localisation macro used previously^33^. The co-localisation area of red pixels (soluble bound protein) with green pixels (transfected cells) was normalised against the area of the transfected cells and converted into a percentage. For the statistical analysis, we used a one-way ANOVA test, with a Tukey’s post-hoc test in Graphpad Prism (version 10 for MacOS, GraphPad Software, San Diego, California USA, www.graphpad.com). Significance was determined when *p* < 0.05. Pictures showed in Figures were imaged using a THUNDER Imager (Leica).

### shRNA in vitro validation

HEK293T were cultured in 6-well plates on coverslips. Twenty-four hours after culturing, the cells were co-transfected with shRNA (both #1 and 8 constructs) and full length Ten4 A1B1 in a 3:1 μg ratio with Fugene (Promega; Cat#E2693) (1:3 (v/m) ratio with total DNA). Cells were harvested 72 h after transfection, sonicated (2 pulses of 5 and 7 s, respectively on ice). Samples were loaded into a 3–8% Tris-Acetate gels (Thermo Fischer Scientific; Cat#EA03785BOX), run for 15 min at 150 V and then for 40 min at 180 V. The gels were transferred in the NuPAGE^®^ XCellII^TM^ Blot transfer module for 90 min at 30 V onto a nitrocellulose membrane (Cytiva, Cat#10600013), sandwiched with two blotting papers and five sponges, submerged in 1× NuPAGE^®^ Transfer Buffer (Invitrogen^TM^, Cat#NP00061) supplemented with 20% ethanol. To confirm successful transfer, the HiMark^TM^ Pre-stained Protein ladder (Invitrogen, Cat#LC5699) was used. After transferring, the membrane was blocked for 30 min using 3% BSA (w/v) (Invitrogen^TM^, Cat#A7906) in PBST (1× PBS with 0.1.% Tween20) and washed with PBST only for 30 min. Then the membranes were incubated in the primary antibody (mouse anti-HA (Sigma-Aldrich, Cat#H3663) prepared in PBST with 3% BSA in a 1:1000 dilution) solution for 1 h. After incubation, the membrane was washed for 30 min with PBST and incubated for 1 h with the secondary antibody solution (anti-Mouse IgG HRP (Sigma-Aldrich, Cat#A0168) diluted 1:10,000 in PBST with 3% BSA). Then the membrane was washed with PBST for 45 min and developed with the ECL^TM^ Western blotting detection reagents (Cytiva, Cat#RPN2106) and visualised using Amersham^TM^ Hyperfilm^TM^ ECL^TM^ films (Cytiva, Cat#28-9069-35). The Western Blots were quantified using ImageJ^113^ using the intensity of the construct band normalised by subtracting against the intensity of the non-transfected band for control purposes. For the statistical analysis, we used a one-way ANOVA test, with a Tukey’s post-hoc test in GraphPad Prism (version 10 for MacOS, GraphPad Software, San Diego, California, USA, https://www.graphpad.com/). Significance was determined when *p* < 0.05.

### Cell-cell aggregation assay

K562 suspension cells were cultured in RPMI-1640 media (no phenol red) (Invitrogen; Cat#11835030) supplemented with 10% FBS and 5% L-Glutamine. The cells were harvested by a 3 min spin at 200 × *g*, washed with PBS, spined again and resuspended in R buffer (Neon Transfection System 100 μL Kit; Thermo Fischer Scientific; Cat#MPK10025). Cells at a concentration of 2 × 10^7^ cells/ml were transfected with control pCAGIG/pCAGIC plasmids, or those coding for mTen2,4 or mADGRL1-3 constructs using the Neon transfection system for electroporation (Settings: 1450 V, 3 pulses, 10 ms). Eighteen hours after transfection, cells were harvested and used at a concentration of either 2 × 105 cells/ml or 4 × 105 cells/ml in aggregation media (Neurobasal-A media without phenol red (Thermo Fischer Scientific; Cat#12349015) supplemented with 2  mM L-glutamine (Life Technologies; Cat#25030-024), 10% FBS, 4% B-27 and 20 mM HEPES) in a 24-well plate. Cells were then left to aggregate at 37 °C, 5% CO_2_ and 250 rpm for 90 min. After the incubation, the cells were imaged directly in the 24-well plate after a slight shake using a Nikon ECLIPSE TE2000-U inverted fluorescence microscope. The total area of cells and the total area of the aggregates for each picture were calculated using the Analyze particle tool in ImageJ^113^. The threshold used to distinguish cells and aggregates was determined at 1284 μm^2^ (>3/4 cells). For the statistical analysis we used a one-way ANOVA test, with a Tukey’s post-hoc test in GraphPad Prism (version 10 for MacOS, GraphPad Software, San Diego, California, USA, www.graphpad.com). Significance was determined when *p* < 0.05. Pictures showed in Fig. 2 and Supplementary Fig. 2 were imaged using a THUNDER Imager (Leica).

### Cell surface expression in K562 cells

For surface staining, K562 cells were harvested eighteen hours after electroporation (performed as explained above) and cooled to 4 °C for 30 min. Cells were spined at 200 × *g* for 3 min (4 °C) and the media aspirated. This process of spinning and aspirating was performed in between all following steps. All the steps in this protocol are performed on non-permeabilised cells, so that the antibody only detects protein that has been trafficked to the cell surface, where the tag is exposed to the antibodies. We also perform all steps at 4 °C to abolish endocytosis. The cells were then incubated on ice with blocking buffer: HBSS with 1% BSA and 10 mM HEPES (pH 7.5) for 30 min on a shaker. For Ten2,4 expressing cells, cells were incubated for 1 h on ice on a shaker. We used anti-HA (mouse; SIGMA-Aldrich; Cat#H3663; RRID: AB_262051) antibody that was pre-clustered with secondary anti-Mouse antibody-Cy3 (Invitrogen; Cat#A10521; AB_10373848) for 40 min (ratio 1:7.5 for primary:secondary antibody) at RT in the dark. Cells were washed with PBS, fixed with 4% PFA for 20 min, washed with PBS with ammonium chloride and stained with DAPI (0.1 μg/ml) for five minutes on ice and shaking. Cells were then resuspended on 20 μl Immu-Mount and put on the centre of the slide. Finally, a rectangular coverslip was put on top of the resuspended cells. The cells were imaged on a using a Nikon ECLIPSE TE2000-U inverted fluorescence microscope (20× magnification). Colocalisation for the surface quantification was performed using ImageJ^113^ with the colocalisation tool and a macro that can be made available upon request. This colocalisation analysis compares the pixels in both channels (green (transfected cells) and red (anti-HA/Cy3 clustered antibodies)) and determines which pixels have a signal in both channels. A ratio of 10% between red and green channels was used. As the cells are non-permeabilised, the signal coming from the red channel (Cy3) can only come from the HA-tagged proteins on the cell surface, if present. The area of the pixels that are colocalised between both channels is the normalised by the total transfected cell area. For the statistical analysis, we used a one-way ANOVA test, with a Tukey’s post-hoc test in GraphPad Prism (version 10 for MacOS, GraphPad Software, San Diego, California, USA, www.graphpad.com). Significance was determined when *p* < 0.05.

### Cell surface expression in HEK293T cells

HEK293T cells grown on coverslips were transfected (24 h after seeding, at a confluence of 80%) using full length Teneurin constructs in pCAGIG vector with 3 μg of DNA and 9 μl of PEI. All the steps in this protocol are performed on non-permeabilised cells, so that the antibody only detects protein that has been trafficked to the cell surface, where the tag is exposed to the antibodies. We also perform all steps at 4 °C to abolish endocytosis. Eighteen hours post-transfection, cells were incubated with buffer (HBSS with 1% BSA and 10 mM HEPES (pH = 7.5)) for 30 min on ice and then with buffer containing 1 μg of mouse anti-HA antibody for 1 h. Cells were then washed with PBS and fixed with 4% PFA (Sigma-Aldrich, Cat#158127-100 G) for 20 min and washed again with PBS supplemented with 50 mM ammonium chloride. Cells were then incubated in the dark with anti-Mouse Cy3 (Abcam, Cat#ab97035; RRID: AB_10680176) in a 1:7.5 primary:secondary ratio in buffer for 1 h. The cells were then washed with PBS, stained with DAPI (0.1 μg/ml) and mounted using Immu-Mount (Thermo Fisher Scientific, Cat10622689). Imaging was performed using a Nikon ECLIPSE TE2000-U inverted fluorescence microscope. Quantification was performed using ImageJ^113^ using the with the colocalisation tool and a macro that can be made available upon request. This colocalisation analysis compares the pixels in both channels (green (transfected cells) and red (anti-His/Cy3 clustered antibodies)) and determines which pixels have a signal in both channels. A ratio of 10% between red and green channels was used. As the cells are non-permeabilised, the signal coming from the red channel (Cy3) can only come from the soluble His-tagged proteins on the cell surface, if present. The co-localisation area of green pixels (transfected cells) with red pixels (cell-bound protein) was normalised against the area of transfected cells and converted into a percentage. For the statistical analysis a one-way ANOVA test was used, with a Tukey’s post-hoc test in GraphPad Prism (version 10 for MacOS, GraphPad Software, San Diego, California, USA, https://www.graphpad.com/). Significance was determined when *p* < 0.05.

### Surface biotinylation of surface-expressed proteins

HEK293T cells were grown on T75 flasks until 90% confluent. Then, cells were transfected using 780μl of optiMEM, 70.2 μl of PEI, and 23.4 μg of DNA (empty pCAGIG, Ten4 WT, Ten4 nT or Ten4 nL). To assess protein expression on K562, cells were electroporated as explained above (see cell-cell aggregation assay). Both cell lines were incubated overnight ( ~ 16 h) at 37 °C. To perform the surface biotinylation and extraction of surface-bound proteins, we used the Pierce™ Cell Surface Biotinylation and Isolation Kit (Cat#A44390) and followed manufacturer’s guidelines. For HEK293T cells, we removed the media and washed cells with 10 mL of ambient PBS per T75. We dissolved the contents of one vial of Sulfo-NHS-SS-Biotin in 24 mL of ambient PBS. We added 5 mL of the biotin solution to one T75 and incubated the T75s for 10 min at RT. After the incubation, we removed labelling solution and washed cells twice with 10 mL ice-cold TBS. removing the TBS after each wash. We scrapped the cells into 10 mL ice-cold TBS and put them on a 50 mL conical tube on ice. We rinsed the scraped T75S with 5 mL each of TBS and added the rinse volume to transferred cells. We then centrifuged cells at 500 × *g* for 3 min at 4 °C and discarded the supernatant.

For K562 cells, we grew the cells in suspension to ~4 × 10^5^ cells/ml having a total of 15 ml. We harvested and centrifuged cells at 300 × *g* for 3 min and discarded the media. We washed the cells with 10 ml of PBS, centrifuged them at 300 × *g* for 3 min and discarded the wash. We added 5 ml of the biotin solution (prepared as explained above) to the tube and resuspended the cells. These were incubated for 10 min at RT. We then centrifuged the cells at 300 × *g* for 3 min and removed label solution. Cells were washed with 10 ml ice-cold TBS two times with a centrifugation step in between at 300 × *g* for 3 min discarding the supernatant. For both cell lines, we added 50 μl of 10× cOmplete^™^, Mini, EDTA-free Protease Inhibitor Cocktail (Sigma Aldrich, Cat# 11836170001) to 500 μl of Lysis Buffer and added 250 μl to the cell pellets. We resuspended the cells 20 times and transferred the cells to Eppendorf tubes. These were vortexed for 5 s before and after an incubation on ice for 30 min. The cell lysate was centrifuged at 15,000 × *g* for 5 min at 4 °C. The supernatant was incubated with 125 μl of NeutrAvidin^TM^ Agarose slurry for 30 min at RT on columns. After incubation, the column was centrifuged for 1 min at 1000 × *g*. The columns were then washed 4 times with the wash buffer. We mixed 225 μL Elution Buffer and 25 μL DTT stock solution and added 100 μL of the buffer to the resin. This reaction was incubated for 30 min at RT. The supernatant was then collected after centrifuging for 2 min at 1000 × *g*. The eluate was then analysed using SDS-PAGE and Western Blot as explained above (see shRNA validation in vitro). We used mouse anti-HA (Sigma-Aldrich, Cat#H3663) and anti-mouse HRP (Sigma-Aldrich, Cat#A0168) to detect Ten4 (WT/nT/nL) protein levels. As a loading control, we used rabbit anti-Transferrin Receptor (Abcam, Cat#ab214039) and anti-rabbit HRP (Abcam, Cat#ab6721) to detect the levels of extracted Transferrin Receptor.

### Liquid chromatography-mass spectrometry (LC-MS) and mass spectrometry analysis

For sample preparation, 17.5 µg protein present in 20 mM Tris, 300 mM NaCl buffer, were treated with 500 U of PNGaseF (NEB P0705S) for 1 h at 37 °C. Subsequently, the samples were denatured in 4 M Urea/0.1 M ammonium bicarbonate buffer pH 8.0, followed by 30 min incubation with 10 mM TCEP at RT and 30 min incubation with 50 mM 2-chloroacetamide at RT in the dark. Samples were diluted 4 times in 0.1 M ammonium bicarbonate buffer and digested with protease chymotrypsin (1 µg enzyme: 40 µg protein), 10 mM calcium chloride, overnight at RT. Digestion was stopped with the addition of formic acid (5%). Digested peptides were centrifuged for 30 min at 15,150 × *g* at 4 °C. The supernatant was loaded onto C18 stage tips, pre-activated with 100% acetonitrile and 0.1% formic acid. Centrifugation was carried out at 1500 × *g* at RT. Next digested peptides were loaded onto the C18 column. Subsequently, peptides were washed with 0.1% formic acid and eluted in 50% acetonitrile/0.1% formic acid. Eluted peptides were dried in a speed-vac before resuspension into 5% acetonitrile/5% formic acid for LC-MS/MS analysis. Peptides were separated by nano liquid chromatography (Thermo Scientific Easy-nLC 1000), coupled in line a Q Exactive mass spectrometer equipped with an Easy-Spray source (Thermo Fisher Scientific). Peptides were trapped onto a C18 PepMac100 precolumn (300 µm i.d.x 5 mm, 100 Å, Thermo Fisher Scientific) using Solvent A (0.1% Formic acid, HPLC grade water). The peptides were further separated onto an Easy-Spray RSLC C18 column (75 µm i.d., 50 cm length, Thermo Fischer Scientific) using a 30-min linear gradient (15% to 38% solvent B (0.1% formic acid in acetonitrile)) at a flow rate 200 nl/min. The raw data were acquired on the mass spectrometer in a data-dependent acquisition mode (DDA). Full-scan MS spectra were acquired in the Orbitrap (Scan range 350–1500 m/z, resolution 70,000; AGC target, 3e6, maximum injection time, 50 ms). The 10 most intense peaks were selected for higher-energy collision dissociation (HCD) fragmentation at 30% of normalized collision energy. HCD spectra were acquired in the Orbitrap at resolution 17,500, AGC target 5e4, maximum injection time 120 ms with fixed mass at 180 m/z. Charge exclusion was selected for unassigned and 1+ ions. The dynamic exclusion was set to 5 s. For data processing, tandem mass (MS/MS) spectra were searched using PEAKS X software version 10.0 as follows against a protein sequence database containing 20,606 protein entries, including 20,323 *Homo Sapiens* proteins (Uniprot release from 2024-02-16), 283 common contaminants and mouse Teneurin 2 and 4 WT and variants sequences. During database searching for full chymotryptic peptides, cysteines (C) were considered to be fully carbamidomethylated (+57,02, statically added), methionine (M) to be fully oxidised (+15,99, dynamically added), all N-terminal residues to be acetylated (+42,01, dynamically added) and asparagine to be converted into aspartic acid (−0.98, dynamically added). Three missed cleavages were permitted. Peptide mass tolerance was set at 20 ppm on the precursor and 0.6 Da on the fragment ions. Data was filtered at a False Discovery Rate below 1% at the peptide level. The mass spectrometry proteomics data have been deposited to the ProteomeXchange Consortium via the PRIDE partner repository with the dataset identifier PXD058769.

### Enzyme-linked immunosorbent assays (ELISA)

NuncTM Maxisorp^TM^ 96-well plates (Thermo Scientific, Cat#735-0083) were coated with TwinStrep Teneurin2–4 or Latrophilin3 at a concentration of either 25 nM or 15 nM and left overnight at 4 °C. The plates were washed three times with PBS supplemented with Tween20 (PBST) and dried. All the wells were blocked with BlockerTM Casein in PBS (ThermoScientific, Cat#37528) for 30 min at RT. After blocking, plates were washed with PBST three times and 50 μl of the anti-Ten4 nanobodies were added at a concentration of 500 nM diluted in Casein. We used an anti-GPC3 nanobody (Nano^glue^)^42^ as a negative control. Blank wells were left in Casein in PBS. Plates were incubated for 60 min at RT and then washed 3 times with PBST. 50 μl of the primary antibody solution was then added to the wells and left for 1 h at RT. For the nanobody-containing wells, mouse anti-His antibody (Thermo Fisher Scientific; Cat#372900; RRID: AB_2533309) at a dilution of 1:1000 was used, but to create a standard curve, we used a titration of mouse anti-Strep antibody (IBA, Cat#2-1507-001) starting from 5 μg/ml was added to wells that were coated with Ten4 but were not added nanobody. The plates were all washed with PBST and all wells were added 50 μl of a solution containing anti-mouse IgG alkaline phosphatase antibody (Sigma-Aldrich, Cat#A1418-.25 ML, 1:10,000 in casein) and incubated for 30 min at RT in the dark. Plates were washed with PBST and PBS and revealed with 100 μl of a buffer containing 1× diethanolamine buffer (Fischer, Cat#34064) and 20 mg of 4-nitrophenylphosphate tablet (Sigma, Cat#N2765-50TAB) diluted to a concentration of 1 mg/ml. Plates were then read every one minute noting the 405 nm absorbance until one sample reached an OD of 1.3. ELISA readings were standardised by calculating the linear fit for the Streptavidin standards and applying the linear equation to the nanobody readings to calculate the normalised response. We reported the results as arbitrary units (AU). For the Kd calculation, a titration of anti-Ten4 nanobody was prepared starting from 10,000 nM and readings were produced following the same protocol. After standardisation of the readings, the Kd and fit were calculated using an equation described before^115^.

### Stimulated emission depletion (STED) microscopy

A shRNA control (miR30 control GFP+) slice was immunostained following a similar protocol described above. The brain slice (thickness 75 μm) was incubated for 2 h at RT in PBS with 0.5% Triton ×-100 (Sigma-Aldrich, Cat#X100-100ml) and then rinsed with PBS. The slice was incubated overnight at 4 °C in NanoTen4 and anti-BLBP antibody (Millipore-Merck, Cat#Abn14), both diluted 1:50 in PBS with 0.3% Triton ×-100, 0.2% BSA, 0.2% Gly, 0.2% Lys and 5% donkey serum. The slice was washed three times with PBS and further incubated three hours at 4° with a dilution of 1:250 of anti-rabbit AlexaFluor 594 secondary antibody (Fisher Scientific, Cat#A21207). The slice was washed in PBS for 30 min and mounted using Mowiol (pH 8.5, no propyl gallate) and a #1.5H high precision coverslip (Electron Microscopy Sciences, Cat#71861-054-C).

For super-resolution STED imaging, the sample was acquired with a STELLARIS 5 TauSTED Xtend confocal microscope system (Leica Microsystems) using a HC PL APO 100×/1.40 OIL STED WHITE oil objective. NanoTen4 and anti-BLBP were imaged in 2D TauSTED mode and GFP signal in conventional confocal mode. Z-stacks of ~10–15 μm hight were acquired with 500 nm z-steps and a pixel size set to 21–23 nm. A white light laser set to 100% power was used for excitation and a 775 nm solid state laser was used for depletion of both STED channels. Channel shifts between STED and confocal channels were determined from acquisitions of 100 nm TetraSpek beads (Thermo Fisher) and corrected using ImageJ^113^ (12 pixels to the left and 8 pixels up).

The STED images were analysed using two custom macros on ImageJ^113^ (available upon request). ROIs were first generated to select the RGC fibres using the BLBP STED images. Then, an area around the fibre at a maximum distance of 1.5 μm from the centre of the fibre was selected in an unbiased manner, independent of the NanoTen4 staining. NanoTen4 STED images were first denoised by applying a median (0) filter and intensity-normalised before converting to a binary image applying an arbitrary threshold of 1000. Next, the area of NanoTen4-positive pixels within the ROIs generated in the previous step for all stacks and images was determined and the enrichment of NanoTen4 for each individual RGC fibre calculated relative to the surrounding area for each image. Averaged values for each image in the stacks (individual image) were reported, and the ratios between the enrichment of RGC fibre and the surrounding area were determined. For the classification of stacks into high/mid/low NanoTen4 stained areas, we calculated the thresholds for each level by using descriptive statistics and using the values for the 25 and 75 percentiles for the enrichment of NanoTen4 on RGC fibres on the CP as thresholds.

### Basescope^TM^ and sequencing validation of CRISPR-introduced nT and nL mutations

BaseScope^TM^ assays were performed following guidelines (BaseScope™ Reagent Kit- v2 RED) provided by the supplier (Advanced Cell Diagnostics, Newark, CA). In brief, electroporated CRISPR brains were fixed in 4% PFA overnight and cut using a cryostat to a 10 μm thickness. 10 μm Cryo-sections were pretreated with hydrogen peroxide, target retrieval and protease III. Then BaseScope^TM^ probes (the three probes were all purchased from Advanced Cell Diagnostics and sequences are available in the Key Resources table) were applied for 2 h at 40 °C in a HybEZ oven follow by the amplificator reagents following the protocol indications. Finally, slides were incubated with Fast Red for 10 min at RT in the dark. Then, nuclei were counterstained with DAPI, and sections were mounted with Mowiol. Images were acquired using Thunder microscope (Leica) with a 20× objective and using 2 cameras, the colour for Basescope detection and the fluorescent camera for GFP/DAPI detection. Images were processed with ImageJ^113^ and Cell profiler software^114^.

For sequencing, electroporated CRISPR brains were dissected in cold HBSS using a Leica MZ10 Stereomicroscope equipped with a fluorescent lamp. The electroporated region from each brain was collected into an Eppendorf tube, centrifuged, and the tissue was stored at −80 °C until further processing. DNA was extracted by boiling the samples in 50 µl of 50 mM NaOH at 95 °C for 5 min, followed by neutralization with 5 µl of 1.5 M Tris-HCl (pH 8.8). PCR was used to amplify the targeted region for the Ten4 TT mutation (Exon 30, position 96892998) using the following primers: Forward: GATCTACGATGACCATCGCA and Reverse: TGCAAAGATCCGGGATGTG. The PCR reaction was performed using Invitrogen™ Platinum™ II Hot-Start PCR Master Mix (2×), and the amplicons (220 bp for nT and 157 bp for nL) were purified using the QIAquick PCR Purification Kit. The quality assessment, sequencing, and raw data control for each sample were performed using the INVIEW CRISPR Check (150–270 bp) service from Eurofins (https://eurofinsgenomics.eu/en/next-generation-sequencing/ngs-built-for-you/inview-crispr-check/). The average quality score ranged from 88.59% to 97.20%, and the total number of read pairs was approximately 5 million per sample. We used R, along with the ShortRead and ggplot2 libraries, to identify the correctly CRISPR-edited transcripts: ACCTGACCGGCGTGAACGTGACA for the nT mutation and GTGACAGTGTTCGGCAGAGAC for the nL mutation. The oligonucleotides’ sequences are available in a Supplementary Data file.

### RNA In situ hybridization (ISH) and Nanobody Immunohistochemistry in brain tissue

Embryonic brains were fixed in 4% PFA overnight and cut to 10 μm thickness as explained above. 10 μm Cryo-sections were pre-treated using the RNAscope Universal Pretreatment Kit (Advanced Cell Diagnostics) as indicated by the manufacturers. ISH were performed using the RNAscope Fluorescent Multiplex Reagent Kit as it was performed for cultured neurons (see above). The target gene (Ten4) was detected with the probe: Mm-Tenm4-C1 (RNAscope; Cat#555491). Following ISH, sections were immunostained O/N using the NanoTen4 conjugated to AlexaFluor633 (1:50) (generated and validated in this manuscript). NanoTen4 was diluted in 2% BSA, 0.3% Triton ×-100, PBS. Nuclei were counterstained with DAPI, and sections were mounted with Mowiol. Images were acquired using a Zeiss LSM880 confocal laser scanning microscope using a 20×, objective and 2 Airy disk pinhole, and analysed with ImageJ^113^ software. The oligonucleotides’ sequences are available in a Supplementary Data file.

### Model building and refinement

All model building was performed in Phenix^116^ (version 1.20.1-4487) using the dock in map and real space refinement functions. Refinement in Phenix (using default constraints) and in COOT^117^ was performed iteratively. Initial model generation was performed using PHYRE2^118^ inputting the Ten2 full ectodomain sequence (see vectors and cloning) for each splice site variant. Regions of this initial model not present in the cryo-EM density were trimmed after initial docking in ChimeraX^119^. All figure panels showing structures or maps were made using ChimeraX^119^. Cryo-EM density maps shown in Fig. 1 and Supplementary Fig. 1, and also the density maps used for model building, were sharpened with deepEMhancer^120^ (using the HighRes model). Model refinement statistics were reported with Phenix comprehensive validation using Molprobity ^121^ (see Table S2). The difference map between the Ten2 A1B1 and Ten2 A0B0 density maps was generated using the Surface colour volume tool in ChimeraX. The Ten2 A1B1 map was coloured by the volume data values of the Ten2 A0B0 map. The maximum value (0.25, depicted as blue) corresponds to voxels that present the same values in both maps. The minimum value (0, depicted as red) corresponds to voxels that are present in the Ten2 A1B1 map but not in the Ten2 A0B0 map. Note that 0.25 is the contour level used for all maps.

### Analysis of published single cell RNASeq datasets

Single-cell RNAseq data for cortex samples (Fig. 4 and Supplementary Fig. 5) were obtained from the published NCBI Gene Expression Omnibus with accession number GSE153164^44^. For the single-cell RNA-seq data for dissociated cortical neurons (Fig. 7 and Supplementary Fig. 8), we used a published NCBI Gene Expression Omnibus with accession number GSE271794^122^. We used the same UMAP coordinates and metadata information with the cluster categorization provided by the authors. We used the R package Seurat (v.4.0.1) to perform all the analysis.

### Nanobody discovery

We generated anti-Ten4 nanobodies by immunising one llama with the purified ternary complex of the mouse Teneurin4 ectodomain, the mouse Latrophilin1 Lec-Olf domains, and the mouse FLRT1 (mTen4-mFLRT1-mLphn/ADGRL1) as previously described ^122^. Briefly, a llama was immunized 6 times with purified ternary complex. Peripheral blood lymphocytes were collected after the final boost, RNA was purified, and cDNA created which served as template to generate a nanobody phage library in the pMESy4 vector encoding a His^6^-tag and EPEA-tag at the C-terminal end of the protein. After two rounds of phage display selection on the ternary complex or on mTen4 only, and screening by ELISA using established protocols^122^. 13 different families, classified according to the sequences of the third complementarity determining region (CDR3), were discovered. These families were further screened using an optimised ELISA protocol (described above under Enzyme Linked Immunosorbent Assay (ELISA)) and one anti-Ten4 nanobody (NanoTen4, http://www.NanoSaurus.org entry SD-AW7W) was selected as the best binder, which was used throughout this manuscript. The CDR loop sequences of NanoTen4 are GRTISNAV (CDR1), VSYSGRFT (CDR2), and AAKGSWSAIPNPSDYDY (CDR3). The structural model of Anti-Ten4 nanobody (NanoTen4) shown in Supplementary Fig. 4A, B was generated using Alphafold3 ^123^.

### Quantification and statistical analysis

Statistical analyses were performed using GraphPad Prism version 10, employing a one/two-tailed unpaired Student’s *t* test, and one-way ANOVA test with Tukey’s post hoc analysis when comparing multiple groups. For the analysis of the fraction of cells and the cumulative frequency distribution, we used chi-square contingency analysis and the Kolmogorov–Smirnov test, respectively. *p* values represent ∗*p* ≤ 0.05, ∗∗*p* ≤ 0.01, ∗∗∗*p* ≤ 0.001 and ∗∗∗∗*p* ≤ 0.0001. All statistical tests and *p-values* for each panel are explained in the figure legends. All data are presented as the mean ± SEM, whisker plots, violin plots, bar plots or dot plots. All sample sizes and definitions are provided in the figure legends.

### Reporting summary

Further information on research design is available in the Nature Portfolio Reporting Summary linked to this article.

## Supplementary information

Supplementary Information

Description of Additional Supplementary Files

Supplementary Data 1

Reporting Summary

Transparent Peer Review file

## Source data

Source Data

## Data Availability

The electron density maps for the mouse Ten2 dimers have been deposited in the Electron Microscopy Data Bank under the accession codes: EMD-51022 (A0B0) (https://www.ebi.ac.uk/emdb/EMD-51022), EMD-50975 (A1B1) (https://www.ebi.ac.uk/emdb/EMD-50975), EMD-50976 (A1BO) (https://www.ebi.ac.uk/emdb/EMD-50976), and EMD-51021 (A0B1) (https://www.ebi.ac.uk/emdb/EMD-51021). The atomic coordinates and models resulting from the structural analysis of the aforementioned maps have been deposited on the Protein Data Bank under the following accession codes, respectively: 9G42 (A0B0) (10.2210/pdb9G42/pdb), 9G2F (A1B1) (10.2210/pdb9G2F/pdb), 9G2H (A1B0) (10.2210/pdb9G2H/pdb), and 9G41 (A0B1) (10.2210/pdb9G41/pdb). The mass spectrometry data of the Teneurin mutants can be found in ProteomeXchange under the accession code: PXD058769. The tissue mass spectrometry data of the different cortical layers can be found in the PRIDE repository under the accession code: PXD075073 (https://www.ebi.ac.uk/pride/archive/projects/PDX075073). The Teneurin3 and 4 dimers shown in Supplementary Fig. 1 are available in the PDB: 8R50 (10.2210/pdb8R50/pdb) and 7BAM (10.2210/pdb7BAM/pdb). Single-cell RNA-seq data is available in NCBI GEO: GSE153164 and GSE271794. Molecular dynamics trajectories are available upon request. Unedited Western blots are shown in the Supplementary Information File. Source data are provided with this paper.

## Materials availability

This study generated a new anti-Ten4 nanobody (NanoTen4, http://www.NanoSaurus.org entry SD-AW7W), which can be made available upon request to the VIB (Els Pardon/Jan Steyaert) and the lead contact (Elena Seiradake). CDR loop sequences are available in the Nanobody discovery methods’ section and Supplementary Fig. 4A, B.

## Code availability

The scripts and macros used for quantification of the stripe assays or cell binding assays can be made available upon request to the lead contacts Daniel del Toro (danieldeltoro@ub.edu) and Elena Seiradake (elena.seiradake@bioch.ox.ac.uk).
