# Supplementary material for: Structurally exclusive Teneurin complexes orchestrate divergent programs in early cortical development
Source: Nat Commun. 2026 Apr 16;17:5292. doi: 10.1038/s41467-026-71619-1 (PMC13269823; doi:10.1038/s41467-026-71619-1)

Supplementary Fig. 1 (associated with Fig. 1):

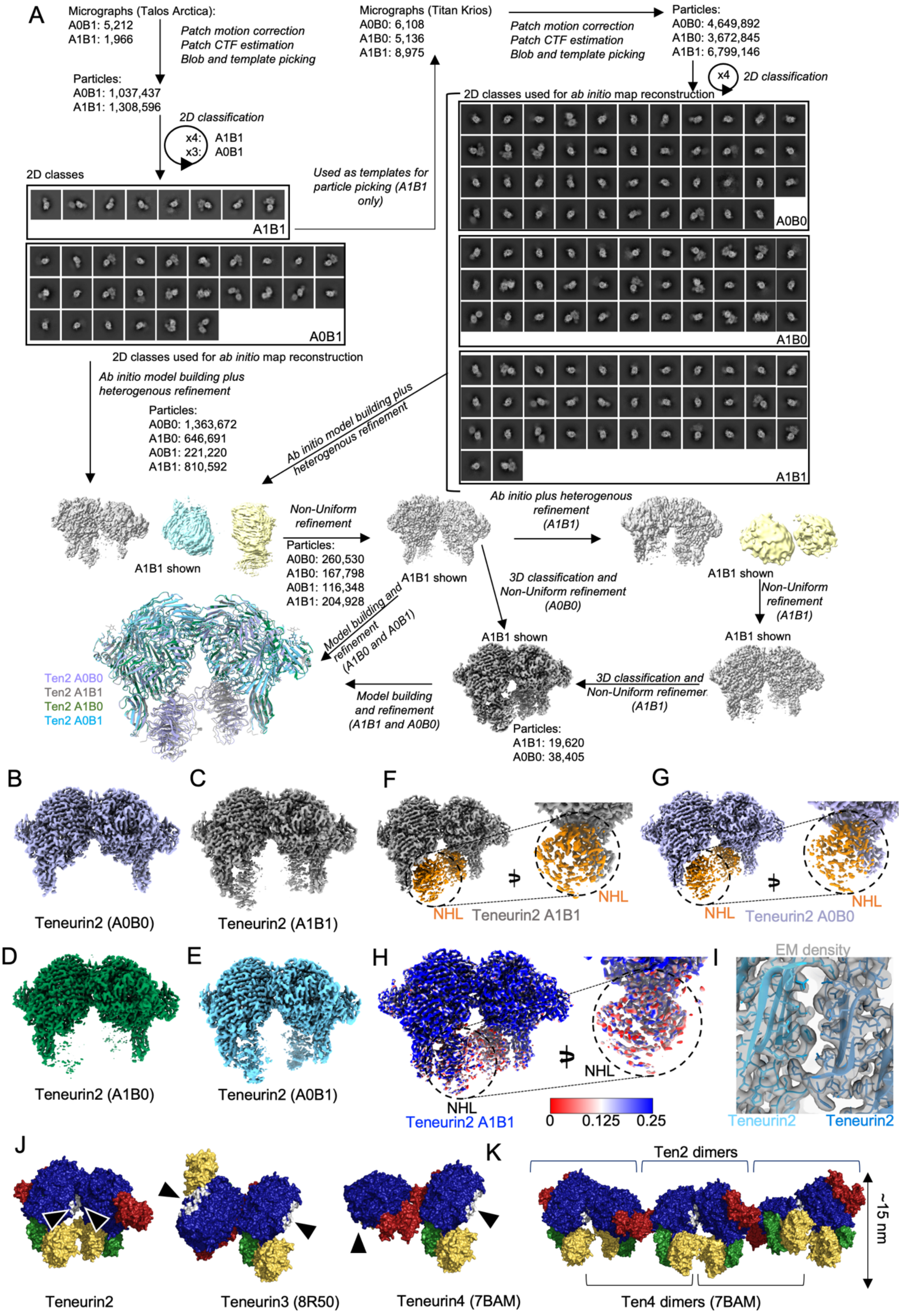

Supplementary Fig 1. Single-particle Cryo-EM data analysis. A: Cryo-EM processing strategy and workflow in cryoSPARC. B-E: Resulting cryoEM maps for the four murine Ten2 homodimers. F,G: Resulting dimer maps for isoforms A1B1 (panel F) and A0B0 (panel G) of Ten2, derived using ~15% of the particles after 3D classification and non-uniform refinement (see panel A). The NHL domain (corresponds to orange areas in the map) is better defined after this processing step. H: The map of the Ten2 A1B1 isoform is depicted (equivalent to that shown in panel F), but coloured according to how it differs from the map calculated for the A0B0 isoform (panel G). We used the surface colour volume tool and the colour by volume data values option in ChimeraX [116] to do this. The maximum value (0.25, depicted as blue), corresponds to voxels that present the same values in both maps. The minimum value (0, depicted as red), corresponds to voxels that are present in the Ten2 A1B1 map but not in the Ten2 A0B0 map. 0.25 is the contour level used for all maps. Most differences are found in the region corresponding to the NHL domain, which is less defined in the map for the A0B0 isoform. I: Zoomed-in view of the Teneurin2 dimer interface. The EM density map (grey) and model (shades of blue) are show. J: Comparison of the Ten2 dimer structure and previously published mammalian Teneurin dimers, which dimerise via different surfaces. Individual domains are coloured as follows: YD-shell: navy. NHL domain: yellow. FN-plug domain: green. C-terminal ABD and Tox-GHH domains: red. The Ten2-Ten2 interaction surface is coloured in white and indicated by arrow heads. The equivalent homologous surfaces on Ten3 and Ten4 are indicated in the relevant structures. K: Alternating the interfaces of the Ten2 and Ten4 dimer structures (PDB 7BAM [9]) produce arrays. Ten2 structures are shown as surface views, coloured as in panel J.

Supplementary Fig. 2 (associated with Fig. 2):

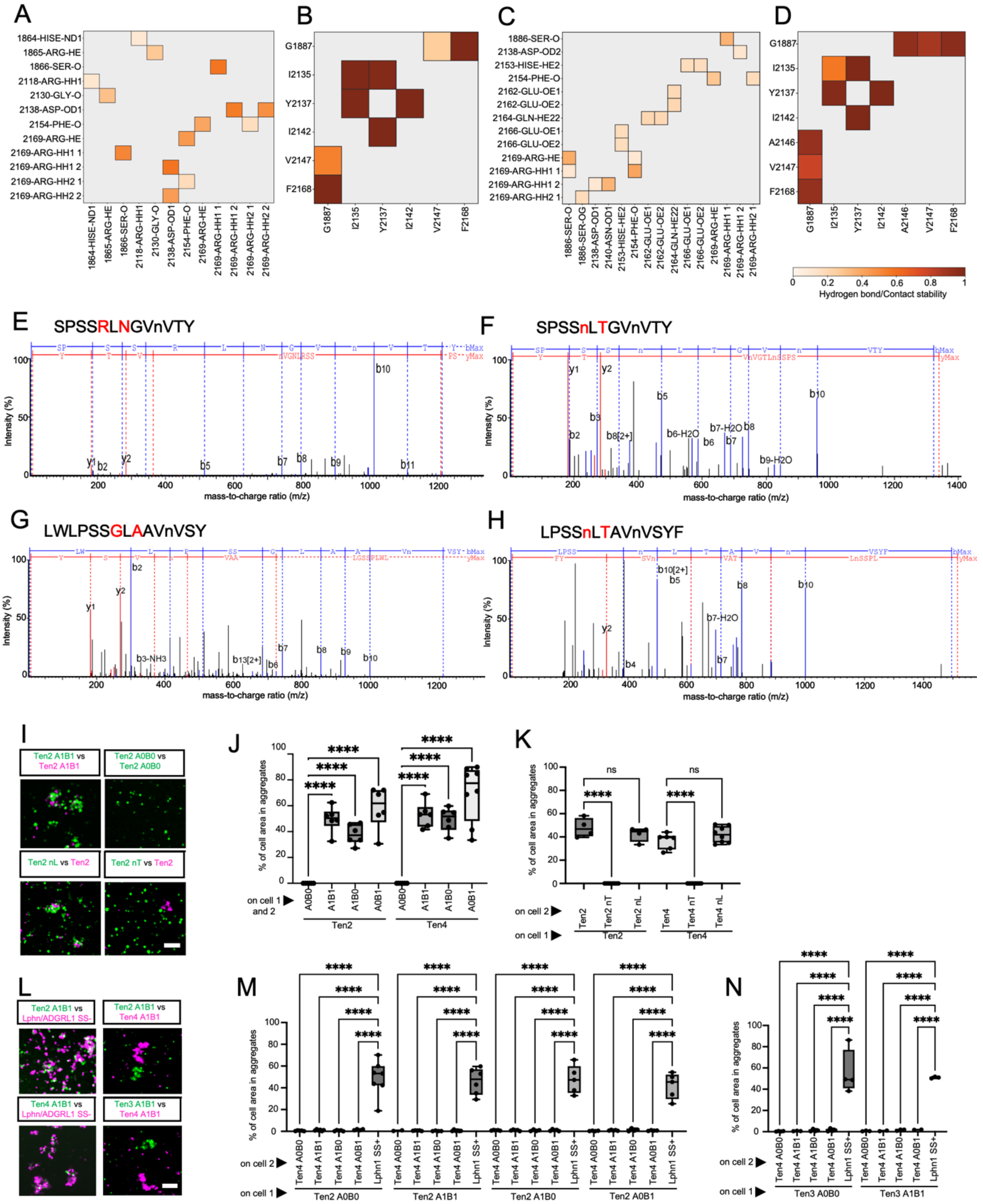

Supplementary Fig. 2: Ten2 interface analysis, mutant validation and cell aggregation. A-D: Summary of key residues that contribute to hydrophilic and hydrophobic interactions (red = most stable, white = least stable) in the Ten2-Ten2 interface, during MD simulation of the Ten2 dimer structure. Results are plotted after backbone constrained simulation (panels A, B) and after unconstrained simulation (panels C,D). E-H: We used liquid chromatography-

mass spectrometry (LC-MS) to reveal N-linked glycosylation sites in chymotrypsin-derived Ten2 and Ten4 ectodomain peptides. Chymotrypsin cuts after F, W, Y. In the nT mutants we we introduced an N-linked glycan at positions G1887N (Ten2) and R1881N (Ten4) by mutating these sites to asparagine (N), followed by a non-proline residue, followed by a threonine (T). The mass spectrometry analysis confirms this. Results are shown for Ten2 WT (panel E), Ten2 nT (panel F), Ten4 WT (panel G) and Ten4 nT (panel H), focusing on the peptide that is mutated in the nT mutants. The mutated residues are highlighted in red. A lower-case n (instead of upper-case N) indicates that the site was indeed glycosylated. I-K: Cell-based aggregation results showing different levels of *'in trans'* binding for different Teneurin constructs. N>4 replicates. 6 pictures per replicate. L-N: Cell-based aggregation results showing no aggregation between the different Teneurin4 homologues Ten2, Ten3 and Ten4. N>2 replicates. 6 pictures per replicate. Representative images are shown in L. n.s. = not significant. ****p < 0.0001. One-way ANOVA test with Tukey's post hoc analysis (J,K,M,N). Scale bars represent 100 μm (I,L). Source data are provided as a Source Data file.

Supplementary Fig. 3 (associated with Fig. 2):

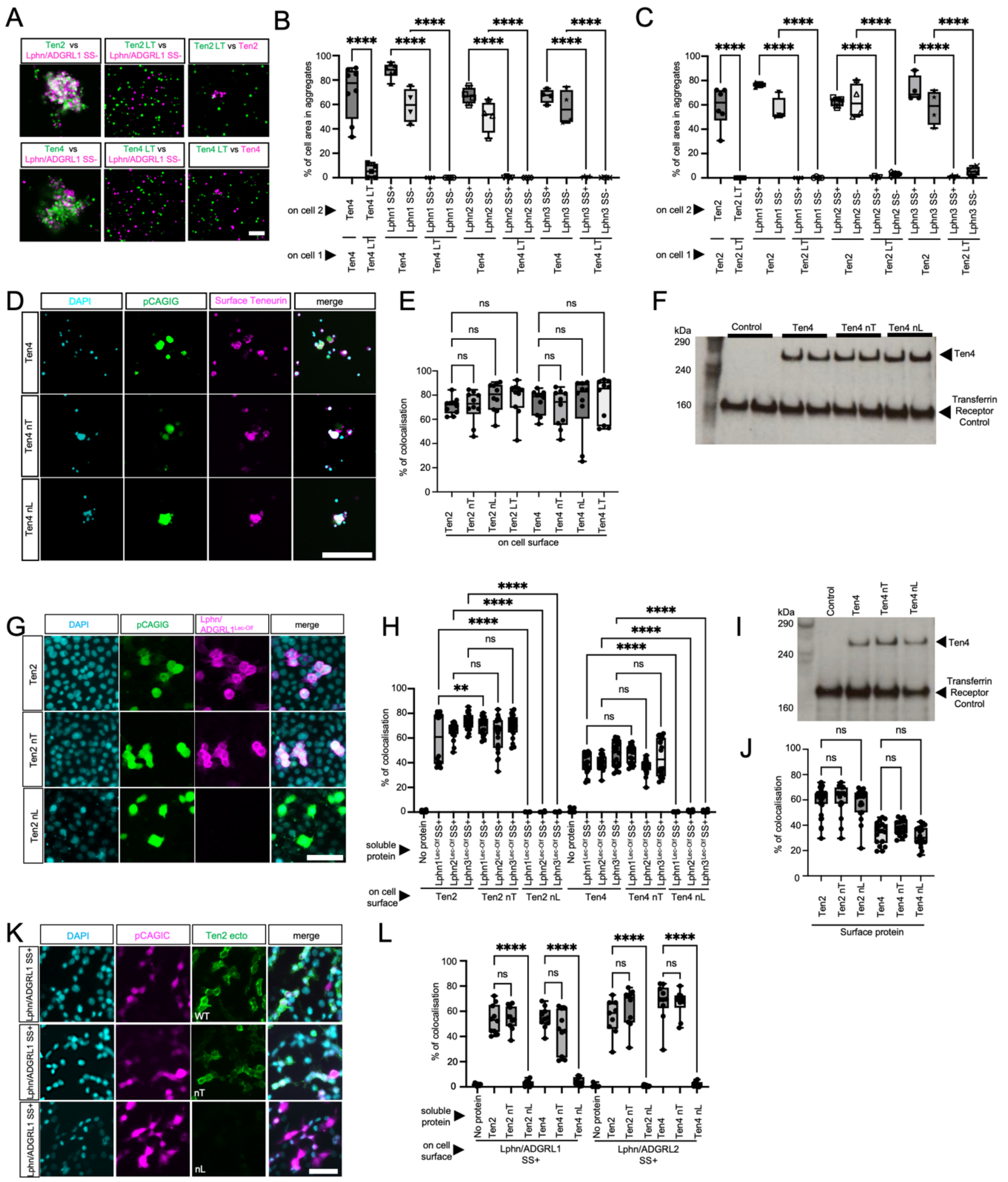

Supplementary Fig. 3. Cell aggregation and cell-based binding studies. A-C: Cell-cell aggregation results and representative images showing the Teneurin LT mutant [3] binding capabilities. N=4 replicates. 6 pictures per replicate. D: Representative images of a cell-surface staining experiment to assess protein expression levels at the cell surface of non-permeabilised K562 cells. E: Quantification of the surface expression levels of Ten2 and Ten4 WT, LT, nT and nL in K562 cells. Immunostaining against the extracellular HA-tag was

used to assess surface presentation of these constructs. N=2 replicates. 10 pictures per replicate. F: Western blot results after the cell surface biotinylation and extraction of all membrane-bound proteins on K562 cells electroporated with either empty pCAGIG, Ten4 WT, Ten4 nT or Ten4 nL with an HA-antibody. The level of Transferrin Receptor in the samples was used as a loading control. G,H: Results of a cell-binding assay in which soluble Latrophilin Lec-Olf (magenta) is added to cells expressing Ten2/4 WT, nT or nL (green). Representative images are shown. N=3 replicates. 10 pictures per replicate. I,J: Assessment of the surface expression levels of Ten2 and Ten4 WT, nT, LT and nL in HEK293T cells, using analogous methods as panels D-F. N=2 replicates. 10 pictures per replicate. K-L: Results of cell-binding experiments as shown in panel G and H but using soluble Ten2 or Ten4 ectodomains (WT, nT or nL) with Latrophilin-expressing cells (magenta). N=1 replicate. 10 pictures per replicate. Representative images are shown. n.s. = not significant. ****$p < 0.0001$. One-way ANOVA test with Tukey's post hoc analysis (B,C,E,H,J,L). Scale bars represent 100 μm (A,D,G,K). Source data are provided as a Source Data file.

Supplementary Fig. 4 (associated with Fig. 3):

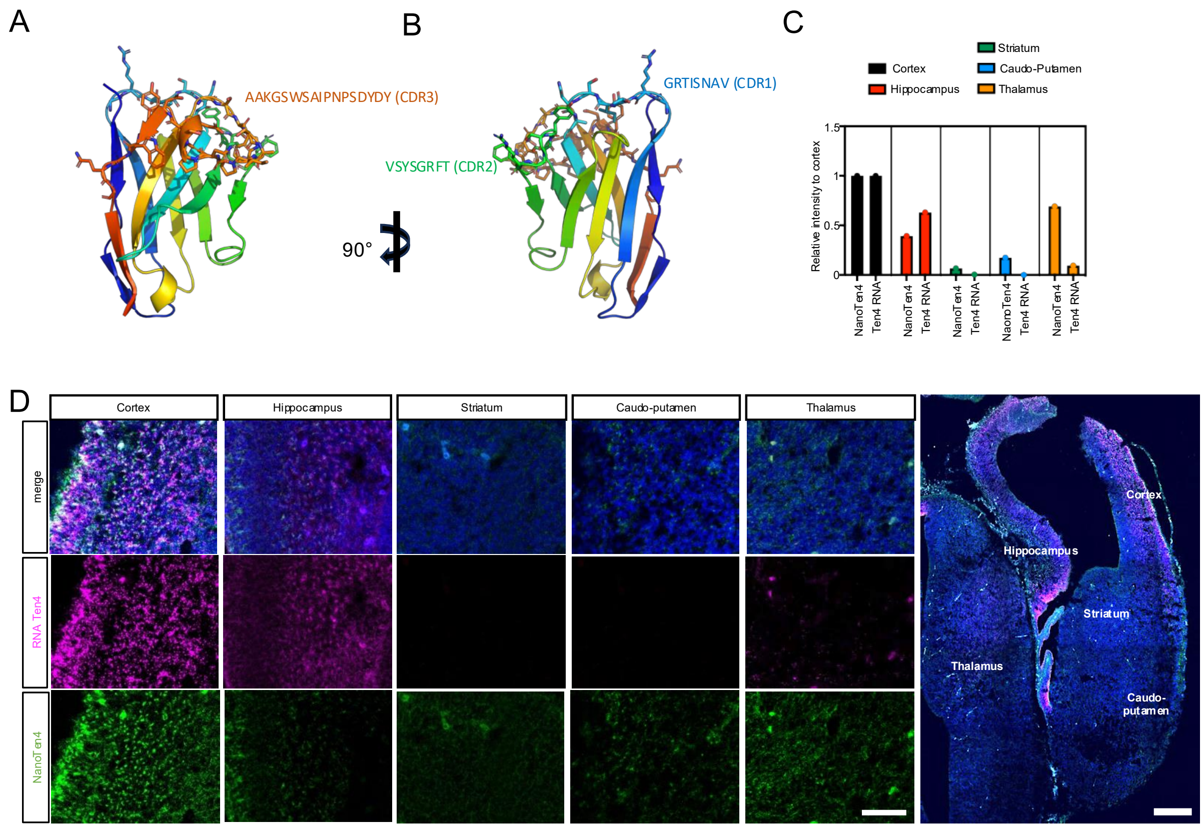

Supplementary Fig. 4. NanoTen4 and Ten4 *in situ* hybridisation analysis. A,B: Alphafold3[124] structural model of anti-Ten4 nanobody NanoTen4 (http://www.NanoSaurus.org entry SD-AW7W) with the CDR loop sequences highlighted and shown as sticks. The rest of the model is shown as a cartoon. C: Quantification of experiment shown in panel D. The NanoTen4 labelling and the presence of Ten4 mRNA largely correlate, suggesting that NanoTen4 labels Ten4 protein *in situ*. D: Different areas of an E15.5 murine brain slice, labelled with NanoTen4 and for Ten4 mRNA, were imaged and labelling area was quantified. Representative images are shown. N=3-4 sections/group from 2 mice. Scale bar represents 300 µm (A) and 50 µm inset (A). Source data are provided as a Source Data file.

Supplementary Fig. 5 (associated with Fig. 4):

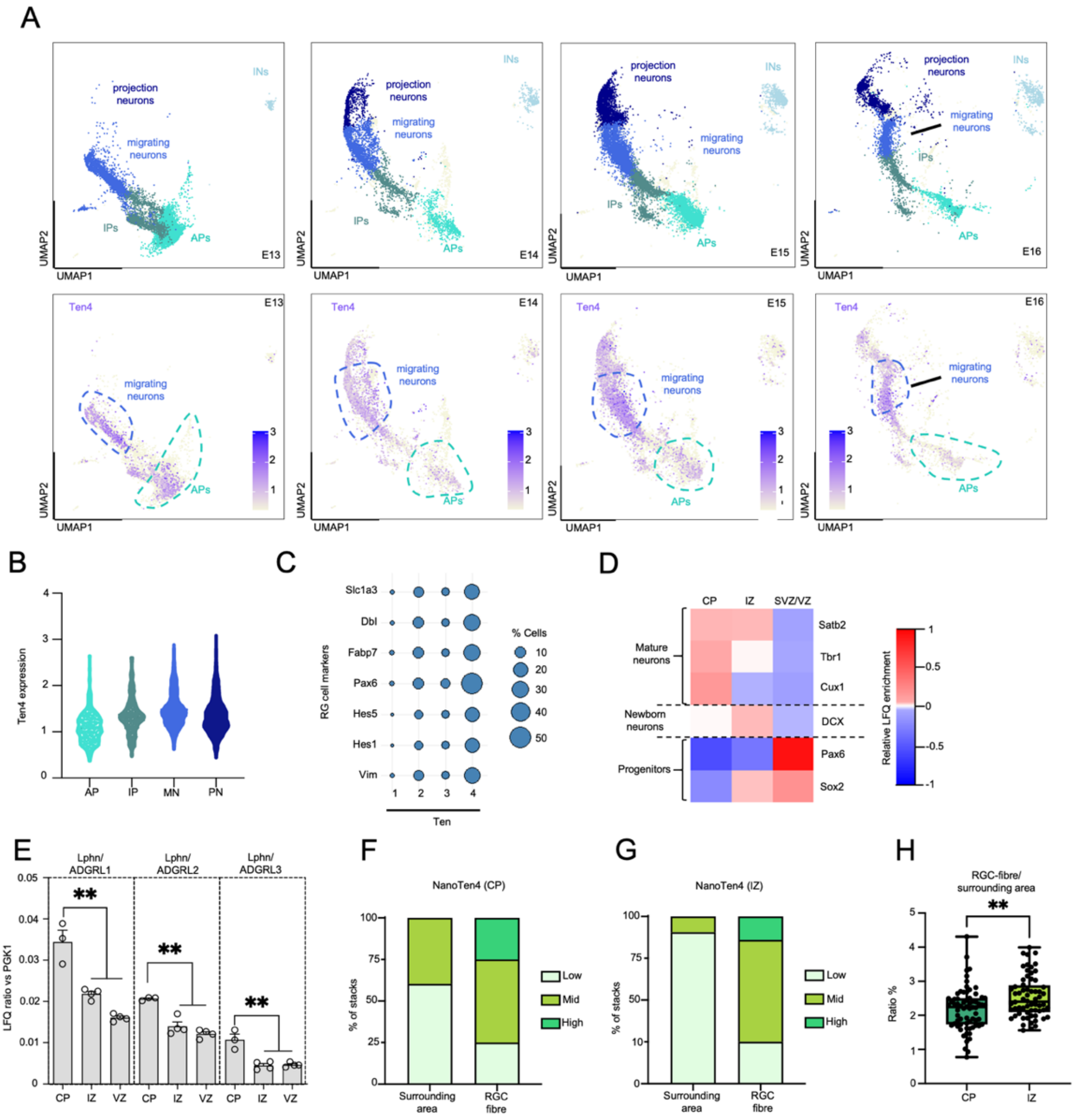

Supplementary Fig. 5. Ten4 expression analysis. A: UMAP visualizations of published single-cell data from E13, E14, E15, and E16 mouse cortex (GSE153164)[43] and corresponding plots showing Ten4 mRNA expression. B: Volcano plot showing the distribution of Ten4-positive cells in apical progenitors (AP), intermediate progenitors (IP), migrating neurons (MN), and projection neurons (PN) at E15. C: Dot plot showing that the majority of radial glial cells (RGC) express Ten4. RGC cells are defined by expressing: Slc1a3, Dbl, Fabp7, Pax6, Hes5, Hes1 and Vim. D: Analysis of neuronal and progenitor markers in mass spectrometry data confirms good separation of the cortical layers indicated. CP=cortical plate, IZ=intermediate zone, SVZ/VZ=subventricular/ventricular zones. E:

Analysis of the expression of Latrophilins (Lphns/ADGRLs) in different cortical layers using mass spectrometry. N=3-4 samples per group. F,G: We determined thresholds for low, mid, and high NanoTen4 labelling intensity using the 25 and 75 percentiles for the values of NanoTen4 staining in the areas within a 1.5 μm radius of the BLBP-positive fibres in the CP as thresholds. We plotted the data separately for areas within a 1.5 μm radius of the BLBP-positive fibres, and outside of these areas ('surrounding area') for both CP and IZ. H: Comparison of the ratio between NanoTen4 staining along radial glia cell fibres versus the surrounding area, comparing quantifications for the cortical plate versus the intermediate zone. **$p < 0.01$. One-way ANOVA test with Tukey's post hoc analysis (E). Two-way unpaired Student's t test (H). Source data are provided as a Source Data file.

Supplementary Fig. 6 (associated with Fig. 5):

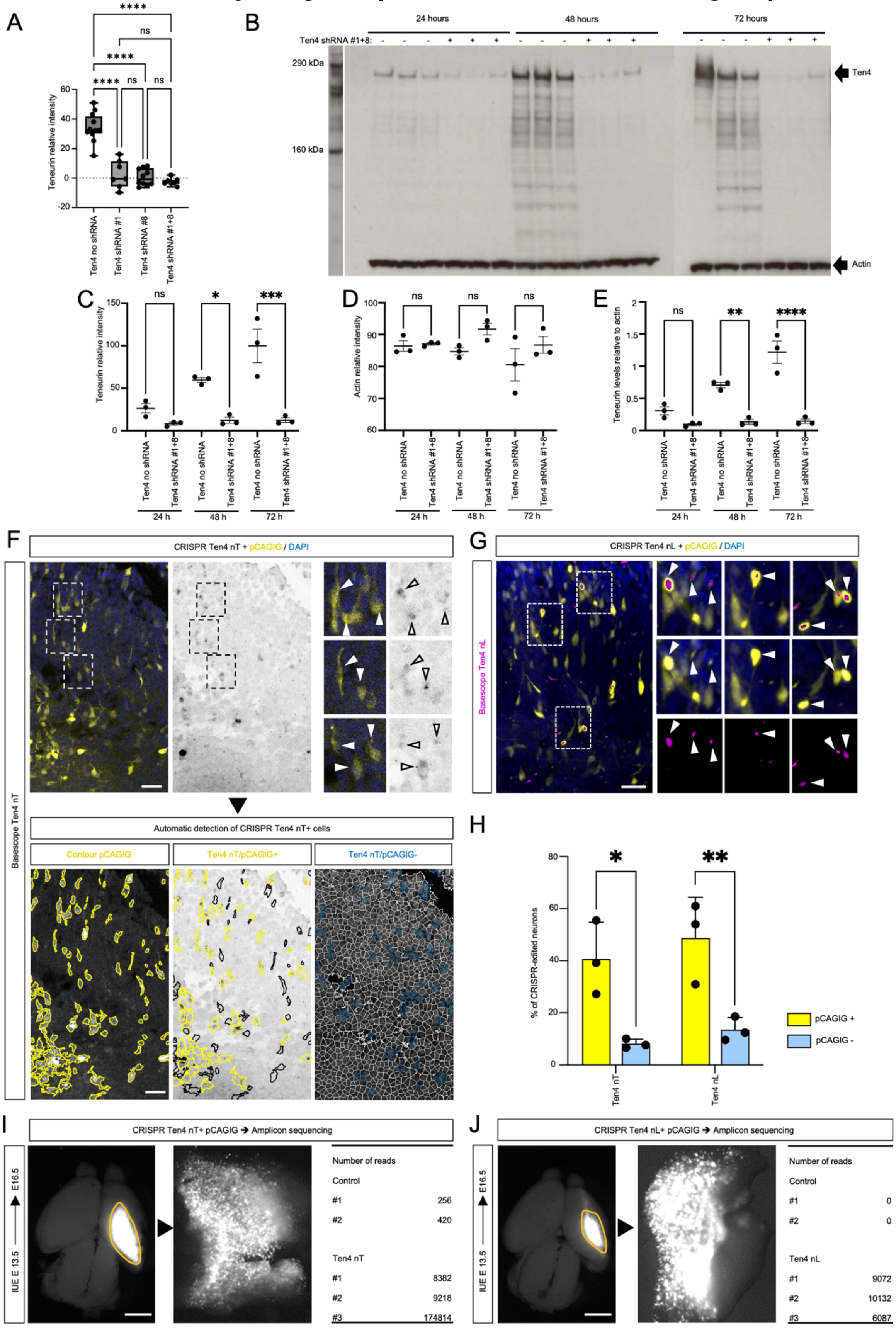

Supplementary Fig. 6. Validation of reagents used for *in vivo* analysis. A: We tested the efficacy of shRNA constructs designed to knock down Ten4 expression with Ten4-overexpressing HEK293T cells. In these experiments, HA-tagged Ten4 is co-transfected with plasmids encoding two shRNA constructs, either separately or alone (#1 and #8). Ten4 expression levels were quantified at 72h post transfection, using anti-HA western blotting. B: Based on results in panel A, we focused on using both shRNA constructs (#1 and #8), and tested their efficacy at different time points (24 hours, 48 hours, 72 hours). Actin was used as a loading control. The knock-down effect was present also after 72 hours, which is the length of an IUE experiment *in vivo*. C-D: Quantification of the intensity of protein bands (Ten4 or actin) relative to background in the Western blot shown in panel B. Box and whiskers plot. N=3 replicates. E: Quantification after normalisation, combining data in panels C+D. F-G: Basescope^{TM} labelling and automated analysis validating the successful mutation of endogenous Ten4 genes using CRISPR/Cas9 in pCAGIG (GFP) expressing cells. H: Quantification from panels F-G. N=3 embryos. I,J: Amplicon sequencing confirms the presence of CRISPR/Cas9-edited cells expressing mutant Ten4 mRNA. *p < 0.05. **p < 0.01. ***p < 0.001. ****p < 0.0001. One-way ANOVA test with Tukey's post hoc analysis (C-E). Two-tailed unpaired Student's t test (H). Scale bar represents 900 μm (I,J). Source data are provided as a Source Data file.

Supplementary Fig. 7 (associated with Fig. 6):

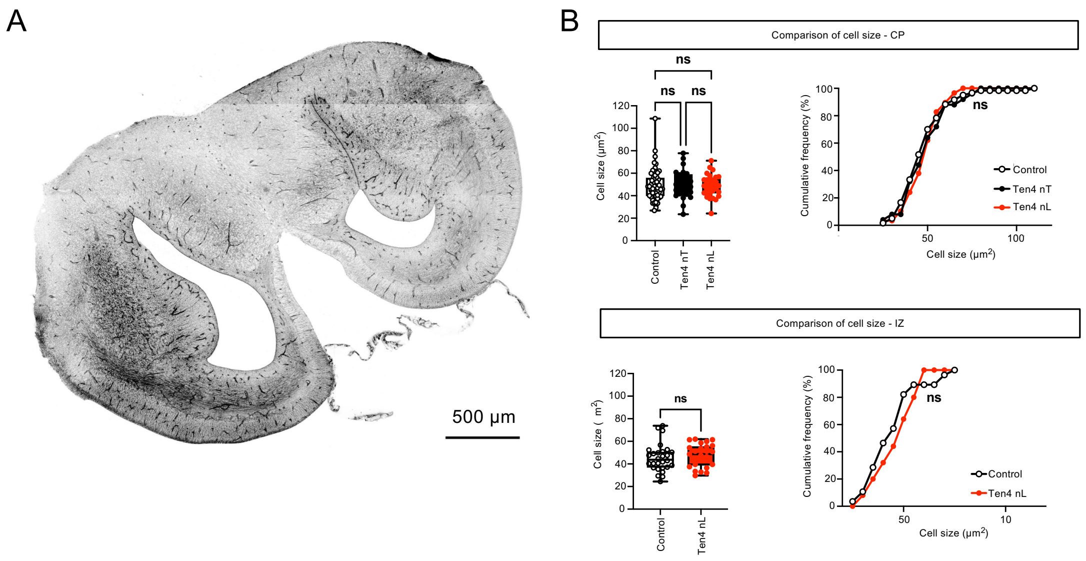

Supplementary Fig. 7. Shadow labelling suggests no change in cell size for Ten4 mutant neurons. A: Overview of a whole fixed and shadow-labelled brain slice. B: Whisker plot and cumulative frequency distribution plot for the quantification of the cell sizes of migrating neurons (mutant and control) in the cortical plate (CP) and intermediate zone (IZ). CRISPR/Cas-9 edited cells containing Ten4 mutations (nT and nL) and WT control cells were analyzed. Note that there are almost no nT mutants left in the IZ at the end of the experiment and therefore only the nL mutant was analyzed. n.s. = not significant. One-way ANOVA test with Tukey's post hoc analysis (Cell size, panel B) and two-sided Kolmogorov-Smirnov test (cumulative frequency distribution, panel B). Scale bar represents 500 µm (A). Source data are provided as a Source Data file.

Supplementary Fig. 8 (associated with Fig. 7):

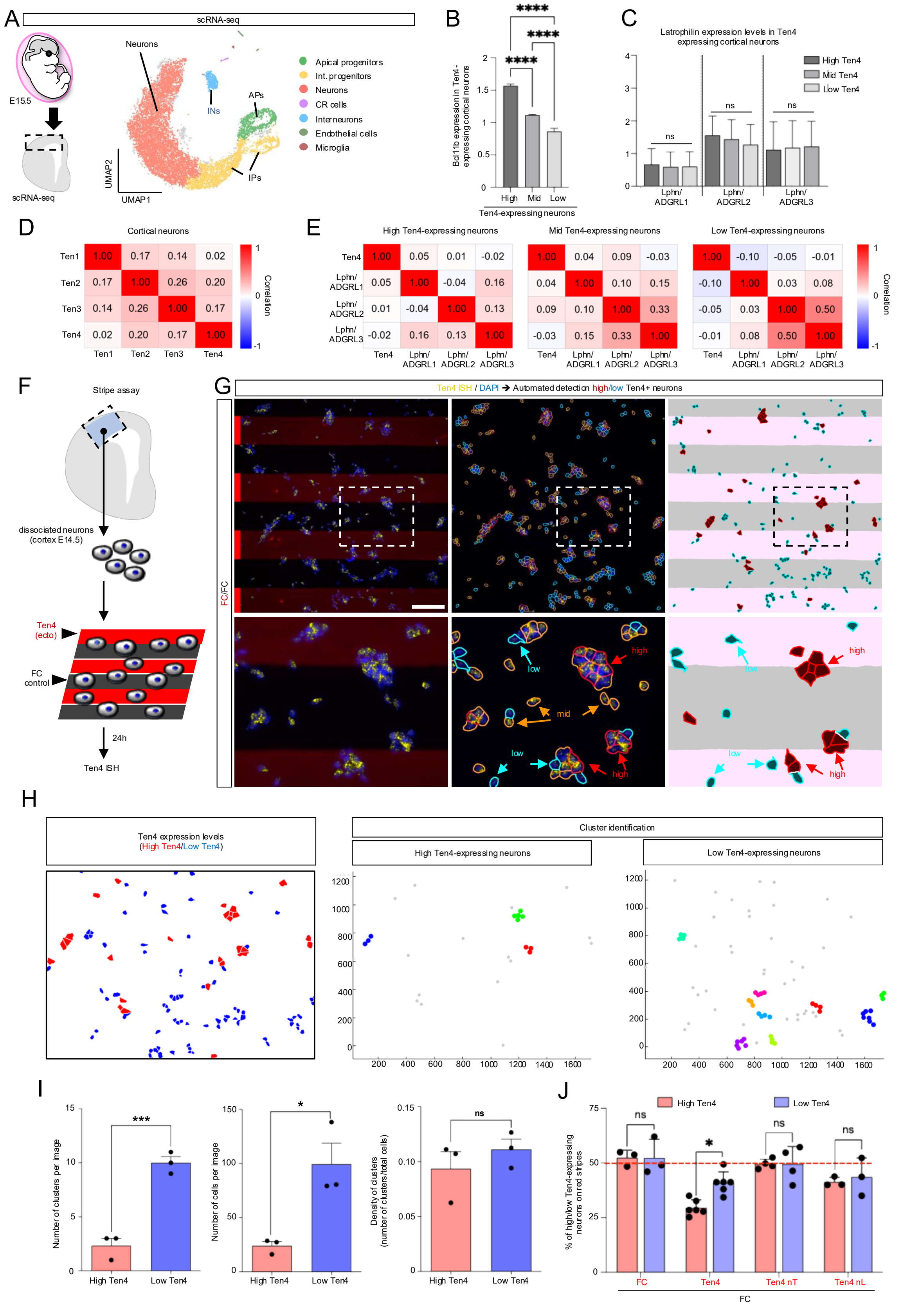

Supplementary Fig. 8. Stripe assay method using cortical neurons. A: Schematic and UMAP of an scRNA-seq dataset for murine cortex at E15.5. Cell types are coloured as

indicated in the legend (data source: GSE271794[122]) B: Bcl11b expression in cortical neurons grouped according to their Ten4 expression levels, as defined in Fig. 7E. C: Latrophilin mRNA expression levels in these different Ten4-expressing populations. D: Heatmap showing correlations for the mRNA expression levels of different Teneurin isoforms in cortical neurons (same data source as panel A). Red = positive high correlation, white = no correlation, blue = negative high correlation. E: As panel D but showing correlations for Ten4 and Latrophilin mRNA expression. We analysed these correlations separately for cells expressing different Ten4 levels. F: Overview of the stripe assay method. G: Snapshots taken from the automated analysis pipeline for Ten4 stripe assays. We used CellProfiler[114] to outline cells according to the Ten4 mRNA transcript levels detected using RNAscope (red = high, orange = mid, cyan = low). The percentage of high and low Ten4-expressing cells located on red stripes (highlighted in pink after thresholding) was quantified in ImageJ using a custom macro from a previous study[3]. As an example, Fc/Fc control data are shown. H: Example images of the cluster analysis used to compare high- and low-Ten4-expressing neurons. High-Ten4 cells are shown in red, and low-Ten4 cells are shown in blue. Cell clusters are identified based on the position of individual cells using a distance-based clustering method where a cluster was defined as a minimum of 3 cells spaced less than 20 µm. Middle and right images: clustered cells are indicated by different colours; cells that are not clustered are coloured in grey. I: Quantification of the number of clusters, cells per image and density of clusters. n=3 or more experiments per condition. J: Quantification of the results for the different stripe assay conditions used, here comparing low- *versus* high-Ten4 expressing neurons in each condition. n.s. = not significant. * $p<0.05$, *** $p<0.001$. Two-tailed unpaired Student's t test (panel I); one-way ANOVA test with Tukey's post hoc analysis (panel J). Scale bar represents 100 µm (panel G). All error bars shown are the SEM. Source data are provided as a Source Data file.

Supplementary table 1 related to Figure 1 and Supplementary Fig. 1. Cryo-EM processing statistics for the 3D maps.

	Teneurin2 A0B0 EMDB: EMD-51022	Teneurin2 A1B1 EMDB: EMD-50975	Teneurin2 A1B0 EMDB: EMD-50976	Teneurin2 A0B1 EMDB: EMD-51021
Magnification	105,000	105,000	105,000	150,000
Voltage (kV)	300	300	300	200
Dose per frame ($e^-/Å^2$)	1.04	1.06	1.04	1
Electron exposure ($e^-/Å^2$)	41.503	42.303	41.503	40
Defocus range (μm)	-1 to -2	-1 to -2	-1 to -2	-1.5 to -2.75
Pixel size (Å)	0.83	0.83	0.83	0.94
Symmetry imposed	C2	C2	C2	C2
Number of micrographs	6,108	8,975	5,136	5,212
Initial number of particles	4,649,892	6,799,146	3,672,845	1,037,437
Final number of particles	38,045	19,620	167,798	116,348
Map resolution (FSC 0.143)	2.82 Å	2.80 Å	2.55 Å	3.48 Å

As reported by cryoSPARC

Supplementary table 2 related to Figure 1 and Supplementary Fig. 1. Refinement statistics for the atomic models.

	Teneurin2 A0B0 PDB: 9G42	Teneurin2 A1B1 PDB: 9G2F	Teneurin2 A1B0 PDB: 9G2H	Teneurin2 A0B1 PDB: 9G41
Chains	4	4	2	2
Total number of atoms	27,564	28,160	22,241	22,275
Number of residues	3,444	3,504	2,732	2,740
R.m.s deviation bond lengths (Å)	0.004 (0)	0.004 (0)	0.003 (0)	0.003 (0)
R.m.s deviation bond angles (°)	0.713 (10)	0.701 (5)	0.595 (0)	0.594 (0)
Ramachandran plot favoured (%)	96.88%	96.46%	97.39%	97.14%
Ramachandran plot allowed (%)	3.12%	3.54%	2.61%	2.86%
Ramachandran plot outlier (%)	0%	0%	0%	0%
Rotamer outliers (%)	0.90%	1.45%	0.79%	0.67%
Clash score	3.83	3.68	3.69	3.52
Molprobity score	1.36	1.51	1.28	1.30
CC model vs data (mask)	0.84	0.85	0.83	0.78

As reported by PHENIX comprehensive cryo-EM validation and Molprobity.

Uncropped scans of all blots

Western blot from Extended Data Figure 3F

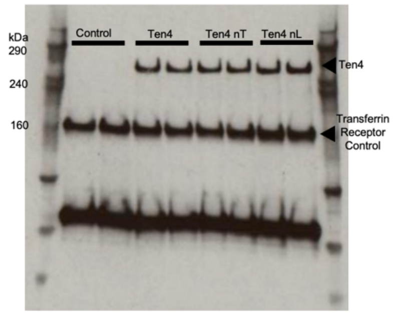

Western blot from Extended Data Figure 3I

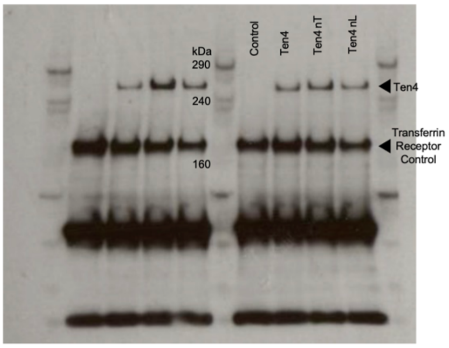

Western blot from Extended Data Figure 6B

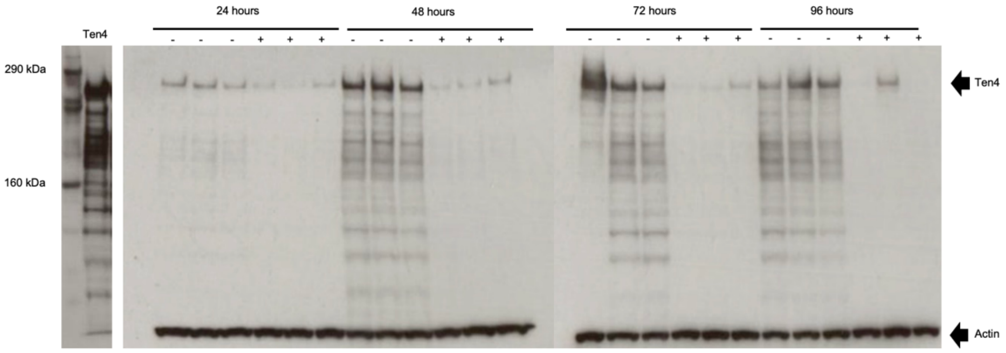

Supplement: Supplementary file 1 — Supplementary Information [file 41467_2026_71619_MOESM1_ESM.pdf]
