# Supplementary material for: Structurally exclusive Teneurin complexes orchestrate divergent programs in early cortical development
Source: Nat Commun. 2026 Apr 16;17:5292. doi: 10.1038/s41467-026-71619-1 (PMC13269823; doi:10.1038/s41467-026-71619-1)
Supplement: Supplementary file 2 — Description of Additional Supplementary Files [file 41467_2026_71619_MOESM2_ESM.pdf]

Description of Additional Supplementary Files

File name: Supplementary Data 1
Description: Oligonucleotide sequences used in this study, such as DNA primers, CRISPR, and shRNAs. Provided as .xlsx file (separate file).
