# Supplementary material for: Structurally exclusive Teneurin complexes orchestrate divergent programs in early cortical development
Source: Nat Commun. 2026 Apr 16;17:5292. doi: 10.1038/s41467-026-71619-1 (PMC13269823; doi:10.1038/s41467-026-71619-1)
Supplement: Supplementary file 4 — Reporting Summary [file 41467_2026_71619_MOESM4_ESM.pdf]

nature portfolio

Corresponding author(s): U. Valentin Naegerl, Daniel del Toro, Elena Seiradake

Last updated by author(s): Dec 18, 2025

Reporting Summary

Nature Portfolio wishes to improve the reproducibility of the work that we publish. This form provides structure for consistency and transparency in reporting. For further information on Nature Portfolio policies, see our Editorial Policies and the Editorial Policy Checklist.

Statistics

For all statistical analyses, confirm that the following items are present in the figure legend, table legend, main text, or Methods section.

n/a	Confirmed	
☐	☒	The exact sample size (*n*) for each experimental group/condition, given as a discrete number and unit of measurement
☐	☒	A statement on whether measurements were taken from distinct samples or whether the same sample was measured repeatedly
☐	☒	The statistical test(s) used AND whether they are one- or two-sided<br>*Only common tests should be described solely by name; describe more complex techniques in the Methods section.*
☒	☐	A description of all covariates tested
☐	☒	A description of any assumptions or corrections, such as tests of normality and adjustment for multiple comparisons
☐	☒	A full description of the statistical parameters including central tendency (e.g. means) or other basic estimates (e.g. regression coefficient) AND variation (e.g. standard deviation) or associated estimates of uncertainty (e.g. confidence intervals)
☐	☒	For null hypothesis testing, the test statistic (e.g. *F*, *t*, *r*) with confidence intervals, effect sizes, degrees of freedom and *P* value noted<br>*Give P values as exact values whenever suitable.*
☒	☐	For Bayesian analysis, information on the choice of priors and Markov chain Monte Carlo settings
☒	☐	For hierarchical and complex designs, identification of the appropriate level for tests and full reporting of outcomes
☒	☐	Estimates of effect sizes (e.g. Cohen's *d*, Pearson's *r*), indicating how they were calculated

Our web collection on statistics for biologists contains articles on many of the points above.

Software and code

Policy information about availability of computer code

Data collection	Cryo-EM data collection: EPU (ThermoFischer)<br>STED and fluorescence imaging: LasX
Data analysis	Cryo-EM data analysis: cryoSPARC (Punjani et al., 2017).<br>Initial model building: SWISSMODEL (Waterhouse et al., 2018)<br>Model refinement: PHENIX (Liebschner et al., 2019), Coot v.09.8.3<br>Model visualisation: ChimeraX (Pettersen et al., 2021)<br>Model prediction: Alphafold3<br>Prism, version 10 (Graphpad Software, USA)<br>ImageJ/Fiji, version 1.54f (Schindelin et al., 2012)<br>Rstudio v 4.0.1. (Rstudio USA)<br>Seurat package for R (Satija lab<br>Molecular dynamics:<br>Amber ff14SB<br>GROMACS 2023.2<br>Parrinello-Rahman barostat<br>MDAnalysis toolkit: Distance_array function

HydrogenBondAnalysis

For manuscripts utilizing custom algorithms or software that are central to the research but not yet described in published literature, software must be made available to editors and reviewers. We strongly encourage code deposition in a community repository (e.g. GitHub). See the Nature Portfolio guidelines for submitting code & software for further information.

Data

Policy information about availability of data

All manuscripts must include a data availability statement. This statement should provide the following information, where applicable:

- Accession codes, unique identifiers, or web links for publicly available datasets
- A description of any restrictions on data availability
- For clinical datasets or third party data, please ensure that the statement adheres to our policy

The electron density maps for the mouse Ten2 dimers have been deposited in the Electron Microscopy Data Bank under the accession codes: EMD-51022 (A0B0) (https://www.ebi.ac.uk/emdb/EMD-51022), EMD-50975 (A1B1) (https://www.ebi.ac.uk/emdb/EMD-50975), EMD-50976 (A1BO) (https://www.ebi.ac.uk/emdb/EMD-50976), and EMD-51021 (A0B1) (https://www.ebi.ac.uk/emdb/EMD-51021).The atomic coordinates and models resulting from the structural analysis of the aforementioned maps have been deposited on the Protein Data Bank under the following accession codes respectively: 9G42 (A0B0) (https://doi.org/10.2210/pdb9G42/pdb), 9G2F (A1B1) (https://doi.org/10.2210/pdb9G2F/pdb), 9G2H (A1B0) (https://doi.org/10.2210/pdb9G2H/pdb), and 9G41 (A0B1) (https://doi.org/10.2210/pdb9G41/pdb). The mass spectrometry data of the Teneurin mutants can be found in ProteomeXchange under the accession code: PXD058769. The Teneurin3 and 4 dimers shown in Extended Data Fig. 1 are available in the PDB: 8R50 (https://doi.org/10.2210/pdb8R50/pdb) and 7BAM (https://doi.org/10.2210/pdb7BAM/pdb). Single-cell RNA-seq data is available in NCBI GEO: GSE153164 (https://www.ncbi.nlm.nih.gov/geo/query/acc.cgi?acc=GSE153164) and GSE271794 (https://www.ncbi.nlm.nih.gov/geo/query/acc.cgi?acc=GSE271794). Molecular dynamics trajectories are available upon request. Source Data are provided with this paper.

Research involving human participants, their data, or biological material

Policy information about studies with human participants or human data. See also policy information about sex, gender (identity/presentation), and sexual orientation and race, ethnicity and racism.

Reporting on sex and gender	No research involving humans was performed.
Reporting on race, ethnicity, or other socially relevant groupings	No research involving humans was performed.
Population characteristics	No research involving humans was performed.
Recruitment	No research involving humans was performed.
Ethics oversight	No research involving humans was performed.

Note that full information on the approval of the study protocol must also be provided in the manuscript.

Field-specific reporting

Please select the one below that is the best fit for your research. If you are not sure, read the appropriate sections before making your selection.

- [x] Life sciences
- [ ] Behavioural & social sciences
- [ ] Ecological, evolutionary & environmental sciences

For a reference copy of the document with all sections, see nature.com/documents/nr-reporting-summary-flat.pdf

Life sciences study design

All studies must disclose on these points even when the disclosure is negative.

Sample size	Sample size calculations were not performed.
Data exclusions	No data was excluded.
Replication	Four (at least) or more replicates were performed for the cell aggregation data with similar results. For some conditions, two replicates were performed on the same date with the same original pool of electroporated cells, giving similar results. For all replicates, if possible, a positive control was performed with each condition alongside the replicate to ensure the electroporated cells were fine.<br>For cell binding experiments two technical replicates were performed with similar results. Positive and negative controls were performed alongside each condition.<br>Between two and four independent replicates were performed for the stripes assays. In each independent replicate, we performed at least two technical replicates. For the in utero electroporation experiments, at least three different brains were analyzed in each condition.

	For the shadow imaging experiments, three three different brains were analyzed in each condition and at least three control and three mutant neurons were analyzed in each brain. These sample sizes are common in the field and enough to provide high-quality data.
Randomization	Samples not randomized for cell aggregation experiments or cell binding experiments, as we needed to control for the overexpressed construct. Same cell sample was used to electroporate/transfect all conditions in each individual replicate. Randomization not performed for in vivo experiments as we needed to be able to identify the different conditions.
Blinding	Blinding not performed.

Reporting for specific materials, systems and methods

We require information from authors about some types of materials, experimental systems and methods used in many studies. Here, indicate whether each material, system or method listed is relevant to your study. If you are not sure if a list item applies to your research, read the appropriate section before selecting a response.

Materials & experimental systems

n/a	Involved in the study
☐	☒ Antibodies
☐	☒ Eukaryotic cell lines
☒	☐ Palaeontology and archaeology
☐	☒ Animals and other organisms
☒	☐ Clinical data
☒	☐ Dual use research of concern
☒	☐ Plants

Methods

n/a	Involved in the study
☒	☐ ChIP-seq
☒	☐ Flow cytometry
☒	☐ MRI-based neuroimaging

Antibodies

Antibodies used	Anti-HA; SIGMA-Aldrich; Cat#H3663; RRID: AB_262051<br>Anti-6xHis Tag; Thermo Fisher Scientific; Cat#372900; RRID: AB_2533309<br>Anti-Penta-His antibody; QIAGEN; Cat#34660; RRID: AB_2619735<br>Anti-mouse Alexa 488; Abcam; Cat#ab150117; RRID: AB_2688012<br>Anti-Mouse Cy3 secondary antibody; Abcam; Cat#ab97035; RRID: AB_10680176<br>Anti-Mouse Cy3 secondary antibody; Invitrogen; Cat#A10521; AB_10373848<br>Anti-mouse HRP secondary antibody; Abcam; Cat#ab6721; AB_955447<br>Anti-rabbit Alexa 488; Thermo Fisher Scientific; Cat#A-21206; AB_2535792<br>anti-BIII Tubulin rabbit antibody; Sigma; Cat#T2200-200UL; AB_262133<br>Goat anti-Human IgG (H+L) Cross-Adsorbed Secondary Antibody, Alexa Fluor 647; Thermo Fisher Scientific; Cat#A-11014; AB_1500628<br>anti-hFc; Jackson ImmunoResearch; Cat#62-8400; AB_2337530<br>ChromPure Human IgG, Fc fragment; Jackson ImmunoResearch; Cat#009-000-008; AB_2337046<br>Rabbit anti-BLBP, Millipore. Cat#ABN14; RRID: AB_10000325<br>Anti-rabbit Alexa 647; Thermo Fisher Scientific; Catalog # A-31573
Validation	All antibodies used in this study were used previously in other papers by us (Akkerman et al., 2022, del Toro et al., 2020, Tønessen et al., 2018) and have been extensively used in the field by others (check on the antibody registry for individual references). Details of the antibodies are available on the antibody registry and manufacturer's website. Dilutions and use for each antibody are detailed in the method section.

Eukaryotic cell lines

Policy information about cell lines and Sex and Gender in Research

Cell line source(s)	K562 cell line (ATCC; CCL-243; RRID: CVCL_0004)<br>HEK293T cell line (ATCC; CRL-3216; RRID: CVCL_0063)
Authentication	Cell lines were not authenticated after purchase from provider.
Mycoplasma contamination	All cells are mycoplasma negative.
Commonly misidentified lines<br>(See ICLAC register)	No commonly misidentified lines used.

Animals and other research organisms

Policy information about studies involving animals; ARRIVE guidelines recommended for reporting animal research, and Sex and Gender in Research

Laboratory animals	All mice were of C57BL/6 background. Pregnant females (approx. 3 months old) were purchased from Charles River and 3 days after surgery (embryonic day e16-18) they were euthanized by cervical dislocation to obtain embryonic brains. For cultures, pregnant females were sacrificed in the same way at embryonic day e14-15.
Wild animals	No wild animals were used.
Reporting on sex	In this study we only used pregnant females. As we worked with embryonic brains, we asume that in each experiment there will be an estimated representation of 50% males and 50% females, as this is the natural proportion of embryos.
Field-collected samples	All mice were housed with a 12h:12h light:dark cycle and food/water available ad libitum.
Ethics oversight	All animal experiments were used in accordance with the ethical guidelines (Declaration of Helsinki and NIH, publication no. 85-23, revised 1985, European Community Guidelines, and approved by the local ethical committee (University of Barcelona, 225/17 and Generalitat de Catalunya, 404/18).

Note that full information on the approval of the study protocol must also be provided in the manuscript.

Plants

Seed stocks	n/a
Novel plant genotypes	n/a
Authentication	n/a
