# Supplementary material for: Structurally exclusive Teneurin complexes orchestrate divergent programs in early cortical development
Source: Nat Commun. 2026 Apr 16;17:5292. doi: 10.1038/s41467-026-71619-1 (PMC13269823; doi:10.1038/s41467-026-71619-1)
Supplement: Supplementary file 5 — Transparent Peer Review file [file 41467_2026_71619_MOESM5_ESM.pdf]

nature portfolio

Peer Review File

Structurally Exclusive Teneurin Complexes Orchestrate Divergent Programs in Early Cortical Development

Corresponding Author: Professor Elena Seiradake

This file contains all reviewer reports in order by version, followed by all author rebuttals in order by version.

Version 0:

Reviewer comments:

Reviewer #1

(Remarks to the Author)
In this manuscript the authors discuss new Ten2 cryoEM structures with different splice inserts and with critical mutations that disrupt either Ten2 dimerization (nT) or its capability of binding LPHN (nL). They also use other state-of-the-art technologies such in vivo gene editing, and superresolution-microscopy to express Ten4 into embryonic mouse brain and then test the effect of the nT and nL mutants in neuronal development and cortical migration. The structural switch hypothesis between Ten4-Ten4 and Ten4-Lphn binding is fascinating. The paper is well written and the story is compelling and easy to follow. Overall this is a manuscript of exceptional high quality, novelty and insights.

Major concerns
None for the structural biology part of this manuscript, where my main expertise lies. This is really a first-rate manuscript form world-class scientists.

Minor concerns
In figure 1C and 1E is clear where splice insert B is, but I cannot discern where splice insert A is located. Can it be highlighted (it or its position) as well?

Page 7, line 219: “by the later cell-binding method” probably meant as “by the latter cell-binding method”

Reviewer #3

(Remarks to the Author)
The work by Berbeira-Santana and colleagues is an impressive study that bridges from structural biology and protein engineering to the molecular mechanisms underlying migration during cortical development and the effects on tissue architecture. The work presents valuable progress in the understanding of how teneurins, cellular adhesion molecules which impact cell migration and nervous system development, regulate distinct migration requirements during development.

The fact that the findings and hypotheses generated on the structural protein level are validated step by step in settings that progressively evolve from in silico and in vitro testing to the effect on native brain tissue, including genetic manipulation of the molecular interaction in a targeted manner, makes the study particularly comprehensive and powerful. The approach directly applies the insights on the structural mechanisms at the molecular level to perturb cellular interactions and testing the outcome in the developing animal.

The paper is well written and results are presented clearly. I fully support publication in Nature Communications.

Minor comments:
I found a few typos which should be easy to eliminate with another careful read before publication. E.g. in the base scope and RNA in situ hybridization sections (line 1105 onwards), it seems that fonts did not work out when specifying the thickness of the slices (10 microns vs. the 10 mm stated in the text). Similar for square micrometers (mm^2) in line 818. Also, on line 772: “Stipe assays and immunostaining“ should be „stripe“.

Reviewer #4

(Remarks to the Author)
During cortical migration, neurons interact with radial glial cells through cell adhesion molecules. Berbeira-Santana et al. showed that the cell adhesion molecule Teneurins interact homophilically in trans with Teneurin and heterophilically in trans with the adhesion GPCR Latrophilin. The authors produced Teneurin mutants with impaired in-trans Teneurin-Teneurin binding (nT) or Tenuerin4-Latrophilin binding (nL). They show that cortical neurons increase the expression level of Tenuerin4 during migration from the intermediate zone to the cortical plate. Based on the data using the nT and nL mutants, the authors suggest that Tenuerin4-Latrophilin interaction increases the association of neurons with radial glial cells and promotes neuronal migration to the cortical plate. On the other hand, Teneurin-Teneurin interaction reduced the association of neurons with radial glial cells. However, the data of the present paper are not clear-cut enough to explain their conclusions; some of them appear contradictory. Additionally, the conclusions of this paper are not clear in terms of advancing our understanding of the mechanism of cortical neuronal migration.

Major comments

1) Fig. 7F and H: From the data of the stripe assay, the authors conclude that “Ten4-Ten4 homophilic 'trans' interactions are repulsive in these neurons” (lines 357-358). However, the neurons expressing high levels of Ten4 aggregate more than neurons expressing low levels of Ten4 (Fig. 7F and Extended Data Fig. 7I), indicating that Ten4-Ten4 homophilic 'trans' interactions promote neuronal interactions. The cell-cell aggregation assay in Fig. 2C-F also support this. Thus, the authors’ main conclusion (lines 357-358) contradicts with these experimental data. As the basic function of cell adhesion molecules is to promote mechanical interaction between cells, the authors need to present convincing data supporting their conclusion that the Ten4-Ten4 interaction prevents the cell-cell interaction.

2) Fig. 6B and C: The authors conclude that “Neurons expressing Ten4 nT have increased contacts with RGC fibers compared to control neurons, while neurons expressing Ten4 nL have reduced contacts” (lines 320-322). However, since it is difficult to identify the neuron-BLBP contacts in Fig. 6B and C, I cannot evaluate this difference. Furthermore, the data in Fig. 6E appear to indicate that neurons expressing Ten4 nT have “reduced” contacts while neurons expressing Ten4 nL have “increased” contacts, namely the opposite results.

3) Lines 280-281: Introduction of the mutants nT and nL in these over-expression experiments resulted in significant “rescue”. The analyses in Fig. 5E and F are not rescue experiments. So, it is more accurate to describe the data as such: the introduction of the mutants nT and nL in these over-expression experiments “significantly reduced the inhibitory” effect of Ten4 over-expression.

4) Regarding the above data, the over-expressions of nT and nL mutants both inhibited neuronal migration (Fig. 5E and F). The authors suggest that the Tenuerin4-Latrophilin interaction and Teneurin-Teneurin interaction are required for cortical migration (lines 271-272). How do the over-expression of nT mutant, that promotes Tenuerin4-Latrophilin interaction, and the over-expression of nL mutant, that promotes Teneurin-Teneurin interaction, inhibit the migration?

5) The authors show that Tenuerin4 knockdown inhibits neuronal radial migration (Fig. 5C and D). However, Tenuerin4 over-expression also inhibits it (Fig. 5E and F). The authors need to explain these seemingly contradictory results. A recent paper emphasized the importance of weak cell adhesion suitable for rapid cell migration (https://advanced.onlinelibrary.wiley.com/doi/10.1002/advs.202502074). Such weak adhesion might explain the apparently contradictory results.

6) Other cell adhesion molecules, such as N-cadherin (CDH2), have been shown to play a role in cortical neuronal migration (https://www.frontiersin.org/journals/cell-and-developmental-biology/articles/10.3389/fcell.2020.588152/full). How does the present adhesion system cooperate with other cell adhesion molecules for neuronal migration?

7) Lines 46-47: The authors conclude “The results show how Ten4 orchestrates cortical migration by exclusive structural mechanisms, underpinning the integration of distinct migration programs”. For me, it is unclear what the key conclusion of this study is for understanding of the mechanism of cortical neuronal migration.

Minor comments

1) Is Fig. 4G described in the main text?

Reviewer #5

(Remarks to the Author)
I co-reviewed this manuscript with one of the reviewers who provided the listed reports. This is part of the Nature Communications initiative to facilitate training in peer review and to provide appropriate recognition for Early Career Researchers who co-review manuscripts.

Version 1:

Reviewer comments:

Reviewer #1

(Remarks to the Author)
My comments were addressed satisfactorily and I don't have any other concern.

Reviewer #3

(Remarks to the Author)
I do not have additional points and fully support publication of the manuscript.

Reviewer #4

(Remarks to the Author)
The authors addressed my comments in this version. However, it does not provide convincing data supporting their conclusion that the Ten4-Ten4 interaction prevents the cell-cell interaction. Please see below for my original concerns, the authors' responses, and my comments on their responses.

Original concern 1) Fig. 7F and H: From the data of the stripe assay, the authors conclude that "Ten4-Ten4 homophilic 'trans' interactions are repulsive in these neurons" (lines 357-358). However, the neurons expressing high levels of Ten4 aggregate more than neurons expressing low levels of Ten4 (Fig. 7F and Extended Data Fig. 7I), indicating that Ten4-Ten4 homophilic 'trans' interactions promote neuronal interactions. The cell-cell aggregation assay in Fig. 2C-F also support this. Thus, the authors' main conclusion (lines 357-358) contradicts with these experimental data. As the basic function of cell adhesion molecules is to promote mechanical interaction between cells, the authors need to present convincing data supporting their conclusion that the Ten4-Ten4 interaction prevents the cell-cell interaction.
Authors' answer 1) we thank the reviewer for raising this point… We therefore also calculated the density of clusters (number of clusters/number of cells), and this resulted in no significant difference between high- and low-Ten4 expressing neurons (see graph in the bottom right corner of the figure below). From this we conclude that, at least in these experiments, high Ten4 expression levels do not cause the neurons to aggregate significantly more than low Ten4 levels.
Comment 1) It would be clearer to say "High Ten4 expression levels do not inhibit neuron-neuron interaction (aggregation) significantly more than low Ten4 levels" instead of "High Ten4 expression levels do not cause the neurons to aggregate significantly more than low Ten4 levels". These cell-cell interaction data contrast with the authors' conclusion that "homophilic Ten4 interaction triggers cell-repulsion in Ten4-expressing neurons" (lines 1842-1843). The authors need to include these negative data in the manuscript and discuss them.
Comment 2) Regarding the cell-substrate interaction assay, Fig. 7F and H plot the data of high- and low-Ten4-expressing neurons separately. I recommend plotting them together in the same graph comparing high- and low-Ten4-expressing neurons. Is the percentage of high-Ten4-expressing neurons on the Ten4 stripe is significantly lower than the percentage of low-Ten4-expressing neurons? This information is essential for supporting the authors' model that increased Ten4 expression levels reduce RGC-attachment (Fig. 7I) and the conclusion that "homophilic Ten4 interaction triggers cell-repulsion in Ten4-expressing neurons" (lines 1842-1843).
In summary, these cell-cell interaction and cell-substrate interaction data are core to this paper, and the former do not support the authors' conclusion. Therefore, I recommend to increase the number of experiments (FC and Ten4nL; n = 3) to obtain more statistically convincing data.

Original concern 6) Other cell adhesion molecules, such as N-cadherin (CDH2), have been shown to play a role in cortical neuronal migration. How does the present adhesion system cooperate with other cell adhesion molecules for neuronal migration?
Authors' answer 6) thank you for another excellent comment and helpful reference…. we now include a brief relevant discussion on how Teneurin interactions may functionally interplay with classical adhesion systems, citing the paper on CDH2, in order to hint at these ideas (see lines 420-423).
Comment 3) It would be clearer in this paper to say "cadherins, which play key roles in cortical neuronal migration…" instead of "cadherins, which play key roles in cortical development…" (lines 421-422).

Original concern 7) Lines 46-47: The authors conclude "The results show how Ten4 orchestrates cortical migration by exclusive structural mechanisms, underpinning the integration of distinct migration programs". For me, it is unclear what the key conclusion of this study is for understanding of the mechanism of cortical neuronal migration.
Authors' answer 6) … The key conclusion is that, Teneurin4 orchestrates different stages of cortical migration by using a structural/functional switch between high-affinity Lphn interactions and low-affinity homophilic interactions...
Comment 4) This would be clearer to conclude in Abstract.

Reviewer #5

(Remarks to the Author)
I co-reviewed this manuscript with one of the reviewers who provided the listed reports. This is part of the Nature Communications initiative to facilitate training in peer review and to provide appropriate recognition for Early Career Researchers who co-review manuscripts.

Version 2:

Reviewer comments:

Reviewer #4

(Remarks to the Author)
The authors have adequately addressed my concerns.

Open Access This Peer Review File is licensed under a Creative Commons Attribution 4.0 International License, which permits use, sharing, adaptation, distribution and reproduction in any medium or format, as long as you give appropriate credit to the original author(s) and the source, provide a link to the Creative Commons license, and indicate if changes were made.
In cases where reviewers are anonymous, credit should be given to 'Anonymous Referee' and the source.
The images or other third party material in this Peer Review File are included in the article’s Creative Commons license, unless indicated otherwise in a credit line to the material. If material is not included in the article’s Creative Commons license and your intended use is not permitted by statutory regulation or exceeds the permitted use, you will need to obtain permission directly from the copyright holder.
To view a copy of this license, visit https://creativecommons.org/licenses/by/4.0/

REVIEWER COMMENTS

Reviewer #1 (Remarks to the Author):

In this manuscript the authors discuss new Ten2 cryoEM structures with different splice inserts and with critical mutations that disrupt either Ten2 dimerization (nT) or its capability of binding LPHN (nL). They also use other state-of-the-art technologies such in vivo gene editing, and superresolution-microscopy to express Ten4 into embryonic mouse brain and then test the effect of the nT and nL mutants in neuronal development and cortical migration. The structural switch hypothesis between Ten4-Ten4 and Ten4-Lphn binding is fascinating. The paper is well written and the story is compelling and easy to follow. Overall this is a manuscript of exceptional high quality, novelty and insights.

Authors: we thank the reviewer for the positive assessment of the results and we address the remaining concerns below.

Major concerns
None for the structural biology part of this manuscript, where my main expertise lies. This is really a first-rate manuscript form world-class scientists.

Authors: thank you.

Minor concerns
In figure 1C and 1E is clear where splice insert B is, but I cannot discern where splice insert A is located. Can it be highlighted (it or its position) as well?

Authors: thank you for this comment. Splice site A is located between the EGF domains 7 and 8, which are in a part of the protein that is not resolved in any of the cryo-EM maps. We have now indicated the location of both splice sites in panel A, (Fig. 1A) which we hope makes this clearer.

Page 7, line 219: “by the later cell-binding method” probably meant as “by the latter cell-binding method”

Authors: thank you for spotting this typo, we have corrected it.

Reviewer #3 (Remarks to the Author):

The work by Berbeira-Santana and colleagues is an impressive study that bridges from structural biology and protein engineering to the molecular mechanisms underlying migration during cortical development and the effects on tissue architecture. The work

presents valuable progress in the understanding of how teneurins, cellular adhesion molecules which impact cell migration and nervous system development, regulate distinct migration requirements during development.

The fact that the findings and hypotheses generated on the structural protein level are validated step by step in settings that progressively evolve from in silico and in vitro testing to the effect on native brain tissue, including genetic manipulation of the molecular interaction in a targeted manner, makes the study particularly comprehensive and powerful. The approach directly applies the insights on the structural mechanisms at the molecular level to perturb cellular interactions and testing the outcome in the developing animal.

The paper is well written and results are presented clearly. I fully support publication in Nature Communications.

Authors: Thank you for this excellent summary and assessment. We are grateful and will admit that we are immensely proud of the work ☺.

Minor comments:
I found a few typos which should be easy to eliminate with another careful read before publication. E.g. in the base scope and RNA in situ hybridization sections (line 1105 onwards), it seems that fonts did not work out when specifying the thickness of the slices (10 microns vs. the 10 mm stated in the text). Similar for square micrometers (mm^2) in line 818. Also, on line 772: "Stipe assays and immunostaining" should be „stripe".

Authors: thank you for spotting these formatting errors and typos. We have fixed them.

Reviewer #4 (Remarks to the Author):

During cortical migration, neurons interact with radial glial cells through cell adhesion molecules. Berbeira-Santana et al. showed that the cell adhesion molecule Teneurins interact homophilically in trans with Teneurin and heterophilically in trans with the adhesion GPCR Latrophilin. The authors produced Teneurin mutants with impaired in-trans Teneurin-Teneurin binding (nT) or Tenuerin4-Latrophilin binding (nL). They show that cortical neurons increase the expression level of Tenuerin4 during migration from the intermediate zone to the cortical plate. Based on the data using the nT and nL mutants, the authors suggest that Tenuerin4-Latrophilin interaction increases the association of neurons with radial glial cells and promotes neuronal migration to the cortical plate. On the other hand, Teneurin-Teneurin interaction reduced the association of neurons with radial glial cells. However, the data of the present paper are not clear-cut enough to explain their conclusions; some of them appear contradictory. Additionally, the conclusions of this paper are not clear in terms of advancing our understanding of the mechanism of cortical neuronal migration.

Authors: thank you for assessing our manuscript. We provide additional analysis and clarification below to address the comments.

Major comments

1) Fig. 7F and H: From the data of the stripe assay, the authors conclude that "Ten4-Ten4 homophilic 'trans' interactions are repulsive in these neurons" (lines 357-358). However, the neurons expressing high levels of Ten4 aggregate more than neurons expressing low levels of Ten4 (Fig. 7F and Extended Data Fig. 7I), indicating that Ten4-Ten4 homophilic 'trans' interactions promote neuronal interactions. The cell-cell aggregation assay in Fig. 2C-F also support this. Thus, the authors' main conclusion (lines 357-358) contradicts with these experimental data. As the basic function of cell adhesion molecules is to promote mechanical interaction between cells, the authors need to present convincing data supporting their conclusion that the Ten4-Ten4 interaction prevents the cell-cell interaction.

Authors: we thank the reviewer for raising this point. Regarding the stripe assays, we take the comment to be based on the example images shown in Figure 7 and S7. To test whether there is a significant difference in the aggregation propensity for the "high" versus the "low" Ten4 expressing neurons in our stripe assays, we analysed our Fc/Fc control stripe data set systematically. We defined "clusters" as having at least three cells, spaced less than 20 μm apart. Counting the number of clusters for each high- and low-Ten4 expressing neurons resulted in a higher number of clusters for the low-Ten4-expressing neurons (see figure panels A and B below). However, on average, the number of low-Ten4-expressing neurons is higher than that of high-Ten4-expressing neurons, per image (see panel B in figure below). We therefore also calculated the density of clusters (number of clusters/number of cells), and this resulted in no significant difference between high- and low-Ten4 expressing neurons (see graph in the bottom right corner of the figure below). From this we conclude that, at least in these experiments, high Ten4 expression levels do not cause the neurons to aggregate significantly more than low Ten4 levels.

Regarding the approach, we have used this methodology to calculate neuronal clusters previously[1] and have also described that neuronal aggregation occurs during stripe assays in a previous publication[2].

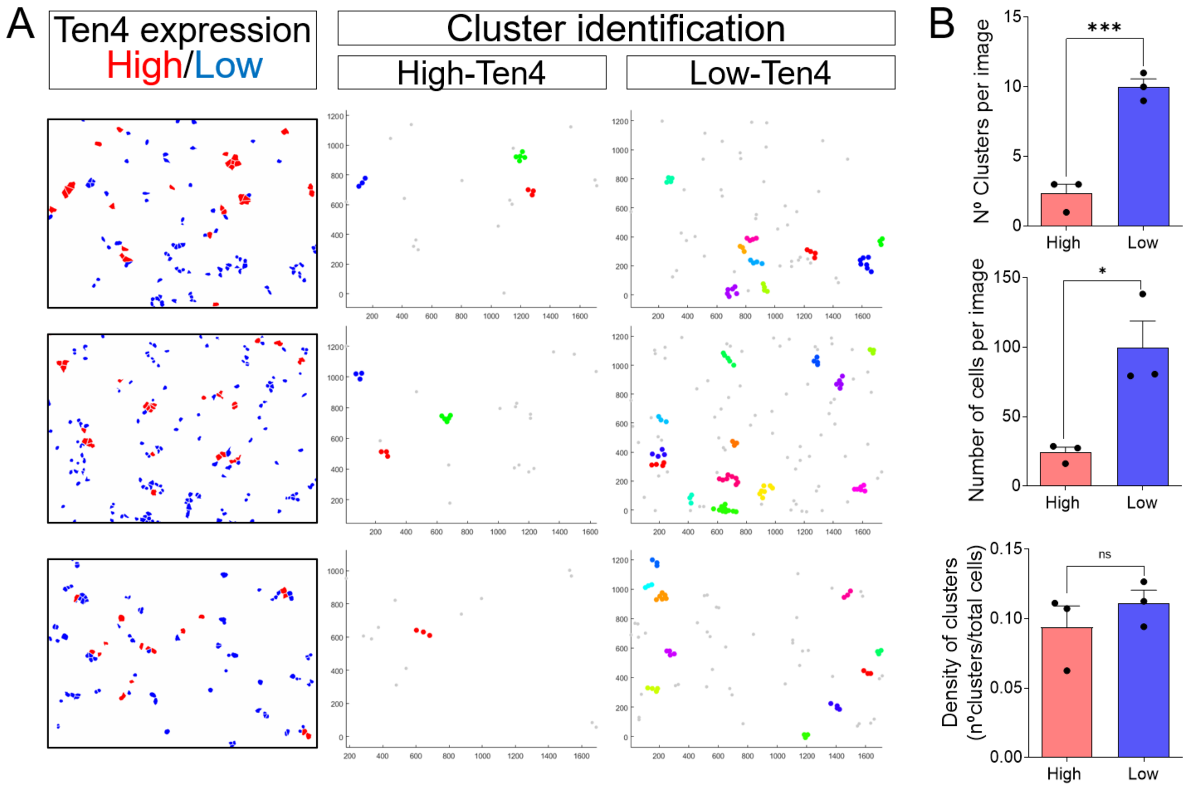

Figure: *Example images and quantification of the cluster analysis used to compare high- and low- Ten4-expressing neurons. A: High-Ten4 cells are shown in red, and low-Ten4 cells are shown in blue. Cell clusters are identified based on the position of individual cells using a distance-based clustering method (middle panels; a cluster was defined as a minimum of 3 cells spaced less than 20 µm). Neuron clusters for both high- and low- Ten4 cells are marked with different colours. Cells that are not clustered are coloured in grey. B: Quantification of the number of clusters, cells per image and density of clusters. n=3 or more experiments per condition. n.s. = not significant, *p < 0.05, ***p < 0.001, Student's t test.*

Should the reviewers wish us to include this analysis in the supplementary materials, then we would the happy to do so.

With regards to the comment that Ten4-expressing cells aggregate in our cell-cell aggregation assay, please note that we use non-adherent cells which cannot "repel" each other as they are not bound to a substrate. There is nothing to provide traction as the cells are in suspension. Thus, what determines the clustering in this assay is whether receptors form molecular interactions (or not) across cells. As "molecular adhesion" is a pre-requisite for both cell-to-cell adhesion and repulsion, it is accepted that repulsive receptors also produce adhesion in this assay. For example, we have shown previously that Unc5, which is a classical repulsive guidance receptor, produces strong cell-cell adhesion in this assay by interacting with its ligand GPC3[3].

2) Fig. 6B and C: The authors conclude that "Neurons expressing Ten4 nT have increased contacts with RGC fibers compared to control neurons, while neurons expressing Ten4

nL have reduced contacts" (lines 320-322). However, since it is difficult to identify the neuron-BLBP contacts in Fig. 6B and C, I cannot evaluate this difference. Furthermore, the data in Fig. 6E appear to indicate that neurons expressing Ten4 nT have "reduced" contacts while neurons expressing Ten4 nL have "increased" contacts, namely the opposite results.

Authors: we thank the reviewer for this comment. We have changed the panels in Fig.6 to make it easier to understand how we analysed the neuron-BLBP contacts (see below and the amended Fig. 6C). As stated in the text, our analysis shows that CP neurons harbouring the Ten4 nT mutation associate more with RGC fibres compared to those harbouring the nL mutation, on average. This would suggest that Ten4-Ten4 interaction reduces the association with neurons, whilst Ten4-Lphn increases it. This can be seen in the quantification shown in panel D, where we binned neurons into those with high, medium or low amounts of RGC staining in their close vicinity (see methods). Panel E essentially shows the same result, but as cumulative frequency plots. For example, a point in the graph with the coordinates x=4 (μm^2) and y=59 (%) means that 59% of the frequencies (measurements) have an area equal or less than 4 μm^2. Because cumulative curves rise faster when values are smaller, a left-shifted curve (as seen for Ten4 nL) indicates overall smaller neuron–BLBP contact areas, whereas a right-shifted curve (as seen for Ten4 nT) indicates larger contact areas.

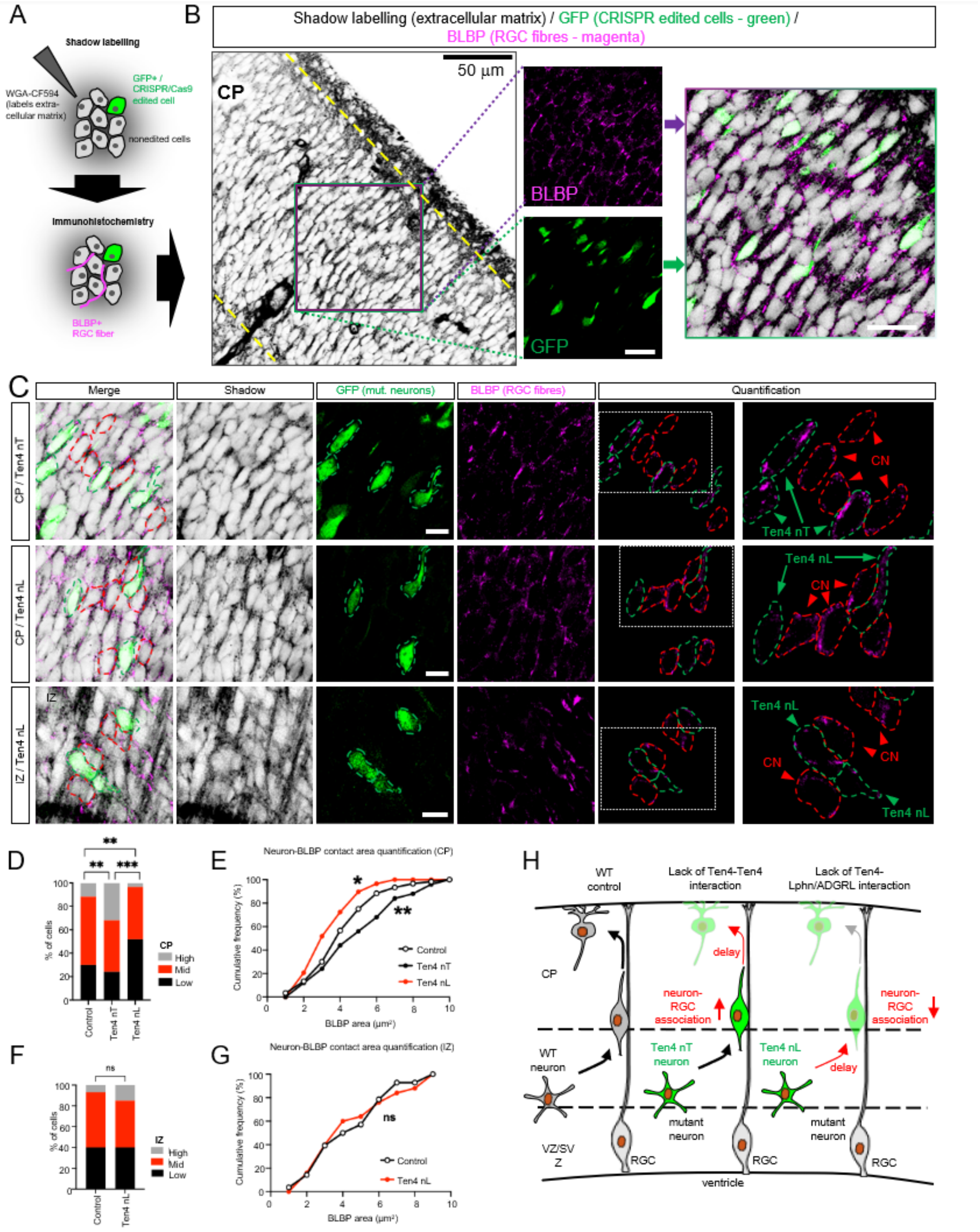

New Figure 6. Ten4 regulates neuron-RGC association via two distinct mechanisms.
A: Diagram depicting the shadow and immunohistochemistry labelling strategy for brain slices collected after IUE using CRISPR/Cas9 reagents to target Tenm4 (as in Fig. 5G). Black = extracellular matrix (Shadow labelling), magenta = anti-BLBP, green =GFP-positive CRISPR-targeted neurons. Wild type neurons are white. B: Example of a labelled brain slice with channels merged, following the strategy outlined in panel A. C: Shadow labelling (black) on electroporated coronal sections with GFP-positive CRISPR-targeted neurons

*(green) immunostained for BLBP (RGC fibres, magenta). Green dashed lines indicate the contour area assigned to each GFP-positive neuron, whereas red dashed lines represent the contour of adjacent control neurons identified through shadow imaging (white neurons). BLBP staining was quantified within each identified contour area (quantification panels). Area in white dashed rectangle is shown with higher magnification on the right. Red and green arrows indicate BLBP staining in control and CRISPR-targeted neurons respectively. D-G: Quantification of experiments as shown in panel C. We expanded the outlined somata of cells by 1 µm and quantified the BLBP labelling within these areas for different cells (control, nL and nT). The neurons were separated into different populations depending on the levels of RGC staining in their vicinity: low (0-3.5 µm2), mid (3.5-6.5 µm2), and high (6.5-10 µm2). The plots display the fraction of cells and cumulative frequency distributions in the CP. N=3 brains. H: Summary diagram showing the observed phenotypes. n.s. = not significant. *p<0.05, **p < 0.01, ***p<0.001. Chi-square contingency analysis (D,F) and Kolmogorov-Smirnov test (E,G). Scale bars represent 50 um (B) or 20 µm (C).*

3) Lines 280-281: Introduction of the mutants nT and nL in these over-expression experiments resulted in significant “rescue”. The analyses in Fig. 5E and F are not rescue experiments. So, it is more accurate to describe the data as such: the introduction of the mutants nT and nL in these over-expression experiments “significantly reduced the inhibitory” effect of Ten4 over-expression.

Authors thank you for suggesting the improved wording, we have replaced the original phrase with the one suggested. We still use the word “rescue” in the next sentence but added the word “apparent” (i.e. “apparent rescue”), in response to the reviewer’s comment.

4) Regarding the above data, the over-expressions of nT and nL mutants both inhibited neuronal migration (Fig. 5E and F). The authors suggest that the Tenuerin4-Latrophilin interaction and Teneurin-Teneurin interaction are required for cortical migration (lines 271-272). How do the over-expression of nT mutant, that promotes Tenuerin4-Latrophilin interaction, and the over-expression of nL mutant, that promotes Teneurin-Teneurin interaction, inhibit the migration?

Authors: thank you for this comment. In these experiments, we are overexpressing the proteins (mutant or WT) in a system where the proteins are also expressed endogenously. The levels of endogenous expression are finely tuned, depending on the required functions, as indeed we show in this paper, where different levels of the same receptor underpin different functional states. Overexpression, here using a constitutively active chicken actin promotor, boosts the expression levels above the physiological level, leading to impaired migration. The reason for this impairment is, in part, due to interactions of the overexpressed receptor with itself (as the nT mutant shows) or with Latrophilin (as the nL mutant shows). Neither of these mutants remove all the effects that the overexpression has on the system, which is why migration is still impaired, albeit at a lower level compared to the “WT Ten4 control” overexpression. We point out that we

also performed CRISPR/Cas9-based experiments, in which we mutate endogenous proteins to introduce the nT and nL mutations, thus circumventing the need for overexpression (Fig. 5E and F). The results of the overexpression experiments are in good agreement with the CRISPR/Cas9-based experiments both pointing to Ten4-Lphn interactions dominating at the IZ/CP boundary, whilst Ten4-Ten4 interactions are important in the upper CP.

5) The authors show that Tenuerin4 knockdown inhibits neuronal radial migration (Fig. 5C and D). However, Tenuerin4 over-expression also inhibits it (Fig. 5E and F). The authors need to explain these seemingly contradictory results. A recent paper emphasized the importance of weak cell adhesion suitable for rapid cell migration (https://advanced.onlinelibrary.wiley.com/doi/10.1002/advs.202502074). Such weak adhesion might explain the apparently contradictory results.

Authors: We are grateful to the reviewer for pointing out this informative paper, which we now cite in the discussion to illustrate that finely tuned adhesive cell-traction is required for cell migration (lines 391-395). Indeed, several studies have shown that both very high or low adhesion impairs cell migration[4,5]. This is consistent with the observation that both loss and overexpression of Ten4 impair cell migration: the KO reduces interactions, whereas overexpression likely increases them due to receptor levels exceeding the physiological range. We and others have shown that both loss and overexpression of proteins involved in cell migration can impair this process, including FLRTs and Unc5s[6],Ten2[2] and Rnd2[7]. To clarify the Ten4 overexpression phenotype, we now state in the results section (line 281): "This suggests that excessive levels of Ten4 are detrimental to cell migration, as has been observed for other receptors involved in this process[2,6]."

6) Other cell adhesion molecules, such as N-cadherin (CDH2), have been shown to play a role in cortical neuronal migration (https://www.frontiersin.org/journals/cell-and-developmental-biology/articles/10.3389/fcell.2020.588152/full). How does the present adhesion system cooperate with other cell adhesion molecules for neuronal migration?

Authors: thank you for another excellent comment and helpful reference. This comment echoes a discussion that was triggered at the EMBO workshop of molecular neurobiology (2022), when an audience member asked why so many guidance receptors trigger "repulsive signalling". The emerging discussion revolved around whether a major function of cell guidance receptors is to counter-balance the strong cell adhesion mediated by classical cell adhesion molecules such as cadherins and integrins. Such a need to reduce "adhesion" could explain why most guidance receptor signalling appears to trigger cell repulsion in migrating cells. Indeed, using single cell sequencing data, we performed a correlation analysis between Teneurins and N-cadherin (CDH2) and cadherin 4 (CDH4), which also participates in cortical migration[8,9]. Interestingly, Ten4 shows a modest but positive correlation with both CDH2 and CDH4, whereas Ten2, also implicated in cortical migration[2], shows a positive correlation with CDH2. In both cases,

these correlations are higher than those observed for other Teneurins, such as Ten1 and Ten3 (see figure below).

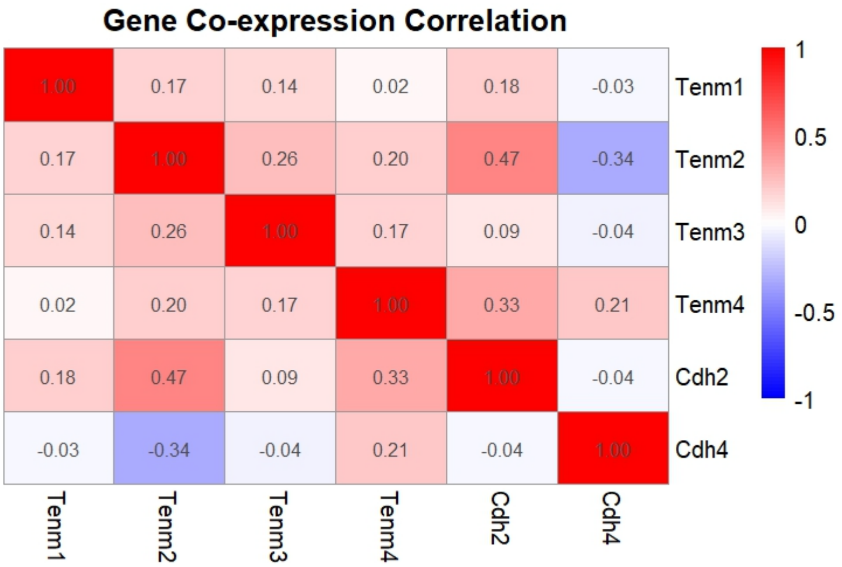

Figure: Heatmap showing correlations for the mRNA expression levels of different Teneurin isoforms, CDH2 and CDH4 in cortical neurons (same data source as Extended Data Figure 7F-G). Red = positive high correlation, white = no correlation, blue = negative high correlation. Note that the gene nomenclature for Teneurins are Tenm1-4. Data source: GSE271794

Mechanistically, several lines of evidence suggest that Teneurin interactions could converge on pathways known to regulate cadherin surface levels and adhesion. For example, Teneurin C-terminal associated peptides are known to regulate ERK activity, which is a well-established modulator of CDH2 protein levels during neuron migration[10,11]. We previously reported that Teneurins form a ternary complex with Latrophilins and FLRTs during cortical migration[2]. Latrophilin signalling promotes MAPK signalling to enhance caveolin- and dynamin-dependent receptor endocytosis[12], and through cAMP/Rap1 pathways, inhibits focal adhesion formation[13]. Notably, both Rap1 signalling and endocytic pathways are established control points for cadherin turnover at the plasma membrane[8]. In addition, FLRTs have been shown to regulate cell adhesion by controlling cadherin surface levels through dynamin-dependent endocytosis via Rnd1[14]. Moreover, FLRTs interact with FGF receptors[15], which are known to associate with CDH2 in cis to stimulate Erk1/2 phosphorylation during neuron migration[16].

Whilst the potential crosstalk between Teneurin interactions and classical cell adhesion molecules remains to be investigated in future work, we now include a brief relevant discussion on how Teneurin interactions may functionally interplay with classical adhesion systems, citing the paper on CDH2, in order to hint at these ideas (see lines 420-423). We have kept the discussion of this point short for now, in order to not add too much length to our already bulky paper, but if the reviewers would advise us to expand on this point using some of the data and discussion presented above, we would be happy to do so.

7) Lines 46-47: The authors conclude "The results show how Ten4 orchestrates cortical migration by exclusive structural mechanisms, underpinning the integration of distinct migration programs". For me, it is unclear what the key conclusion of this study is for understanding of the mechanism of cortical neuronal migration.

Authors: thank you for asking us to clarify this. The key conclusion is that, Teneurin4 orchestrates different stages of cortical migration by using a structural/functional switch between high-affinity Lphn interactions and low-affinity homophilic interactions. To the best of our knowledge, this is the first time that anyone has shown how the same receptor orchestrates two subsequent migration stages of the same cells, simply by switching its receptor/ligand interactions. It provides a good example of how relatively few guidance receptors underpin biological complexity during development.

Minor comments

1) Is Fig. 4G described in the main text?

Authors: we thank the reviewer for this comment, and we have included a call for Figure 4G in the main text (line 253).

Reviewer #5 (Remarks to the Author):

I co-reviewed this manuscript with one of the reviewers who provided the listed reports. This is part of the Nature Communications initiative to facilitate training in peer review and to provide appropriate recognition for Early Career Researchers who co-review manuscripts.

Authors: we thank the reviewer for their time and input to the revision process!

References:

1. Del Toro, D., Ruff, T., Cederfjäll, E., Villalba, A., Seyit-Bremer, G., Borrell, V., & Klein, R. (2017). Regulation of cerebral cortex folding by controlling neuronal migration via FLRT adhesion molecules. Cell, 169(4), 621-635. https://doi.org/10.1016/j.cell.2017.04.012
2. del Toro, D., Carrasquero-Ordaz, M.A., Chu, A., Ruff, T., Shahin, M., Jackson, V.A., Chavent, M., Berbeira-Santana, M., Seyit-Bremer, G., Brignani, S., et al. (2020). Structural Basis of Teneurin-Latrophilin Interaction in Repulsive Guidance of Migrating Neurons. Cell *180*, 323-339.e19. https://doi.org/10.1016/j.cell.2019.12.014.
3. Akkermans, O., Delloye-Bourgeois, C., Peregrina, C., Carrasquero-Ordaz, M., Kokolaki, M., Berbeira-Santana, M., Chavent, M., Reynaud, F., Raj, R., Agirre, J., et al. (2022).

GPC3-Unc5 receptor complex structure and role in cell migration. Cell *185*. https://doi.org/10.1016/j.cell.2022.09.025.
4. Rohani N, Canty L, Luu O, Fagotto F, Winklbauer R. EphrinB/EphB signaling controls embryonic germ layer separation by contact-induced cell detachment. PLoS Biol. 2011 Mar;9(3):e1000597. https://doi.org/10.1371/journal.pbio.1000597.
5. Wen JW, Winklbauer R. Ingression-type cell migration drives vegetal endoderm internalisation in the Xenopus gastrula. Elife. 2017 Aug 10;6:e27190. https://doi.org/10.7554/eLife.27190.
6. Seiradake, E., del Toro, D., Nagel, D., Cop, F., Härtl, R., Ruff, T., Seyit-Bremer, G., Harlos, K., Border, E.C., Acker-Palmer, A., et al. (2014). FLRT Structure: Balancing Repulsion and Cell Adhesion in Cortical and Vascular Development. Neuron 84, 370–385. https://doi.org/10.1016/j.neuron.2014.10.008.
7. Heng, JT., Nguyen, L., Castro, D. et al. Neurogenin 2 controls cortical neuron migration through regulation of Rnd2. Nature 455, 114–118 (2008). https://doi.org/10.1038/nature07198.
8. Martinez-Garay I. Molecular Mechanisms of Cadherin Function During Cortical Migration. Front Cell Dev Biol. 2020 Sep 15;8:588152. https://doi.org/10.3389/fcell.2020.588152.
9. Martinez-Garay I, Gil-Sanz C, Franco SJ, Espinosa A, Molnár Z, Mueller U. Cadherin 2/4 signaling via PTP1B and catenins is crucial for nucleokinesis during radial neuronal migration in the neocortex. Development. 2016 Jun 15;143(12):2121-34. https://doi.org/10.3389/10.1242/dev.132456.
10. Chand D, Song L, deLannoy L, Barsyte-Lovejoy D, Ackloo S, Boutros PC, Evans K, Belsham DD, Lovejoy DA. C-Terminal region of teneurin-1 co-localizes with dystroglycan and modulates cytoskeletal organization through an extracellular signal-regulated kinase-dependent stathmin- and filamin A-mediated mechanism in hippocampal cells. Neuroscience. 2012 Sep 6;219:255-70. https://doi.org/10.1016/j.neuroscience.2012.05.069.
11. Hor CHH, Goh ELK. Rab23 Regulates Radial Migration of Projection Neurons via N-cadherin. Cereb Cortex. 2018 Apr 1;28(4):1516-1531. https://doi.org/10.1093/cercor/bhy018.
12. Huang WR, Wu YY, Liao TL, Nielsen BL, Liu HJ. Cell Entry of Avian Reovirus Modulated by Cell-Surface Annexin A2 and Adhesion G Protein-Coupled Receptor Latrophilin-2 Triggers Src and p38 MAPK Signaling Enhancing Caveolin-1- and Dynamin 2-Dependent Endocytosis. Microbiol Spectr. 2023 Jun 15;11(3):e0000923. https://doi.org/10.1128/spectrum.00009-23.
13. Camillo C, Facchinello N, Villari G, Mana G, Gioelli N, Sandri C, Astone M, Tortarolo D, Clapero F, Gays D, Oberkersch RE, Arese M, Tamagnone L, Valdembri D, Santoro MM, Serini G. LPHN2 inhibits vascular permeability by differential control of endothelial cell adhesion. J Cell Biol. 2021 Nov 1;220(11):e202006033. https://doi.org/10.1083/jcb.202006033.

14. Ogata S, Morokuma J, Hayata T, Kolle G, Niehrs C, Ueno N, Cho KW. TGF-beta signaling-mediated morphogenesis: modulation of cell adhesion via cadherin endocytosis. Genes Dev. 2007 Jul 15;21(14):1817-31. https://doi.org/10.1101/gad.1541807.
15. Böttcher RT, Pollet N, Delius H, Niehrs C. The transmembrane protein XFLRT3 forms a complex with FGF receptors and promotes FGF signalling. Nat Cell Biol. 2004 Jan;6(1):38-44. https://doi.org/10.1038/ncb1082.
16. Kon E, Calvo-Jiménez E, Cossard A, Na Y, Cooper JA, Jossin Y. N-cadherin-regulated FGFR ubiquitination and degradation control mammalian neocortical projection neuron migration. Elife. 2019 Oct 2;8:e47673. https://doi.org/10.7554/eLife.47673.

REVIEWER COMMENTS

Reviewer #1 (Remarks to the Author):

My comments were addressed satisfactorily and I don't have any other concern.

Authors: we are happy that the reviewer is satisfied with the manuscript

Reviewer #3 (Remarks to the Author):

I do not have additional points and fully support publication of the manuscript.

Authors: we are happy that the reviewer is satisfied with the manuscript

Reviewer #4 (Remarks to the Author):

The authors addressed my comments in this version. However, it does not provide convincing data supporting their conclusion that the Ten4-Ten4 interaction prevents the cell-cell interaction. Please see below for my original concerns, the authors' responses, and my comments on their responses.

Original concern 1) Fig. 7F and H: From the data of the stripe assay, the authors conclude that "Ten4-Ten4 homophilic 'trans' interactions are repulsive in these neurons" (lines 357-358). However, the neurons expressing high levels of Ten4 aggregate more than neurons expressing low levels of Ten4 (Fig. 7F and Extended Data Fig. 7I), indicating that Ten4-Ten4 homophilic 'trans' interactions promote neuronal interactions. The cell-cell aggregation assay in Fig. 2C-F also support this. Thus, the authors' main conclusion (lines 357-358) contradicts with these experimental data. As the basic function of cell adhesion molecules is to promote mechanical interaction between cells, the authors need to present convincing data supporting their conclusion that the Ten4-Ten4 interaction prevents the cell-cell interaction.

Authors' answer 1) we thank the reviewer for raising this point... We therefore also calculated the density of clusters (number of clusters/number of cells), and this resulted in no significant difference between high- and low-Ten4 expressing neurons (see graph in the bottom right corner of the figure below). From this we conclude that, at least in these experiments, high Ten4 expression levels do not cause the neurons to aggregate significantly more than low Ten4 levels.

Comment 1) It would be clearer to say "High Ten4 expression levels do not inhibit neuron-neuron interaction (aggregation) significantly more than low Ten4 levels" instead of "High Ten4 expression levels do not cause the neurons to aggregate significantly more than low Ten4 levels". These cell-cell interaction data contrast with the authors' conclusion that "homophilic Ten4 interaction triggers cell-repulsion in Ten4-expressing

neurons" (lines 1842-1843). The authors need to include these negative data in the manuscript and discuss them.

Authors: We agree that this point required clarification. We have now included the cluster density analysis in a new Extended Data Figure 8 and revised the results section to state: "We previously showed that neurons tend to aggregate over time in this assay[1]. High Ten4 expression did not significantly inhibit neuron–neuron aggregation compared to low Ten4 expression (Extended Data Fig. 8H, I)" (lines 361 ff).

We note that the stripe assay is not designed to assess cell–cell aggregation, but rather to measure neuronal responses to substrate-bound cues. In this assay, we consistently observe avoidance of Ten4-coated stripes by high Ten4–expressing neurons, indicating that substrate-bound Ten4 elicits a repulsive response. Thus, while high Ten4–expressing neurons do not prevent the spontaneous aggregation that neurons show over time in this assay, it does mediate repulsion from Ten4-coated surfaces.

Comment 2) Regarding the cell-substrate interaction assay, Fig. 7F and H plot the data of high- and low-Ten4-expressing neurons separately. I recommend plotting them together in the same graph comparing high- and low-Ten4-expressing neurons. Is the percentage of high-Ten4-expressing neurons on the Ten4 stripe is significantly lower than the percentage of low-Ten4-expressing neurons? This information is essential for supporting the authors' model that increased Ten4 expression levels reduce RGC-attachment (Fig. 7I) and the conclusion that "homophilic Ten4 interaction triggers cell-repulsion in Ten4-expressing neurons" (lines 1842-1843).
In summary, these cell-cell interaction and cell-substrate interaction data are core to this paper, and the former do not support the authors' conclusion. Therefore, I recommend to increase the number of experiments (FC and Ten4nL; n = 3) to obtain more statistically convincing data.

Authors: We thank the reviewer for this constructive suggestion. As recommended, we have now plotted high- and low-Ten4-expressing neurons together in the same graph to directly compare both conditions in a new Extended Data Figure 8. This new graph shows that high Ten4-expressing neurons display significantly lower percentage on Ten4 stripes compared to low-Ten4 expressing cells ($p=0.0281$). The data indicate that increased Ten4 expression enhances the repulsive response, which is consistent with our model that higher Ten4 levels reduce RGC attachment and promote Ten4-mediated repulsion. We refer to it in the result section (lines 356 ff).

Regarding the reviewer's comment about the number of experiments for FC and Ten4 nL, we would like to point out that the mutant Ten4 nL stripes are less repulsive than wild type Ten4 for high-expressing Ten4 neurons. Together with the variability observed in the low-Ten4 expressing neurons, it will be difficult to obtain a significant difference between the two conditions. We note that each stripe assay allows the visualization and quantification of a large number of dissociated cells within a single experiment (>200 cells per experiment, as described in published protocols[2], and that each experiment

itself includes at least two biological replicates that are averaged. Therefore, at least n=3 independent experiments already represent a large total number of analyzed cells and corresponds to the standard procedure for stripe assays using dissociated cells. This approach has been used both by us and multiple labs in previous studies[1,3-7].

Importantly, as detailed above, we observe a significant difference between high- and low-Ten4-expressing neurons on wild-type Ten4 stripes, which directly supports our main conclusion. As stated in the result sections (lines 367 ff), the weaker repulsive effect elicited by Ten4 nL could indicate that interaction with Latrophilin contributes to the full repulsive response mediated by Ten4. One possible explanation is that Latrophilin acts in *cis* with Ten4 to modulate repulsion. As Ten4 is a disulphide-bonded *cis* homodimer, it could potentially engage in Lphn *cis* and homophilic Ten4 *trans* interactions by using the two subunits of the dimer). This would be reminiscent of other Lphn receptor systems such as FLRT, where Unc5 co-expressed *in cis* decreased FLRT-Lphn adhesion in HeLa cells[8]. Although it is beyond the scope of this paper to unravel the molecular mechanisms of the signalling cascade by which Ten4-Ten4 *trans* interaction mediates the observed cell repulsion, we have added this point in the discussion as an interesting direction for future work (lines 447 ff: "The signalling mechanisms that determine these different response modes are not understood, but our stripe assay data suggest that co-expressed Latrophilins could be required for the effective repulsive response of high Ten4-expressing cells to external Ten4".).

Original concern 6) Other cell adhesion molecules, such as N-cadherin (CDH2), have been shown to play a role in cortical neuronal migration. How does the present adhesion system cooperate with other cell adhesion molecules for neuronal migration?

Authors' answer 6) thank you for another excellent comment and helpful reference.... we now include a brief relevant discussion on how Teneurin interactions may functionally interplay with classical adhesion systems, citing the paper on CDH2, in order to hint at these ideas (see lines 420-423).

Comment 3) It would be clearer in this paper to say "cadherins, which play key roles in cortical neuronal migration..." instead of "cadherins, which play key roles in cortical development..." (lines 421-422).

Authors: We have modified the text as suggested.

Original concern 7) Lines 46-47: The authors conclude "The results show how Ten4 orchestrates cortical migration by exclusive structural mechanisms, underpinning the integration of distinct migration programs". For me, it is unclear what the key conclusion of this study is for understanding of the mechanism of cortical neuronal migration.

Authors' answer 6) ... The key conclusion is that, Teneurin4 orchestrates different stages of cortical migration by using a structural/functional switch between high-affinity Lphn interactions and low-affinity homophilic interactions...

Comment 4) This would be clearer to conclude in Abstract.

Authors: We agree and have revised the Abstract text accordingly.

Reviewer #5 (Remarks to the Author):

I co-reviewed this manuscript with one of the reviewers who provided the listed reports. This is part of the Nature Communications initiative to facilitate training in peer review and to provide appropriate recognition for Early Career Researchers who co-review manuscripts.

Authors: We thank the reviewer for their contribution to the review process.

References:

1. del Toro, D., Carrasquero-Ordaz, M.A., Chu, A., Ruff, T., Shahin, M., Jackson, V.A., Chavent, M., Berbeira-Santana, M., Seyit-Bremer, G., Brignani, S., et al. (2020). Structural Basis of Teneurin-Latrophilin Interaction in Repulsive Guidance of Migrating Neurons. Cell 180, 323-339.e19. https://doi.org/10.1016/j.cell.2019.12.014.
2. Yamagishi S, Kesavamoorthy G, Bastmeyer M, Sato K. Stripe Assay to Study the Attractive or Repulsive Activity of a Protein Substrate Using Dissociated Hippocampal Neurons. J Vis Exp. 2016 Jun 19;(112):54096.https://doi.org/10.3791/54096
3. Yamagishi S, Hampel F, Hata K, Del Toro D, Schwark M, Kvachnina E, Bastmeyer M, Yamashita T, Tarabykin V, Klein R, Egea J. FLRT2 and FLRT3 act as repulsive guidance cues for Unc5-positive neurons. EMBO J. 2011 Jun 14;30(14):2920-33. https://doi.org/10.1038/emboj.2011.189.
4. Seiradake, E., del Toro, D., Nagel, D., Cop, F., Härtl, R., Ruff, T., Seyit-Bremer, G., Harlos, K., Border, E.C., Acker-Palmer, A., et al. (2014). FLRT Structure: Balancing Repulsion and Cell Adhesion in Cortical and Vascular Development. Neuron 84, 370–385. https://doi.org/10.1016/j.neuron.2014.10.008.
5. Visser JJ, Cheng Y, Perry SC, Chastain AB, Parsa B, Masri SS, Ray TA, Kay JN, Wojtowicz WM. An extracellular biochemical screen reveals that FLRTs and Unc5s mediate neuronal subtype recognition in the retina. Elife. 2015 Dec 2;4:e08149. https://doi.org/10.7554/eLife.08149.

6. Kim HJ, Lee E, Nam M, Chung JK, Joo S, Nam Y, Sun W. Contribution of Extracellular Matrix Component Landscapes in the Adult Subventricular Zone to the Positioning of Neural Stem/Progenitor Cells. Exp Neurobiol. 2021 Aug 31;30(4):275-284. https://doi.org/10.5607/en21012.
7. Marler KJ, Poopalasundaram S, Broom ER, Wentzel C, Drescher U. Pro-neurotrophins secreted from retinal ganglion cell axons are necessary for ephrinA-p75NTR-mediated axon guidance. Neural Dev. 2010 Nov 2;5:30. https://doi.org/10.1186/1749-8104-5-30.
8. Jackson, V.A., Mehmood, S., Chavent, M., Roversi, P., Carrasquero, M., del Toro, D., Seyit-Bremer, G., Ranaivoson, F.M., Comoletti, D., Sansom, M.S.P.M., et al. (2016). Super-complexes of adhesion GPCRs and neural guidance receptors. Nat Commun 7, 11184. https://doi.org/10.1038/ncomms11184.
